# Acceptance and trust in AI-generated exercise plans among recreational athletes and quality evaluation by experienced coaches: a pilot study

**DOI:** 10.1186/s13104-025-07172-9

**Published:** 2025-03-13

**Authors:** Felix Wachholz, Stefano Manno, Daniel Schlachter, Nicole Gamper, Martin Schnitzer

**Affiliations:** Department of Sport Science, University of Innsbruck, Fürstenweg 185, Innsbruck, 6020 Austria

**Keywords:** ChatGPT, Artificial intelligence, Digital training, Large language models, Exercise plans

## Abstract

**Objectives:**

Large language models are becoming increasingly significant tools in everyday life, including the context of training and sports. However, the extent to which recreational athletes actually rely on AI-generated training plans and the differences in trust towards these technologies between users and non-users have not yet been investigated. Furthermore, there is a lack of information regarding the current quality of such AI-generated training plans. The aim of this project was to examine how users and non-users differ in their trust towards these technologies and to assess the quality of AI-generated training plans.

**Results:**

In our sample, 54% of the participants trained using a structured training plan, with 25% of those utilizing AI-generated training plans. Users of these AI-based tools exhibited significantly (*p* = 0.030) higher levels of trust in these technologies compared to non-users. The quality of the output from large language models has now reached a level where even professional coaches are often unable to distinguish whether a training plan was AI-generated or created by a human expert. This suggests that AI-generated training plans could potentially match the standards of those developed by experienced coaches, making them a viable option for athletes seeking guidance in their training.

**Supplementary Information:**

The online version contains supplementary material available at 10.1186/s13104-025-07172-9.

## Introduction

From personalized training plans [1] to performance analytics [2], artificial intelligence (AI) technologies and especially large language models (LLMs) are revolutionizing how athletes, both professional and amateur, approach training and development. While these AI-models show potential in supporting athletes’ progress, the significant question remains to what extent recreational athletes adopt these AI-based training systems in their training.

Understanding the factors that might influence the adoption of AI-based sports technologies is crucial for optimizing their use. One useful framework to investigate this is the Technology Acceptance Model (TAM), proposed by [3], which explains how users’ perceptions of technology’s usefulness and ease of use affect their decision to embrace it [4–6]. The TAM was extended by Choung et al. (2023) to include trust and its application to AI. Moreover, the perspectives of experienced coaches on AI-generated training plans are also critical. Coaches, who traditionally tailor training programs to individual athletes based on experience and expertise, might view AI as a supportive tool or as a threat to their professional judgement. Thus, their evaluation of AI-driven training plans, such as those generated by e.g. ChatGPT [7], is essential for understanding the broader implications of AI in sports. This research note aims to address three main areas: the prevalence of training plan uses and especially AI-based training plan usage among recreational athletes, the technology acceptance in an exercise context based on TAM, and the evaluation of an AI-generated training plan by experienced coaches.

## Methods

A mixed-method approach was employed in the current study. First, a quantitative online survey was administered and second, a qualitative approach was utilized to gather insights from skilled and experienced coaches. The study was conducted in full accordance with the ethical principles outlined in the Declaration of Helsinki.

### Questionnaire

Participation in the present study required proficiency in the German language. The survey was specifically targeted at recreational athletes from Innsbruck, Austria. A total of 158 responses were received, of which 39 were excluded due to incomplete questionnaire submissions. This left a final sample of 119 participants. The sample consisted of 62 female participants and 55 male participants. One participant identified as non-binary, and one did not disclose their gender. The average age of the participants was 28.5 ± 7.4 years.

Data collection was conducted using a self-report questionnaire (www.soscisurvey.com), which was made available to participants as an online questionnaire. The distribution of the questionnaire to recreational and non-elite athletes was carried out through private contacts in the sports community, as well as through targeted internet forums. The data were collected over a four-week period from January 4 to January 31, 2024.

The TAM + Trust model was developed by [8] to include trust and its application to AI. The constructs of this study were translated into German and thematically adapted to AI systems for training planning. The constructs *Usefulness* (*N* = 5), *User-friendliness* (*N* = 5), *Intention to Use* (*N* = 3) and *Attitude* (*N* = 4) were measured using a five item scale translated into German from Choung et al. (2023). The construct *Trust* (*N* = 7) was translated into German and thematically adapted to AI systems for training plans [9]. The items of the constructs *Usefulness*, *User-friendliness* and *Intention to Use* were rephrased for both, users and non-users. Individuals using an AI-generated training plan received specifically formulated items, while those who did not use a training plan or did not use an AI-generated plan were presented with a hypothetical item. The full questionnaire can be found in the supplementary material 1 and 1a. Respondents rated these items on a five-point Likert scale (1 = strongly disagree to 5 = strongly agree).

### Semi-structured interviews

To evaluate training plan quality by experts, a literature-based expert interview guide was developed and six interviews were conducted. The selection of interview participants was limited to coaches from elite sports and all interviews were held by the same interviewer. The average professional experience of the interviewed coaches was 12.2 ± 7.4 years, and all had completed a degree in sports science, along with various additional coaching certifications. All six interviews were conducted between December 2023 and January 2024. During the interviews care was taken to ensure that the respondents had the opportunity to speak freely while still answering all the questions to ensure the best possible comparability of results.

After the introductory questions about the coach’s background in training planning and qualifications, the main part of the interview focused on specific questions regarding the creation of training plans. At the end of each interview, respondents were asked to provide a quality assessment of the two different training plans, as were the participants in the questionnaires. Interviewees were informed that the training plans were designed for a beginner runner who was preparing to run a half marathon for the first time. The interviews lasted on average 47.5 ± 7.2 min. The full version of the expert interview guide (Supplementary material 2) and the transcripts (Supplementary material 3) can be found in the supplementary material.

Before each interview, written consent was obtained from the interview participants regarding their agreement to have the conversation recorded as an audio file. After recording, the audio files were transcribed and analyzed using qualitative content analysis [10], and transcriptions were processed with the software MAXQDA 2024 (VERBI Software, 2024), before being coded for analysis. After the transcription of the interviews, paraphrasing was carried out to clarify and concisely express the interviewees’ statements.

### Creation of training plans

The human-created training plan is a publicly available, 12-week half marathon program created by Terrence Mahon, which can be accessed on the Runtastic website. The program was designed for running beginners and Terrence Mahon is an American middle- and long-distance running coach with years of expertise in creating training plans.

The AI training plan to be shown to participants and experts was created using ChatGPT Version 3.5 [7] with a prompt telling to create the plan as a coach for middle and long-distance running. Based on the human-created program, it was prompted to cover 12 weeks and contain simple workout descriptions. The person asking for it would be a running beginner of average fitness and it would be his first half marathon (the original prompt can be found in the Supplementary material 4).

### Statistical analysis of the questionnaire

To assess the internal consistency of the questionnaire scales, Cronbach’s alpha was employed. This statistical measure was chosen to evaluate the reliability of the items within each scale, ensuring that they consistently measured the intended constructs. Given that the data did not follow a normal distribution, non-parametric tests were conducted using MannWhitney-U tests to compare differences between groups. This test was selected due to its suitability for analyzing ordinal data or data that deviate from normality. To compare the AI-generated and human-created training plan a Wilcoxon-test was used. Effect sizes were calculated and interpreted using Cohen’s *d* to determine the magnitude of the differences observed [11]. No statistical analysis was performed on the semi-structured interviews, apart from the application of descriptive statistics.

## Results

### Questionnaire

Of the 119 participants, 74,1% reported to exercise 3–4 times or more per week. More than the half, specifically 65 participants, reported to train with a training plan, while 54 indicated they did not use a plan. Among those who used a training plan, 16 reported using an AI-generated plan. There were no differences in the types of sports practiced regarding whether or not a training plan was used. AI-generated training plans were most commonly used by runners (*N* = 3), cyclists (*N* = 1), and triathletes (*N* = 1). Other AI-based offerings, such as Enduco, PerfectPace, or Garmin Coach, were also primarily used by runners, cyclists, and triathletes. Freeletics was reported to be used by 2 strength athletes. Regarding gender, ten women, five men, and one non-binary person indicated using AI-based training plans. According to the results of Cronbach’s alpha analysis, the questionnaire items demonstrated internal consistency.

**Fig. 1:**
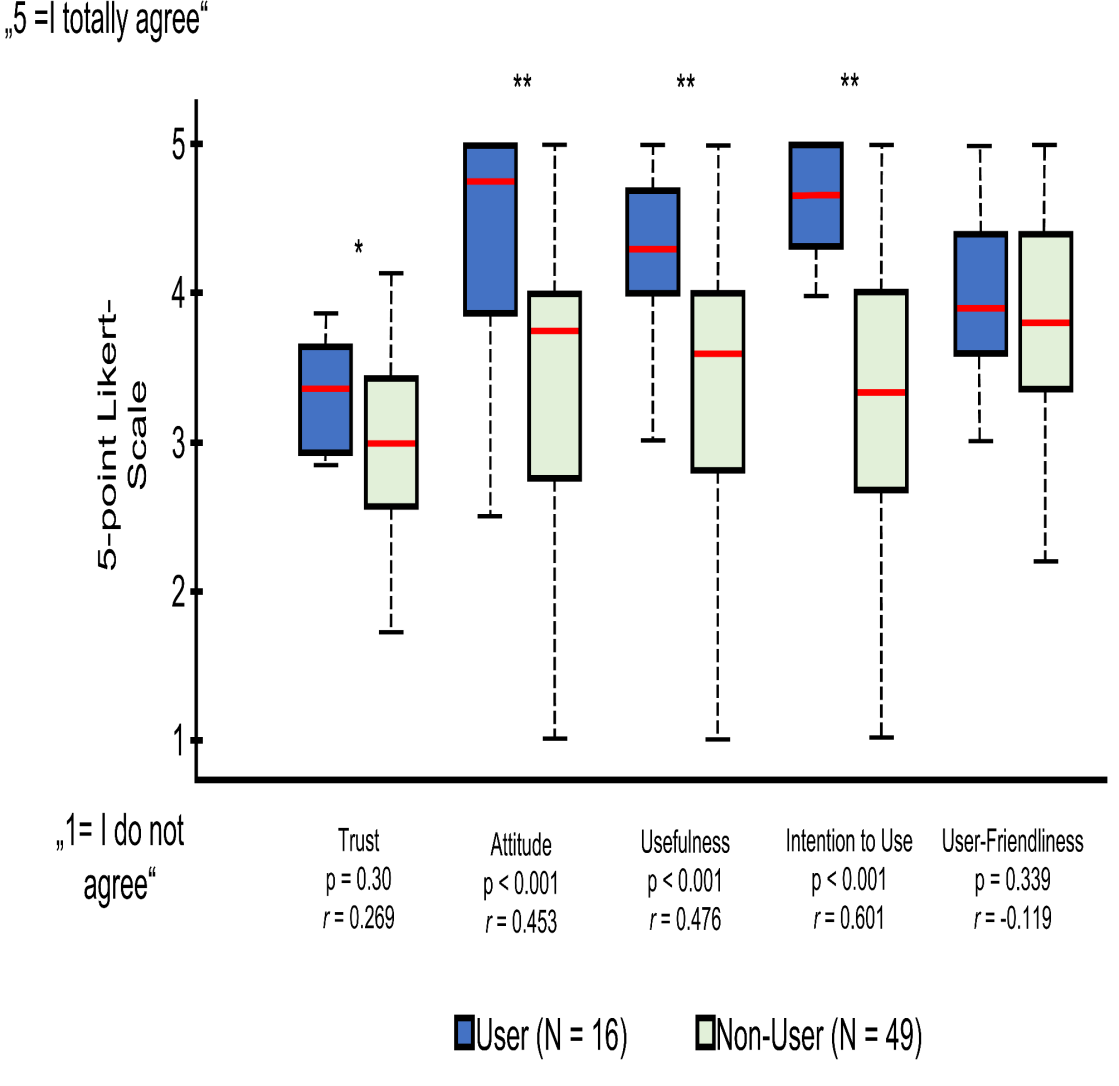
Differences between the scales *Trust*, *Attitude*, *Usefulness*, *Intention to Use* and *User-Friendliness* visualized. The asterisks indicate significant differences (*p* < 0.05; *) or highly significant differences (*p* < 0.001, **). The correlation coefficient specifies the magnitude of the effects, small effect: *r* = 0.1 to 0.3, medium effect: *r* = 0.3 to 0.5 and large effect: *r* > 0.5

Statistical analysis revealed significant differences between users and non-users of AI-generated exercise plans, with users scoring notably higher regarding *Trust*, *Attitude*, *Usefulness* and *Intention to Use*. However, *UserFriendliness* did not show significant differences. Results are visualized in Fig. 1.

When comparing the two presented training plans - one AI-generated and one human-designed - it was found that the AI-generated training plan (3.73 ± 1.1) received significantly higher *Trust* (*p* = 0.002, *r* = -0.291) than the human-designed plan (3.36 ± 1.1). When this comparison was stratified by training plan users and non-users, a significantly higher level of *Trust* (*p* < 0.001, *r* = -0.435) in the AI-generated training plan (3.89 ± 1.1) was also observed among training plan users compared to the human-designed plan (3.31 ± 1.1). However, this difference was not present among individuals who did not use a training plan (*p* = 0.440, *r* = -0.105).

### Semi-structured interviews

All interviewees agreed that the AI-generated (Training Plan 1) plan is better suited for the athlete, as it is “easier for the athlete to understand” (IP2, line 121) and “more concise” (IP3, 194). “Training Plan 1 is more suitable given that the athlete is preparing for their first half marathon, as it is more straightforward and easier to understand than Training Plan 2” (IP5, 173–175). With Training Plan 1, “there is little room for error; it represents a general average of many plans you would find on the internet” (IP6, 201–203).

On the other hand, Training Plan 2 (created by an expert running coach) “is presented in more detail” (IP1, 114) and contains “a lot of information, perhaps even too much” (IP2, 116). Additionally, “the option of ‘or rest day’ could confuse the athlete, as they might not know whether they should complete the session or not” (IP4, 181–183). Participant 6 also pointed out the high training volumes required in Training Plan 2: “If someone likes this plan, they must already be in such good shape that they don’t really need to prepare for the half marathon, as they would already be fit enough without this plan” (IP6, 189–190).

At the end of each interview, participants were asked to identify which of the two training plans had been generated by AI. Out of the six interviewees, four correctly identified that Training Plan 1 was created using AI.

## Discussion

The survey-results from this sample indicate that in recreational sports, approximately every second person trains according to a training plan. Furthermore, the use of AI-generated training plans is not yet widespread. However, there are significant differences in relation to the acceptance based on the TAM + Trust [3, 9] depending on whether individuals are users or non-users of such technologies, with users already demonstrating higher *Trust*, a more positive *Attitude*, and greater *Usefulness* and *Intention to Use* compared to non-users. This finding aligns with the literature, which shows that user trust is a crucial factor for the actual utilization of AI systems [8, 9]. Companies offering AI solutions should prioritize building this trust to enhance user engagement and maximize their technology’s utilization. The results, combined with the findings from expert interviews, also suggest that the use of AI-generated training plans in recreational sports could be a cost-effective way to access training plans of adequate quality, which is also consistent with existing literature [1, 12]. Particularly when the alternative is to train without a plan or understanding of training management, amateur athletes could be at least partially protected from overtraining or injury. The fact that even experts were sometimes unable to distinguish whether a training plan was AI-generated or created by a human expert highlights the current advancements of LLMs [12] like ChatGPT [7].

### Limitations

When interpreting the results, several limitations of the study should be considered. Most importantly, it should be noted that the highest quality coaching in the trainer-athlete context is likely still best achieved through personalized guidance, particularly when various objective parameters are incorporated into the training design. Nonetheless, the AI-generated training plans demonstrate potential in providing at least a structured approach, which is likely still superior to training without any specific guidance. The data collected is based on self-reports, which may lead to potential biases due to social desirability or subjective perceptions. Additionally, the sample may not be representative of all athletes and coaches, especially regarding the use of AI-generated training plans. The number of users of AI-generated training plans in the study was very low, with only 16 individuals. However, the effect size presented to be mostly moderate or strong. Nonetheless, a more appropriate logistic regression analysis to assess the impact of different scales on the use of AI-generated training programs could not be reliably conducted due to the limited sample size.

Moreover, this suggests that AI-generated training plans are not yet widely adopted. It should also be noted that the training plan generated by ChatGPT was formulated in simpler language. Participants may have rated their trust in the training plan higher due to its easier comprehensibility and more visually appealing presentation. Nevertheless, there is a trend indicating that athletes may trust an AI-generated training plan as much as that of a professional coach. Since only three institutions were included in the interviews, some statements regarding the use of AI in high-performance sports training overlapped. Future research should ensure that a broader range of organizations is surveyed to generate a more comprehensive set of perspectives.

## Electronic supplementary material

Below is the link to the electronic supplementary material.

Supplementary Material 1

Supplementary Material 1a

Supplementary Material 2

Supplementary Material 3

Supplementary Material 4

## Data Availability

The datasets used and/or analyzed during the current study are available from the corresponding author on reasonable request.

## Abbreviations

AI: Artificial intelligence
LLMs: Large language models
TAM: Technology Acceptance Model
