# Supplementary material for: Acceptance and trust in AI-generated exercise plans among recreational athletes and quality evaluation by experienced coaches: a pilot study
Source: BMC Res Notes. 2025 Mar 13;18:112. doi: 10.1186/s13104-025-07172-9 (PMC11908068; doi:10.1186/s13104-025-07172-9)
Supplement: Supplementary file 1 — Supplementary Material 1 [file 13104_2025_7172_MOESM1_ESM.pdf]

Fragebogen

Seite 1 | Intro

universität innsbruck

0% ausgefüllt

Liebe*r Untersuchungsteilnehmer*in!

Im Rahmen meiner Abschlussarbeit des Studiums Sportmanagement an der Universität Innsbruck beschäftige ich mich damit, inwiefern Sportler*innen aus verschiedenen Bereichen Trainingsplänen vertrauen, die mittels künstlicher Intelligenz erstellt wurden. Für die Analyse ist Ihre Sicht als Sportler*in sehr wichtig!

Die Befragung ist anonym! Alle Ihre Angaben werden streng vertraulich behandelt, es können keine Rückschlüsse auf einzelne Personen gezogen werden. Eine individuelle Auswertung ist aufgrund der Anonymisierung nicht möglich.

Im Voraus vielen Dank dafür, dass Sie sich die Zeit zur Beantwortung der Fragen nehmen!

Seite 2 | Sportbezogene Fragen

Welcher Sportart gehen Sie hauptsächlich nach?

Bitte geben Sie die Sportart an, die sie hauptsächlich durchführen. Wenn Sie mehrere Sportarten ausüben, wählen Sie die Sportart für die Sie am meisten trainieren.

- ◯ Laufen (Straße, Trail, ...)
- ◯ Radfahren (MTB, Rennrad, ...)
- ◯ Schwimmen
- ◯ Triathlon
- ◯ Leichtathletik
- ◯ Krafttraining
- ◯ Klettern
- ◯ Kampfsport
- ◯ Ski (Alpin, Langlauf, Freeride, Mountaineering, ...)
- ◯ Snowboard
- ◯ Fußball
- ◯ Handball
- ◯ Andere: ____________
- ◯ keine Angabe

Wie oft pro Woche treiben Sie durchschnittlich Sport?

Beziehen Sie die Anzahl bitte auf die Trainingseinheit/en.

- ◯ weniger als 1 mal pro Woche
- ◯ 1 bis 2 mal pro Woche
- ◯ 3 bis 4 mal pro Woche
- ◯ 5 bis 6 mal pro Woche
- ◯ mehr als 6 mal pro Woche
- ◯ keine Angabe

Wie lange trainieren Sie durchschnittlich pro Trainingseinheit?

- ○ weniger als 30 Minuten
- ○ 30 – 60 Minuten
- ○ 60 – 90 Minuten
- ○ 90 – 120 Minuten
- ○ mehr als 120 Minuten
- ○ keine Angabe

Trainieren Sie nach einem Trainingsplan?

- ○ Ja
- ○ Nein

Seite 3 | KI (Trainingsplan)

Definition von Künstlicher Intelligenz

Im Folgenden wird der Begriff Künstliche Intelligenz (KI) definiert, wie er in dieser Studie verwendet wird:

Ein KI-System ist ein maschinenbasiertes System, das Empfehlungen geben, Prognosen erstellen oder Entscheidungen treffen kann.

Es verwendet maschinelle oder menschliche Eingaben, um eine reale oder virtuelle Umgebung zu erfassen. Auf dieser Grundlage werden Modelle erstellt und Informationen oder Handlungsoptionen ermittelt. KI-Systeme können mit einem unterschiedlichen Grad an Autonomie ausgestattet sein.

Diese Funktionen werden auch bei der Erstellung von Trainingsplänen im Sport genutzt.

Seite 4

Wird nur angezeigt bei vorheriger Antwort mit „Ja".

Verwenden Sie momentan einen Trainingsplan, der mittels Künstlicher Intelligenz erstellt wurde?

- ○ Ja
- ○ Nein

Seite 5

Wird nur angezeigt bei vorheriger Antwort mit „Ja".

Verwenden Sie eines der folgenden Tools zur Erstellung eines KI-generierten Trainingsplans?
Wenn Sie das Tool für andere Zwecke – wie die Aufzeichnung von Leistungsdaten – nutzen, so kreuzen Sie es bitte NICHT an!

- ☐ ChatGPT
- ☐ Enduco
- ☐ Freeletics
- ☐ Strava
- ☐ Twaiv
- ☐ VIPerform
- ☐ Andere: ____________

Seite 6 | TAM

Einstellung

Bitte geben Sie an, inwiefern Sie den folgenden Aussagen zustimmen.

Ich stehe KI-Systemen zur Trainingsplanung positiv gegenüber.	stimme überhaupt nicht zu	○	○	○	○	○	stimme vollkommen zu
Ich empfinde die Nutzung von KI-Systemen zur Trainingsplanung als angenehm.	stimme überhaupt nicht zu	○	○	○	○	○	stimme vollkommen zu
Die Verwendung von KI-Systemen zur Trainingsplanung ist eine gute Idee.	stimme überhaupt nicht zu	○	○	○	○	○	stimme vollkommen zu
Die Verwendung von KI-Systemen zur Trainingsplanung ist eine intelligente Art, um die Trainingsplanung durchzuführen.	stimme überhaupt nicht zu	○	○	○	○	○	stimme vollkommen zu

Wahrgenommene Benutzerfreundlichkeit

Nutzer

Bitte geben Sie an, inwiefern Sie den folgenden Aussagen zustimmen.

Ich finde die Nutzung von KI-Systemen zur Trainingsplanung einfach.	stimme überhaupt nicht zu	○	○	○	○	○	stimme vollkommen zu
Ich finde es einfach, mit KI-Systemen meine Trainingsplanung durchzuführen.	stimme überhaupt nicht zu	○	○	○	○	○	stimme vollkommen zu
Die Trainingsplanung mit KI-Systemen ist klar und verständlich.	stimme überhaupt nicht zu	○	○	○	○	○	stimme vollkommen zu
Ich bin kompetent in der Trainingsplanung mit KI-Systemen.	stimme überhaupt nicht zu	○	○	○	○	○	stimme vollkommen zu
Ich finde KI-Systeme zur Trainingsplanung einfach zu bedienen.	stimme überhaupt nicht zu	○	○	○	○	○	stimme vollkommen zu

Nicht Nutzer

Bitte geben Sie an, inwiefern Sie den folgenden Aussagen zustimmen.

Die Nutzung von KI-Systemen zur Trainingsplanung wäre für mich einfach zu erlernen.	stimme überhaupt nicht zu	○	○	○	○	○	stimme vollkommen zu
Ich fände es einfach, mit KI-Systemen meine Trainingsplanung durchzuführen.	stimme überhaupt nicht zu	○	○	○	○	○	stimme vollkommen zu
Die Trainingsplanung mit KI-Systemen wäre klar und verständlich.	stimme überhaupt nicht zu	○	○	○	○	○	stimme vollkommen zu
Es wäre einfach für mich, kompetent in der Trainingsplanung mit KI-Systemen zu werden.	stimme überhaupt nicht zu	○	○	○	○	○	stimme vollkommen zu
Ich fände KI-Systeme zur Trainingsplanung einfach zu bedienen.	stimme überhaupt nicht zu	○	○	○	○	○	stimme vollkommen zu

Wahrgenommene Nützlichkeit

Nutzer

Bitte geben Sie an, inwiefern Sie den folgenden Aussagen zustimmen.

KI-Systeme helfen mir dabei, meine Trainingsplanung schneller zu erledigen.	stimme überhaupt nicht zu	○	○	○	○	○	stimme vollkommen zu
KI-Systeme verbessern meine Leistung bei der Trainingsplanung.	stimme überhaupt nicht zu	○	○	○	○	○	stimme vollkommen zu
KI-Systeme steigern meine Produktivität bei der Trainingsplanung.	stimme überhaupt nicht zu	○	○	○	○	○	stimme vollkommen zu
KI-Systeme erhöhen meine Effektivität bei der Trainingsplanung.	stimme überhaupt nicht zu	○	○	○	○	○	stimme vollkommen zu
Ich finde die Verwendung von KI-Systeme zur Trainingsplanung nützlich.	stimme überhaupt nicht zu	○	○	○	○	○	stimme vollkommen zu

Nicht Nutzer

Bitte geben Sie an, inwiefern Sie den folgenden Aussagen zustimmen.

KI-Systeme würden mir dabei helfen meine Trainingsplanung schneller zu erledigen.	stimme überhaupt nicht zu	○	○	○	○	○	stimme vollkommen zu
KI-Systeme würden meine Leistung bei der Trainingsplanung verbessern.	stimme überhaupt nicht zu	○	○	○	○	○	stimme vollkommen zu
KI-Systeme würden meine Produktivität bei der Trainigsplanung steigern.	stimme überhaupt nicht zu	○	○	○	○	○	stimme vollkommen zu
KI-Systeme würden meine Effektivität bei der Trainingsplanung erhöhen.	stimme überhaupt nicht zu	○	○	○	○	○	stimme vollkommen zu
Ich fände die Verwendung von KI-Systemen zur Trainingsplanung nützlich.	stimme überhaupt nicht zu	○	○	○	○	○	stimme vollkommen zu

Nutzungsintention

Nutzer

Bitte geben Sie an, inwiefern Sie den folgenden Aussagen zustimmen.

Ich beabsichtige, KI-Systeme zur Trainingsplanung weiterhin zu nutzen.	stimme überhaupt nicht zu	○ ○ ○ ○ ○	stimme vollkommen zu
Ich treffe Entscheidungen zur Trainingsplanung basierend auf Empfehlungen von KI-Systemen.	stimme überhaupt nicht zu	○ ○ ○ ○ ○	stimme vollkommen zu
KI-Systeme zur Trainingsplanung zu nutzen ist etwas, dass ich in Zukunft weiterhin tun werde.	stimme überhaupt nicht zu	○ ○ ○ ○ ○	stimme vollkommen zu

Nicht Nutzer

Bitte geben Sie an, inwiefern Sie den folgenden Aussagen zustimmen.

Ich beabsichtige, KI-Systeme zur Trainingsplanung in Zukunft zu nutzen.	stimme überhaupt nicht zu	○ ○ ○ ○ ○	stimme vollkommen zu
Ich beabsichtige, Entscheidungen zur Trainingsplanung basierend auf Empfehlungen von KI-Systemen zu treffen.	stimme überhaupt nicht zu	○ ○ ○ ○ ○	stimme vollkommen zu
KI-Systeme zur Trainingsplanung zu nutzen ist etwas, dass ich in Zukunft tun würde.	stimme überhaupt nicht zu	○ ○ ○ ○ ○	stimme vollkommen zu

Seite 7 | Vertrauen

Bitte geben Sie an, inwiefern Sie den folgenden Aussagen zustimmen.

KI-Systeme für die Trainingsplanung sind kompetent in der Bereitstellung von Informationen und Beratung, die ich brauche.	stimme überhaupt nicht zu	○ ○ ○ ○ ○	stimme vollkommen zu
KI-Systeme für die Trainingsplanung sind zuverlässig und liefern konsistente und verlässliche Informationen.	stimme überhaupt nicht zu	○ ○ ○ ○ ○	stimme vollkommen zu
KI-Systeme für die Trainingsplanung sind transparent.	stimme überhaupt nicht zu	○ ○ ○ ○ ○	stimme vollkommen zu
KI-Systeme für die Trainingsplanung sind verlässlich und glaubwürdig.	stimme überhaupt nicht zu	○ ○ ○ ○ ○	stimme vollkommen zu
KI-Systeme für die Trainingsplanung werden ihre Antworten nicht manipulieren und keine negativen Konsequenzen für mich erzeugen.	stimme überhaupt nicht zu	○ ○ ○ ○ ○	stimme vollkommen zu
KI-Systeme für die Trainingsplanung handeln in guter Absicht und sind ehrlich zu mir.	stimme überhaupt nicht zu	○ ○ ○ ○ ○	stimme vollkommen zu
KI-Systeme für die Trainingsplanung sind sicher und schützen meine Privatsphäre und vertrauliche Informationen	stimme überhaupt nicht zu	○ ○ ○ ○ ○	stimme vollkommen zu

Bitte geben Sie an, inwiefern Sie den folgenden Aussagen zustimmen.

Ich vertraue Trainingsplänen, die mittels künstlicher Intelligenz erstellt wurden.	stimme überhaupt nicht zu	○ ○ ○ ○ ○	stimme vollkommen zu
Ich vertraue KI-Systemen für die Trainingsplanung.	stimme überhaupt nicht zu	○ ○ ○ ○ ○	stimme vollkommen zu

Seite 8 - 10 | Vergleich Trainingspläne

Trainingsplan Beispiel

Im Folgenden werden Ihnen **zwei** Trainingspläne präsentiert.

Es handelt sich jeweils um einen 12-wöchigen Halbmarathon-Trainingsplan, von dem Ihnen einfachheitshalber die ersten beiden Wochen gezeigt werden.

Diese sind für eine Person ausgelegt, die:

- durchschnittlich sportlich ist und
- ihren ersten Halbmarathon läuft und
- zum Ziel hat diesen zu schaffen.

Lesen Sie sich die Trainingspläne bitte durch und beantworten Sie die darunter stehende Frage.

Randomisierung Reihenfolge Trainingsplan X und Y

Trainingsplan X

Woche 1-2	Aufwärmen	Workout Beschreibung	Abkühlen
**Montag**	5 Minuten aktives Dehnen	Lockeres Laufen bei 60 bis 65 % der max. Herzfrequenz – 20 bis 30 Minuten	5 Minuten Dehnen
**Dienstag**	5 Minuten aktives Dehnen	Lockeres Laufen oder Crosstraining bei 60 bis 65 % der max. Herzfrequenz – 20 bis 30 Minuten oder Ruhetag	5 Minuten Dehnen
**Mittwoch**	2 bis 3 km,<br>5 Minuten aktives Dehnen	6 bis 8 x 100-m-Bergsprints, zur Regeneration jeweils zurück zum Start joggen/gehen,<br>3 bis 4 Minuten Pause oder joggen zu ebenem Gelände,<br>6 bis 8 x 400 m bei Halbmarathon- bis 10-km-Pace (oder 85 bis 90 % der max. Herzfrequenz) mit jeweils 60 bis 75 Sekunden Pause.<br>**Wiederhole in Woche 2 die 400-m-Intervalle 10 - 12 mal*	2 bis 3 km,<br>5 Minuten Dehnen
**Donnerstag**	Ruhetag		
**Freitag**	5 Minuten aktives Dehnen	Lockeres Laufen bei 60 bis 65 % der max. Herzfrequenz – 30 bis 40 Minuten	5 Minuten Dehnen
**Samstag**	2 bis 3 km,<br>5 Minuten aktives Dehnen	4 bis 6 x 100-m-Sprints bei 90 bis 95 % der Höchstleistung mit 30 - 45 Sekunden Pause,<br>dann 3 Minuten Pause,<br>3 bis 6 km Tempolauf bei Marathon-Pace (80 % der max. Herzfrequenz)<br>**Erhöhe den Tempolauf in Woche 2 auf 5–8 km*	2 bis 3 km,<br>5 Minuten Dehnen
**Sonntag**	Ruhetag		

Bitte geben Sie an, inwiefern Sie der folgenden Aussage zustimmen.

Ich vertraue diesem Trainingsplan.	stimme überhaupt nicht zu	○ ○ ○ ○ ○	stimme vollkommen zu

Trainingsplan Y

Woche 1-2	Aufwärmen	Workout Beschreibung	Abkühlen
Montag	10 Minuten leichtes Laufen	Easy Run: 3 km in gemütlichem Tempo (60-70% deiner maximalen Herzfrequenz)	5-10 Minuten Gehen und Dehnen
Dienstag	10 Minuten leichtes Laufen	Intervalltraining: 4x400 Meter schnelles Laufen (80-90% deiner maximalen Herzfrequenz) mit 2 Minuten Gehpause dazwischen	5-10 Minuten Gehen und Dehnen
Mittwoch	Ruhetag oder leichtes Crosstraining (z.B. Radfahren oder Schwimmen)		
Donnerstag	10 Minuten leichtes Laufen	Tempo Run: 5 km moderates Tempo (70-80% deiner maximalen Herzfrequenz)	5-10 Minuten Gehen und Dehnen
Freitag	Ruhetag oder leichtes Crosstraining		
Samstag	10 Minuten leichtes Laufen	Long Run: 8 km in gemütlichem Tempo (60-70% deiner maximalen Herzfrequenz)	10-15 Minuten Gehen und Dehnen
Sonntag	Ruhetag		

Bitte geben Sie an, inwiefern Sie der folgenden Aussage zustimmen.

Ich vertraue diesem Trainingsplan. stimme überhaupt nicht zu ○ ○ ○ ○ ○ stimme vollkommen zu

Seite 11 | Soziodemografische Daten

Welchem Geschlecht fühlen Sie sich zugehörig?

- ○ Männlich
- ○ Weiblich
- ○ Divers
- ○ keine Angabe

Wie alt sind Sie?
Bitte geben Sie ihr Alter in Zahlen an.

Alter: ☐ keine Angabe

Welches ist Ihre höchste abgeschlossene Ausbildung?

- ○ Pflichtschule
- ○ Berufsschule (Lehre)
- ○ Weiterführende Schule ohne Matura / Abitur (Handelsschule, Fachschule, ...)
- ○ Weiterführende Schule mit Matura / Abitur (Gymnasium, HAK, HTL, ...)
- ○ Universität: Bachelor
- ○ Universität: Master, PHD
- ○ Anderes
- ○ keine Angabe

Welcher Tätigkeit gehen Sie momentan hauptsächlich nach?

- ◯ Schüler*in
- ◯ Student*in
- ◯ Erwerbstätig (weniger als 20 h/Woche)
- ◯ Erwerbstätig (mehr als 20 h/Woche)
- ◯ Ohne Beschäftigung
- ◯ Anderes

- ◯ keine Angabe

Wo befindet sich momentan Ihr Lebensmittelpunkt?

- ◯ Österreich
- ◯ Deutschland
- ◯ Italien
- ◯ Anderes

- ◯ keine Angabe

Seite 12 | Abschluss

universität innsbruck

Vielen Dank für Ihre Teilnahme!

Sie sind am Ende des Fragebogens angekommen.

Ich möchte mich herzlich bei Ihnen für das Beantworten der Fragen und die damit verbundene Unterstützung meiner Abschlussarbeit bedanken.

Alles Gute!

Ihre Antworten wurden gespeichert, Sie können das Browser-Fenster nun schließen.
