# Supplementary material for: Acceptance and trust in AI-generated exercise plans among recreational athletes and quality evaluation by experienced coaches: a pilot study
Source: BMC Res Notes. 2025 Mar 13;18:112. doi: 10.1186/s13104-025-07172-9 (PMC11908068; doi:10.1186/s13104-025-07172-9)
Supplement: Supplementary file 2 — Supplementary Material 1a [file 13104_2025_7172_MOESM2_ESM.pdf]

„Stelle dir vor du bist im Bereich Mittel- und Langstreckenlauf einer der besten Coaches weltweit: Erstelle mir einen 12-wöchigen Halbmarathon-Trainingsplan mit einfachen Workout-Beschreibungen, um für mich das Beste aus meinem Training herauszuholen und den Halbmarathon zu schaffen, während ich gleichzeitig nur so wenig Zeit wie möglich damit verbringe. Ich bin durchschnittlich sportlich und es ist mein erster Halbmarathon. Der Trainingsplan soll so strukturiert sein, dass jede Einheit folgende Teile beinhaltet:

- Aufwärmen (wenn für das Workout erforderlich)
- Workout, mit Angabe der angestrebten Herzfrequenzbereiche
- Abkühlen (wenn für das Workout erforderlich)

Kategorisiere den Trainingsplan nach Woche 1-2, Woche 3-5, Woche 6-8, Woche 9-11, Woche 12.“

Translated:

“Imagine you are one of the best coaches in the world for middle- and long-distance running: Create a 12-week half marathon training plan with simple workout descriptions to help me get the most out of my training and successfully complete the half marathon while spending as little time as possible on it. I am of average fitness, and this is my first half marathon. The training plan should be structured so that each session includes the following parts:

- Warm-up (if required for the workout)
- Workout, with target heart rate zones specified
- Cool-down (if required for the workout)

Please categorize the training plan into: Week 1-2, Week 3-5, Week 6-8, Week 9-11, Week 12.”
