# Supplementary material for: Acceptance and trust in AI-generated exercise plans among recreational athletes and quality evaluation by experienced coaches: a pilot study
Source: BMC Res Notes. 2025 Mar 13;18:112. doi: 10.1186/s13104-025-07172-9 (PMC11908068; doi:10.1186/s13104-025-07172-9)
Supplement: Supplementary file 3 — Supplementary Material 2 [file 13104_2025_7172_MOESM3_ESM.pdf]

Leitfaden: Erstellung von Trainingsplänen und Evaluierung durch Expert*innen

Interviewpartner*in:

Dauer:

Einwilligungserklärung:

Ich erkläre hiermit mein Einverständnis zur Nutzung der personenbezogenen Daten, die im Rahmen des folgenden Gesprächs erhoben wurden. Die Daten werden im Rahmen eines mündlichen Gesprächs erhoben, das mit einem Aufnahmegerät aufgezeichnet wurde. Zum Zwecke der Datenanalyse werden die mündlich erhobenen Daten verschriftlicht (Transkription), wobei die Daten anonymisiert werden. Eine Identifizierung der interviewten Person ist somit ausgeschlossen.

Kontaktdaten, die eine Identifizierung der interviewten Person zu einem späteren Zeitpunkt ermöglichen würden, werden aus Dokumentationsgründen in einem separaten Schriftstück lediglich den Gutachter*innen der wissenschaftlichen Ausarbeitung zur Verfügung gestellt. Nach dem Abschluss des Projekts werden diese Daten gelöscht.

Der Speicherung der personenbezogenen Daten zu Dokumentationszwecken kann durch die interviewte Person jederzeit widersprochen werden. Die Teilnahme an dem Gespräch erfolgt freiwillig. Das Gespräch kann zu jedem Zeitpunkt abgebrochen werden. Das Einverständnis zur Aufzeichnung und Weiterverwendung der Daten kann jederzeit widerrufen werden.

____

Name, Vorname

____

Unterschrift

____

Datum, Ort

universität innsbruck

Themengebiet	Leitfrage	**Checkliste**<br>Wurde die Frage vollständig beantwortet?<br>*Wenn nicht, nochmal nachfragen*	**Zusätzliche Fragen/Aufrechterhaltungsfragen**<br>- *Können Sie dazu etwas mehr erzählen?*<br>- *Haben Sie dazu ein Beispiel?*
Einleitung	- Kurze Vorstellung meinerseits<br>- Aufzeigen des Zwecks des Interviews: die Erstellung von Trainingsplänen und die anschließende Evaluierung durch Experten		
Hauptteil:<br><br>Hintergrund des Trainers/ der Trainerin im Bereich der Trainingsplanung	- Wie lange sind Sie bereits als Trainer*in im Leistungssport aktiv?<br>- Wie lange arbeiten Sie bereits in Ihrer Institution?<br>- Welche Qualifikationen haben Sie bereits in Ihrer Laufbahn erlangt?<br>- Was ist Ihr Spezialgebiet/was liegt Ihnen am meisten in Bezug auf die Trainingsplanung?		
Methoden der Trainingsplanung – bevorzugte Methoden zur Erstellung von Trainingsplänen	- Wie ist der Ablauf von der Planung bis hinzu den fertig erstellten Trainingsplänen?<br>Planung:<br>- Informationsbeschaffung<br>- Fokussetzung<br>- Leistungsmerkmale: Kraft, Geschwindigkeit, Ausdauer, Energiebereitstellung (Skerik et al., 2018)<br>- Welche spezifischen Indikatoren und Kennzahlen werden verwendet, um die Trainingspläne zu erstellen?<br>- Werden vorab sportmotorische Tests durchgeführt?		

universität innsbruck

	- Welche digitalen Hilfsmittel werden in der Trainingsplanung verwendet?		
Flexibilität und Anpassungsfähigkeit	- Wie flexibel ist die Anpassung von Trainingsplänen innerhalb eines bestimmten Zeitraums (Vorbereitung, Wettkampfsaison)?	Wenn unflexibel:<br>Was beeinflusst Flexibilität negativ?	
Herausforderungen bei der Evaluierung	- Welche Herausforderungen/Komplikationen bei der Erstellung von Trainingsplänen können auftreten?<br>- Wie können die Herausforderungen bewältigt werden bzw. was wären digitale Lösungsmöglichkeiten?		
Künstliche Intelligenz	- Wie ist Ihre Einstellung künstlicher Intelligenz gegenüber? (pro/contra)<br>- Welche Vorerfahrungen bestehen in Bezug auf künstliche Intelligenz im Sport?<br>- Besteht grundsätzlich Vertrauen künstlicher Intelligenz gegenüber?<br>- Welche Relevanz hat künstliche Intelligenz bisher im Training des Leistungssports?<br>- Welche Anwendung findet künstliche Intelligenz (in welcher Form auch immer) aktuell?<br>- In welchem Bereich sehen Sie den größten Nutzen von künstlicher Intelligenz im Training des Leistungssports?<br>- Wie wird die Entwicklung von künstlicher Intelligenz in Zukunft eingeschätzt?<br>- Künstliche Intelligenz eher als Hilfsmittel oder Ersatz für Trainer*in?<br>- Besteht die Sorge, in Zukunft von künstlicher Intelligenz als Trainer*in ersetzt zu werden?		

	- Wie ist Ihre Einschätzung zu diesen beiden Trainingsplänen?	*Trainingspläne vorlegen*	
Schluss:<br><br>Dank und Verabschiedung	- Möglichkeit für zusätzliche Kommentare oder Fragen für Trainer*in	*Haben Sie noch Punkte, die Sie gerne ansprechen würden?*	

Guide: Creation of Training Plans and Evaluation by Experts

Interviewee:

Duration:

Consent Declaration:

I hereby declare my consent for the use of the personal data collected during the following interview. The data is collected during a verbal conversation recorded with a recording device. For the purpose of data analysis, the verbally collected data will be transcribed, with all data anonymized. Identification of the interviewed person is therefore excluded.

Contact details that could allow the identification of the interviewed person at a later time will be made available, for documentation purposes, only to the reviewers of the scientific work in a separate document. These data will be deleted after the project is completed.
The interviewed person can object to the storage of personal data for documentation purposes at any time. Participation in the interview is voluntary. The interview can be terminated at any time. Consent to the recording and further use of the data can be withdrawn at any time.

____

Name, Surname

____

Signature

____

Date, Location

universität innsbruck

Area Topic	Guidance Question	**Checklist**<br>*Was the question answered completely? If not, ask again.*	**Additional /Maintenance Questions**<br>- *Could you tell me more?*<br>- *Do you have an example?*
Introduction	- Brief introduction of myself<br>- Explaining the purpose of the interview: the creation of training plans and subsequent evaluation by expert		
Main part:<br><br>Background of the coach related to training planing	- How long have you been working as a coach in high-performance sports?<br>- How long have you been working at your institution?<br>- What qualifications have you acquired in your career so far?<br>- What is your area of expertise/what is your focus in training planning?		
Methods of Training Planning – Preferred Methods for Creating Training Plans	- What is the process of the planning towards a ready to use training plan?<br>Planning:<br>- Information gathering<br>- Setting the focus<br>- Performance characteristics: strength, speed, endurance, energy supply (Skerik et al., 2018)<br>- What specific indicators and metrics are used to create the training plans?<br>- Are motor skills tests conducted in advance?		

	- Which digital support is used while creating training plans?		
Flexibility and adaptability	- How flexible is the adaption of training planning within a given timeframe (preparation, competition season, ...)?	If unflexible:<br>What influences flexibility negative?	
Challenges in evaluation	- What challenges/complications can arise when creating training plans?<br>- How can these challenges be addressed, and what digital solutions might help?		
Artificial Intelligence	- What is your attitude towards artificial intelligence (AI)? (pro/con)<br>- What prior experience do you have with artificial intelligence in sports?<br>- Is there a general trust in artificial intelligence?<br>- What relevance does artificial intelligence currently have in elite sports training?<br>- How is artificial intelligence currently applied (in any form)?<br>- In which area do you see the greatest benefit of artificial intelligence in elite sports training?<br>- How is the development of artificial intelligence expected to evolve in the future?<br>- Is artificial intelligence more of a tool or a replacement for a coach?<br>- Is there concern about being replaced by artificial intelligence as a coach in the future?		

	- What is your assessment of these two training plans?	*present trainingplans*	
End:<br><br>Thanks and closing	- Opportunity for additional comments or questions for the coach	*Are there any points you would like to address?*	
