# Supplementary material for: Acceptance and trust in AI-generated exercise plans among recreational athletes and quality evaluation by experienced coaches: a pilot study
Source: BMC Res Notes. 2025 Mar 13;18:112. doi: 10.1186/s13104-025-07172-9 (PMC11908068; doi:10.1186/s13104-025-07172-9)
Supplement: Supplementary file 4 — Supplementary Material 3 [file 13104_2025_7172_MOESM4_ESM.pdf]

Transskripte der geführten Interviews

Interviewpartner 1 (IP 1), 06.12.2023

Ich: Wie lange sind Sie bereits als Trainer*in im Leistungssport aktiv?

IP 1: Am Olympiazentrum seit Februar 2023. Davor war ich noch drei Jahre selbstständig als Trainer im Powerlifting tätig und mache das aktuell auch noch nebenher.

Ich: Wie lange arbeiten Sie bereits in Ihrer Institution?

IP 1: Seit Februar 2023. Ich betreue Sportarten wie Paraski, Ringen, Segeln, Skinachwuchs, Skilangläufer.

Ich: Welche Qualifikationen haben Sie bereits in Ihrer Laufbahn erlangt?

IP 1: Ich habe Sportwissenschaften im Bachelor in Konstanz studiert und den Master Sportwissenschaften in Innsbruck. Ich war selber lange im Klettern, Mountainbike und Powerlifting im Leistungssport aktiv. Ich habe den C-Trainer im Mountainbiken, C-Trainer Snowboarden, Fitness B-Lizenz abgeschlossen.

Ich: Was ist Ihr Spezialgebiet/was liegt Ihnen am meisten in Bezug auf die Trainingsplanung?

IP 1: Maximalkraft und Hypertrophie sind meine Spezialgebiete in Bezug auf die Trainingsplanung.

Ich: Wie ist der Ablauf von der Planung bis hinzu den fertig erstellten Trainingsplänen?

IP 1: Das kommt natürlich ganz darauf an in welchem Umfeld der Sportler oder die Sportlerin bevor sie zu uns gekommen sind, waren. Oftmals haben die Athleten bereits Spezialtrainer, eventuell haben Sportler auch Trainer die zum Beispiel das Ausdauertraining übernehmen. Das heißt man muss im Vorhinein abklären, was es für Trainings bereits gibt. Wir setzen uns dann zuerst mit dem Athleten und danach mit den Trainern zusammen und besprechen die Planung. Wichtig zu erfahren ist, was die Ziele sind. Meine Aufgabe als Trainer ist es auch die ganzen Inputs von den Trainern zusammenzubringen und zu schauen, wie die Auslastung des Athleten ist und wo etwas verbessert werden kann. Der Fokus ergibt sich dementsprechend dann aus den Gesprächen mit den Trainern.

Ich: Welche spezifischen Indikatoren und Kennzahlen werden verwendet, um die Trainingspläne zu erstellen?

IP 1: Wir fragen ab, wie viele Wettkämpfe der Athlet geplant hat in diesem Jahr zu machen. Innerhalb des Trainings ist für mich der RPE wichtig, d.h. wie anstrengend das Training für den Athleten ist.

Ich: Werden vorab sportmotorische Tests durchgeführt?

IP 1: Grundsätzlich nicht, wir sagen nicht, dass bevor der Athlet bei uns trainieren möchte, muss er erst sportmotorische Tests durchführen. Sondern wir müssen sehen, dass sie sporttauglich sind. Das läuft über einen Arzt, der bestätigt, dass sie körperlich fit sind. Regelmäßig werden dann an der Institution Schwellendiagnostiken durchgeführt, wie z.B. Spiroergometrie, Laktattests.

Ich: Welche digitalen Hilfsmittel werden in der Trainingsplanung verwendet?

IP 1: Ich verwende primär Excel. Zusätzlich verwende ich Videoanalysetools wie Quick. Velocity pace Training, das die Hantelgeschwindigkeit misst. Ich verwende zusätzlich eine Datenbank, um die Daten zu sammeln und aufzuzeichnen. Pulsuhren oder Ringe verwenden wir auch, um Daten zu tracken.

Ich: Wie flexibel ist die Anpassung von Trainingsplänen innerhalb eines bestimmten Zeitraums (Vorbereitung, Wettkampfsaison)?

IP 1: Das ist unterschiedlich von Trainer zu Trainer. Ich mache es so, dass ich eine Woche Training plane. Dann warte ich auf das Feedback des Athleten und anhand dieses Feedbacks plane ich die nächste Trainingswoche. Wenn zum Beispiel die Übung XY mal viel schwerer war, passe ich das Gewicht sofort für die nächste Woche an. Wenn Tests unter der Zeit einer Trainingsphase gemacht werden, werden Kleinigkeiten im Training angepasst. Wenn der Test am Ende einer Trainingsphase stattfindet, dann ist das Ergebnis sehr maßgeblich für die nächste Trainingsphase.

Ich: Welche Herausforderungen/Komplikationen bei der Erstellung von Trainingsplänen können auftreten?

IP 1: Die größte Herausforderung ist das Belastungsmanagement, da die Athleten jeden Tag zwei oder mehr Trainingseinheiten haben und der Input nicht nur von einem Trainer kommt, sondern von mehreren. Das ist die größte Herausforderung aber gleichzeitig auch meine Aufgabe, um zu schauen was braucht der Athlet und was nicht.

Ich: Wie können die Herausforderungen bewältigt werden bzw. was wären digitale Lösungsmöglichkeiten?

IP 1: Wichtig ist die Kommunikation, um herausfinden zu können, wie es dem Athleten geht. Wellnessscore oder Stressindex, bei dem abgefragt wird, wie gestresst der Athlet ist. Zudem ist wichtig, dass man den Athleten gut einschätzen kann und weiß, wann der Athlet einem etwas vormacht. Eine digitale Lösungsmöglichkeit ist eben das Zusammenführen von den erhobenen Daten, die einen Einblick in den Zustand des Athleten geben. Es wird gerade SmarterBase entwickelt, was dazu beitragen soll, dass man alle Daten zusammenführen kann und diese auf einen Blick zur Hand hat.

Ich: Wie ist Ihre Einstellung künstlicher Intelligenz gegenüber? (pro/contra)

IP 1: Ziemlich neutral.

Ich: Welche Vorerfahrungen bestehen in Bezug auf künstliche Intelligenz im Sport?

IP 1: Ich selber habe keinerlei Vorerfahrungen in Bezug auf die künstliche Intelligenz.

Ich: Besteht grundsätzlich Vertrauen künstlicher Intelligenz gegenüber?

IP 1: Wenn es darum geht, nur Daten auszuwerten, dann hätte ich Vertrauen der KI gegenüber. Das Fehlerpotenzial, denke ich, ist bei der KI in Bezug auf Datenauswertung geringer als bei einem Menschen. Bei anderen Dingen möchte man vielleicht auch die Freiheit haben, von der KI abzuweichen.

Ich: Welche Relevanz hat künstliche Intelligenz bisher im Training des Leistungssports?

IP 1: Ich denke notwendig ist es nicht zwangsläufig, aber es kann eine Erleichterung darstellen. Es kann schon sein, dass die KI dann dazu führt, dass die Leistung des Athleten dann deshalb ein Stück besser ist.

Ich: Welche Anwendung findet künstliche Intelligenz (in welcher Form auch immer) aktuell?

IP 1: Aktuell findet künstliche Intelligenz keine Anwendung bei uns.

Ich: In welchem Bereich sehen Sie den größten Nutzen von künstlicher Intelligenz im Training des Leistungssports?

IP 1: Ich könnte mir vorstellen, dass künstliche Intelligenz einen Mehrwert bieten kann, wenn es darum geht, Daten auszuwerten und zu analysieren. Vor allem kann das Sinn machen, wenn es riesige Datenmengen sind. Künstliche Intelligenz kann da sicher eine große Zeitersparnis bewirken.

Ich: Wie wird die Entwicklung von künstlicher Intelligenz in Zukunft eingeschätzt?

IP 1: Ich glaube, dass dieses Thema immer größer wird. Vor allem in Hinblick auf die großen Datenmengen, welche ausgewertet und gefiltert werden müssen.

Ich: Künstliche Intelligenz eher als Hilfsmittel oder Ersatz für Trainer*in?

IP 1: Ersatz auf keinen Fall, aber als Hilfsmittel ist es mit Sicherheit gut geeignet. Ich denke, dass aktuell ein Trainer nicht von Technik ersetzt werden kann. Kann gut sein, dass das in zehn oder zwanzig Jahren möglich ist, aber momentan ist das nicht schaffbar, auch in Bezug auf die mentale Ebene. Man muss als Trainer auf die Emotionen des Athleten eingehen können. Die zwischenmenschliche Ebene ist im Leistungssport etwas sehr Wichtiges, auch von der psychologischen Seite her.

Ich: Besteht die Sorge, in Zukunft von künstlicher Intelligenz als Trainer*in ersetzt zu werden?

IP 1: Nein, momentan besteht die Sorge nicht. Es gibt Dinge, die nicht nur objektiv eingeschätzt werden dürfen, sondern auch subjektiv durch einen Menschen. Diese Dinge müssen jedes Mal aufs Neue wieder interpretiert werden.

Ich: Wie ist Ihre Einschätzung zu diesen beiden Trainingsplänen?

IP 1: Grundsätzlich hat Trainingsplan 1 eine Periodisierung, also der Plan ist nach jedem Wochentag aufgeteilt. Es sind in beiden Blöcken jeweils vier Trainingstage, die in Grundlagentraining, Intervalltraining und mittlere Belastung aufgeteilt sind. Der Plan ist sehr allgemein gehalten, aber funktioniert sicherlich, um den Halbmarathon laufen zu können.

Trainingsplan 2 beinhaltet Dehnen vor jedem Training zum Aufwärmen. Ich denke, das hat keinen Einfluss auf die Leistung beim Halbmarathon dann, das Dehnen braucht es nicht unbedingt. Trainingsplan hat Informationen über die Strecke beim Training, wobei 2 über die Zeit gesteuert wird. Ich denke grundsätzlich funktionieren beide Pläne, die Darstellung ist nur etwas anders. Trainingsplan 2 ist etwas genauer dargestellt, Plan 1 ist allgemeiner gehalten.

Ich: Welcher der beiden Pläne wurde von einer künstlichen Intelligenz erstellt?

IP 1: Rein von der Informationsdichte, die der Athlet in Trainingsplan 2 bekommt, lässt mich darauf schließen, dass dieser Plan von einem Trainer erstellt wurde.

Ich: Vielen Dank für deine Zeit!

IP 1: Gerne, kein Problem.

Interviewpartner 2, 13.12.2023

Ich: Wie lange bist du bereits als Trainer im Leistungssport tätig?

IP 2: Ich bin jetzt im Olympiazentrum seit fast zwei Jahren, war aber davor schon im alpinen Skirennlauf im Nachwuchsleistungssport tätig für zwei Jahre. Also insgesamt 4 Jahre als Trainer im Leistungssport tätig und vorher war ich selber aktiver Leistungssportler.

Ich: Und der alpine Skirennlauf ist dann auch dein Spezialgebiet? Betreust du also nur Skirennläufer?

IP 2: Nein, also ich betreue so wie alle anderen Trainer nicht nur eine Sportart. Es gibt jetzt nicht eine Spezialsportart. Natürlich, als ehemaliger Skirennläufer hat man tendenziell mehr Athleten aus dem Skirennlauf, aber es sind ansonsten Sportarten durch die Bank dabei. Also ich bin wirklich allgemeiner Athletiktrainer und Leistungsdiagnostiker.

Ich: Welche Qualifikationen haben Sie bereits in Ihrer Laufbahn erlangt?

IP 2: Also ich habe das Bachelorstudium Sportwissenschaften hier in Innsbruck gemacht und werde im Januar oder Februar meine Masterarbeit abgeben. Also noch nicht ganz fertiger Master Sportwissenschaft. Ansonsten habe ich Zusatzausbildungen wie den Trainer Grundkurs bei der BSPA gemacht. Spezialtrainerkurse auch, wobei die für meine aktuelle Tätigkeit weniger relevant sind.

Ich: In Bezug auf die Trainingsplanung, was ist dein Spezialgebiet?

IP 2: Momentan eher die Rehabilitationsrahmenprogramme. Ansonsten, dadurch, dass ich selber aus dem Ski Alpin komme, Trainingsplanung Ski Alpin. Aber ich betreue eben Sportarten wie Voltigieren, Breakdance, Tennis. Die letzten Monate lag der Schwerpunkt auf Knieverletzungsrehabilitation. Dadurch, dass ich einen verletzten Sportler dazubekommen habe, habe ich vermehrten Einblick in die Rehaplanung bekommen.

Ich: Wie ist grundsätzlich der Ablauf von der Planung bis hinzu den fertig erstellten Trainingsplänen? In Bezug auf die Informationsbeschaffung, Fokussetzung, Leistungsmerkmale.

IP 2: Einerseits ist es wichtig, welche Sportart man betreut, also die Sportartanalyse ist der erste Schritt, um einen Plan erstellen zu können. Es ist wichtig zu wissen, welche Voraussetzungen die Sportart und der Athlet hat. Diese werden durch Eingangstests herausgefunden. Dann schau ich mir den Jahresplan an in Bezug auf die Wettkämpfe des Athleten. Die Trainingsplanung soll eben auf Wettkämpfe hinzielen. Es ist wichtig zu wissen, wie der Zyklus ist, ob es eine Sommer- oder Wintersportart ist. Und dann muss man den Athleten selber einstufen, welche Ziele hat der Athlet, welche Bausteine man legen sollte, damit das funktioniert und der Athlet dort hinkommt, wo er hin will. Das eigene Ziel des Athleten sollte dabei sehr viel Gewicht haben, man versucht immer einen Konsens zu finden. Dadurch, dass wir im Olympiazentrum sehr interdisziplinär arbeiten, arbeiten wir unter den Trainern für einen Athleten auch zusammen. Grundsätzlich ist es so, dass hier im Olympiazentrum das athletische, psychologische bis hin zur Ernährungsberatung alles abgewickelt wird. Ein Baustein für die Fokussetzung in der Trainingsplanung ist eben durch leistungsdiagnostische Tests gegeben.

Ich: Werden vorab sportmotorische Tests durchgeführt?

IP 2: Sportmotorische Tests werden einige durchgeführt. Wir machen einige Krafttestungen, Schnelligkeitstestungen. Gatter Test, Speedy Jump Test für die Schnelligkeit, Kraftmessplatte, Sprungkrafttestungen Counter Movement Jumps, Drop Jumps, Tapping Tests sowohl Arme als auch Beine, Krafttests mit isometric leg drunk, 1RM Tests, isokinetische Tests Contrex. Ansonsten nutzen wir im Trainingssetting einige Tests mit Gymaware, Trainingstool für die force velocity.

Ich: Gibt es außer den genannten Tools digitale Hilfsmittel, die du zur Trainingsplanung verwendest?

IP: Durchaus ja, ich arbeite mit Trainingpeaks. Das ist ein Tool für die Trainingsplanung. Die Trainingseinheiten erstelle ich klassisch über Numbers oder Excel. Smarterbase nutzen wir ansonsten, das ist vorrangig ein Wellnessmonitoring. Der Athlet füllt da jeden Morgen aus, wie er sich fühlt, wie viel und wie gut er geschlafen hat, wie ist der muskuläre Zustand, also der allgemeine Zustand.

Ich: Wie flexibel ist die Anpassung von Trainingsplänen innerhalb eines bestimmten Zeitraums (Vorbereitung, Wettkampfsaison)?

IP 2: Vorbereitung: Es gibt einen Plan, wobei der Plan dann selten so aufgeht, wie man den macht, weil natürlich muss man das subjektive Empfinden des Athleten mit einbeziehen. Man muss auf unvorhersehbare Dinge, wie z.B. Muskelkater, reagieren. Da ist natürlich kurzfristige Flexibilität gefragt. Die langfristige Planung wird aber schon so durchgezogen, ob es jetzt genau die 3 Wochen und dann eine Woche Pause ist oder 4 Wochen und eine Woche Pause, wird dann meistens kurzfristig angepasst. In der Wettkampfsaison kommt es auf die Sportart an, je nach Terminkalender, je nach Verschiebungen muss man schon relativ flexibel sein. Die Inhalte innerhalb der Trainingswoche können laufend angepasst werden.

Ich: Welche Herausforderungen/Komplikationen bei der Erstellung von Trainingsplänen können auftreten?

IP 2: Einerseits der Athlet selbst, was wünscht er sich, wie viel Engagement zeigt er, wie viel Zeit hat er überhaupt. Es gibt viele Sportler, die studieren nebenher, die haben kleinere Berufe und somit auch andere Verpflichtungen, denen sie nachkommen müssen. Der zweite Punkt ist, wie viel sie wirklich Spezialsportart trainieren können. Wenn z.B. ein Tennisspieler jeden Tag die Möglichkeit hat, in die Halle zum Trainieren zu gehen, ist die Herausforderung groß, athletische Komponenten damit zu vereinbaren. Da spielt die Regeneration eine große Rolle, wo wir als Coaches schauen müssen, dass die Auslastung des Athleten passt. Wir versuchen immer das Maximum aus dem Athleten herauszuholen, wobei wir auf den Fokus auf die langfristige Entwicklung des Athleten legen und das überschneidet sich oft ein wenig.

Ich: Wie können die Herausforderungen bewältigt werden bzw. was wären digitale Lösungsmöglichkeiten?

IP 2: Es ist extrem wichtig, eine gute Beziehung zum Sportler aufzubauen. Er muss dir alles sagen können, gleichzeitig musst du ihm auch alles sagen können, ohne dass er beleidigt ist. Zusätzlich dient das angesprochene Tracking dazu, die Herausforderung zu bewältigen. Zudem ist der ständige Austausch mit dem Sportler sehr wichtig.

Ich: Wie ist Ihre Einstellung künstlicher Intelligenz gegenüber? (pro/contra)

IP 2: Es kann auf alle Fälle eine Hilfe sein. Die menschliche Ebene fehlt aus meiner Sicht. Persönliche Aspekte gehen dabei verloren, jedoch denke ich, dass es eine Hilfestellung in jeglichen Situationen bieten kann. Es kann also eine gute Ergänzung sein. Man darf nie die eigene Arbeit dabei vergessen. Gerade in Bezug auf Datenanalyse kann es eine gute Hilfe sein. Aber aktuell denke ich, dass die künstliche Intelligenz nicht so weit vorangeschritten ist, dass man sich zu 100% darauf verlassen kann.

Ich: Welche Vorerfahrungen bestehen in Bezug auf künstliche Intelligenz im Sport?

IP 2: Bisher nur ChatGPT. Beim Ausprobieren der Funktionen stellt man fest, dass man die Informationen von ChatGPT hinterfragen muss. In Bezug auf unser Training habe ich keine Vorerfahrungen was die künstliche Intelligenz angeht.

Ich: Besteht grundsätzlich Vertrauen künstlicher Intelligenz gegenüber?

IP 2: Zu 100% nicht, eher dann als Hilfsmittel.

Ich: Welche Relevanz hat künstliche Intelligenz bisher im Training des Leistungssports?

IP 2: Bisher hat künstliche Intelligenz keine Relevanz bei uns im Training.

Ich: Welche Anwendung findet künstliche Intelligenz (in welcher Form auch immer) aktuell?

IP 2: Bisher findet künstliche Intelligenz keine Anwendung bei uns.

Ich: In welchem Bereich sehen Sie den größten Nutzen von künstlicher Intelligenz im Training des Leistungssports?

IP 2: Zur Verarbeitung von großen Datenmengen. Zur Datenanalyse.

Ich: Wie wird die Entwicklung von künstlicher Intelligenz in Zukunft eingeschätzt?

IP 2: Im Spitzensportsetting wird es immer wichtig bleiben, dass eine Beziehungsebene auf menschlicher Ebene stattfindet. Die kurzfristige Anpassung ist wichtig, um das optimale Training für den Athleten bieten zu können.

Ich: Künstliche Intelligenz eher als Hilfsmittel oder Ersatz für Trainer*in?

IP2: Im Spitzensport denke ich nicht, dass Trainer von einer künstlichen Intelligenz ersetzt werden können, da es die menschliche Komponente immer brauchen wird. Es braucht

außerdem einen Menschen, der die Informationen der künstlichen Intelligenz kritisch beurteilen kann.

Ich: Besteht die Sorge, in Zukunft von künstlicher Intelligenz als Trainer*in ersetzt zu werden?

IP 2: Nein, es besteht keine Sorge.

Ich: Wie ist Ihre Einschätzung zu diesen beiden Trainingsplänen?

IP 2: Trainingsplan 2 hat sehr viel Information, eher schon zu viel Information. Trainingsplan 1 hat die Informationen auf den Punkt gebracht. Bei Trainingsplan 1 versteh ich alles. Vom Aufbau her macht Trainingsplan 1 für mich mehr Sinn. Trainingsplan 2 hat eine sehr hohe Intensität. Vom Aufbau und vom Verständnis her finde ich den Trainingsplan 1 besser. Grundsätzlich funktionieren beide Trainingspläne zur Vorbereitung von einem Halbmarathon, jedoch finde ich, dass Trainingsplan 1 verständlicher für den Athleten ist, vor allem vor dem Hintergrund, dass der Athlet zum ersten Mal einen Halbmarathon läuft.

Ich: Vielen Dank für deine Zeit.

IP 2: Gerne!

Interviewpartner 3, 15.12.2023

Ich: Wie lange sind Sie bereits als Trainer*in im Leistungssport aktiv?
IP 3: Ich bin jetzt seit 01.01.2020 hier am Olympiazentrum tätig, habe also somit fast die vier Jahre voll. Davor habe ich aber bereits 20 Jahre Berufserfahrung in leitender Position als Cheftrainer im Skispringen gesammelt.
Ich: Wie lange arbeiten Sie bereits in Ihrer Institution?
IP 3: Seit knapp vier Jahren bin ich nun hier am Olympiazentrum tätig.
Ich: Welche Qualifikationen haben Sie bereits in Ihrer Laufbahn erlangt?
IP 3: Angefangen habe ich beim ÖSV Skispringen als Trainer der Damen. Das habe ich zwei Jahre gemacht. Danach bin ich nach Italien zum italienischen Skiverband und habe dort Damenskispringen von null auf entwickelt. Wir haben dort mit Teenagern begonnen und ihnen Sprungski gezeigt und über Jahre betreut bis hinzu der Weltmeisterschaft in Oslo 2011. Danach habe ich noch zwei Jahre die nordische Kombination in Italien geleitet. Dann war ich ein Jahr in der Schweiz als Cheftrainer Damen Skisprung. Im Anschluss daran habe ich im Nachwuchs im Skiclub Garmisch-Patenkirchen gearbeitet. Früher war ich selber Skispringer im Leistungssport und habe dementsprechend eine Sportschule besucht. War eben auch im Kader der österreichischen Nationalmannschaft und war selber kurz im Weltcup aktiv. Ich habe zudem Sportmanagement studiert. Ich habe Trainerausbildungen gemacht, Lehrwart, Allgemeintrainer, Spezialtrainer im Skisprung, Lehrwart in der nordischen Kombination. Die letzten Jahre habe ich einige Ausbildungen im Bereich Neuroathletik gemacht.
Ich: Was ist Ihr Spezialgebiet/was liegt Ihnen am meisten in Bezug auf die Trainingsplanung?
IP 3: Als Athletik- bzw. Strength und Conditioning Trainer sind wir für verschiedene Sportarten zuständig. Jeder Trainer hat mehrere Athleten, die verschiedene Sportarten ausüben. Mein Großteil der Athleten kommt aus dem Klettern, nämlich vier Speedkletterathleten, einen männlichen Boulderathleten und eine weibliche Boulderathletin (Combained), eine Paraathletin, einen Kunstturner, eine Taekwandokämpferin, einen Skispringer, den ich im Neuroathletiktraining betreue. Grundsätzlich bei uns im Haus ist es so, dass wir aufgrund von organisatorischen Gründen eine Mischung an Winter- und Sommersportarten betreuen. In Abstimmung mit dem zusätzlichen Spezialtrainer betreue ich dann den jeweiligen Athleten. Wir bekommen dabei spezielle Vorgaben von den Spezialtrainern, die wir einhalten sollten. Trotzdem haben wir Freiheiten, die wir in die Trainingsplanung miteinfließen lassen können. Allgemein betreue ich Sportarten, bei denen Schnellkraft sehr wichtig ist, deshalb ist das meine Kernkompetenz. Unsere Institution steht dafür, dass wir gut als Team funktionieren und die Spezialgebiete in der Trainingsplanung auf die passenden Trainer aufteilt werden.
Ich: Wie ist der Ablauf von der Planung bis hinzu den fertig erstellten Trainingsplänen?
IP 3: Ganz simpel gesagt, gibt es einen Wettkampfkalender für den jeweiligen Athleten. Dann werden die Wettkämpfe durch die Spezialtrainer oder den Wunsch des Athleten priorisiert. Danach wird überlegt, wie man diese Anforderungen durch das Training erreichen kann. Wir versuchen dies Leistungsdiagnostikbasiert zu machen, d.h. man führt Gespräche mit den Spezialtrainern und macht das Stärken-Schwächen-Profil des Athleten aus.
Ich: Welche spezifischen Indikatoren und Kennzahlen werden verwendet, um die Trainingspläne zu erstellen?
IP 3: Wir verwenden hauptsächlich relative Werte, da diese aussagekräftiger sind als absolute Werte. Zudem versuchen wir Werte in der Gruppe zu vergleichen, damit man Referenzwerte hat. Der Vorteil vom Olympiazentrum ist, dass es sehr viel Leistungsdiagnostik gibt und dies auch schon über viele Jahre und viele Sportarten hinweg. Das heißt, man weiß, dass die Skifahrer z.B. in der 1RM Kniebeuge und die Skispringer bei den Sprungkrafttests das Maß aller Dinge sind. Man schaut sich dann an, wie beispielsweise der Kletterer bei diesen Tests abschneidet. Bei solchen neuen Sportarten ist eben die Frage, was denn der Sollwert sein sollte, da es noch nicht so viele Erfahrungen in Bezug auf die Daten gibt. Dies ist eben dann unsere Aufgabe, das zu erforschen.
Ich: Werden vorab sportmotorische Tests durchgeführt?

IP 3: Wenn nicht im Vorhinein bereits von den Verbänden Tests durchgeführt wurden, haben wir hier natürlich auch die Möglichkeit, Tests mit den Athleten zu machen. Wenn wir selber an der Institution testen, dann testen wir nicht so umfangreich, sondern nur das, was wir auch als nächsten Fokus haben. Wir machen z.B. Sprungkrafttests, Maximalkrafttests, Tappingtests, Spiroergometrien, Ergometrien, Rumpfkrafttests, Schulterrotationstests, Bankziehen, Bankdrücken. Wir schauen immer sehr viel auf die Gesamtgesundheit des Athleten, d.h. wir können uns die Zeit nehmen, um muskuläre Dysbalancen auszugleichen. Wenn durch die Leistungsdiagnostischen Tests beispielsweise auch durch Verletzungen Defizite auftauchen, wird sich die Zeit genommen, um darauf lang genug eingehen zu können. Trotzdem geht es natürlich darum, dass der Athlet maximale Leistung bringt, nur unter der Bedingung, dass es dem Athleten auch gut geht.

Ich: Welche digitalen Hilfsmittel werden in der Trainingsplanung verwendet?

IP 3: Immer mehr werden natürlich Wearables verwendet, um verschiedene Dinge zu tracken. Herzfrequenzmessung, Pulsgurt, Aora-Ring, Wooparmband.

Ich: Wie flexibel ist die Anpassung von Trainingsplänen innerhalb eines bestimmten Zeitraums (Vorbereitung, Wettkampfsaison)?

IP 3: Grundsätzlich ist es natürlich nicht sehr erwünscht, als Trainer die Pläne anzupassen, jedoch ist dies „daily business". Es muss also flexibel sein, aber wenn etwas dazwischen kommt wie Krankheit oder Verletzung, dann muss man reagieren. Auf Basis von Wearable-Daten den Trainingsplan anpassen, sehe ich eher skeptisch an, da bislang nicht diese Langzeiterfahrungen der Wearables vorhanden sind und diese Dinge eher als Zusatzinformation gesehen werden sollten. Eine Woche sollte man den Trainingsplan schon mal durchgezogen haben, um zu sehen, ob es gut funktioniert. Ansonsten ist es eher ein Bauchgefühl, das man hat.

Ich: Welche Herausforderungen/Komplikationen bei der Erstellung von Trainingsplänen können auftreten?

IP 3: Die Schwierigkeit ist, dass es nicht wirklich einen Athleten gibt, bei dem man nur mit der sportlichen Notwendigkeit planen kann. Also in Österreich haben wir noch keine Kultur, bei dem der Profisportler alles dem Sport unterordnet, sondern haben auch noch andere Verpflichtungen nebenher, denen sie nachkommen müssen. Auf diese Dinge müssen wir als Trainer natürlich Rücksicht nehmen. Für die Wochenplanung heißt das, dass im Vorhinein abgeklärt werden muss, wann der Athlet wie viel Zeit hat für das Training. Als Student zum Beispiel muss der Athlet vorab einschätzen können, wann er mehr Zeit für die Uni braucht und wann nicht.

Ich: Wie können die Herausforderungen bewältigt werden bzw. was wären digitale Lösungsmöglichkeiten?

IP 3: Sehr wichtig ist ein gutes Zeitmanagement zu pflegen. Wie gesagt ist es im Vorhinein einer Trainingsphase wichtig, dass man klar über die verfügbare Zeit des Athleten für das Training kommuniziert. Es gibt kein Programm, dass automatisiert das Zeitmanagement erfasst. Was auf jeden Fall die Kommunikation erleichtert, ist, dass ich mittlerweile sehr viel digitale Trainingspläne über eine Software, die Appgesteuert läuft, erstelle. Der Athlet hat somit alles selber bei sich am Handy im Überblick, damit er auch mal remote trainieren kann, falls etwas dazwischen kommt. Ich als Trainer kann somit eben trotzdem feststellen, welches Training absolviert wurde und wie das Training durchgeführt wurde. Das Programm dafür heißt AthleteMonitoring. Es wird in Zukunft in Österreich wahrscheinlich eine große Änderung noch geben. Es gibt aktuell ein großes Projekt vom Österreichischen Olympischen Komitee, um all diese riesen Datenmengen, die über jeden Athleten bestehen, besser verarbeiten zu können. Diese sollen auf einer Plattform digital automatisiert erhoben werden, damit alle Informationen aus der Leistungsdiagnostik von verschiedenen Tools an einem Speicherort zusammenkommen. Die Software heißt SmarterBase und kommt ursprünglich aus Australien und es geht hauptsächlich um die Steuerung und die Verfügbarkeiten von Athleten. Wann können sie belastbare Trainings machen und wann nicht. Das Ziel dabei ist, Ausfälle aufgrund von Verletzungen zu reduzieren. Aktuell ist dies noch sehr viel Handarbeit, wobei der Ausdauerbereich sehr viel weiter fortgeschritten ist. Trainingpeaks ist da ein gutes Beispiel, das als Goldstandard in der Ausdauertrainingsplanung und -Trainingsaufzeichnung benannt werden kann.

Ich: Wie ist Ihre Einstellung künstlicher Intelligenz gegenüber? (pro/contra)

IP 3: Da ich hier im Haus einer der älteren Trainer bin, bin ich dementsprechend am weitesten weg von dem Thema. Ich finde das Thema extrem spannend. Für sehr viel standardisierte Arbeit glaube ich, dass künstliche Intelligenz sehr gut funktionieren kann. Aber bei uns im Spitzensport ist das Training nun mal nicht standardisiert. Ich habe zum Beispiel vier Athleten, die eins zu eins alles aus meiner Hand bekommen, aber sie haben nicht den gleichen Trainingsplan. Also selbst unter diesen vier, die alle die gleichen Wettkämpfe machen und die gleiche Priorisierung haben, ist es nicht „copy, paste". Wenn ich den Aufwand sehe, was man alles mit einfließen lassen muss, stellt sich für mich die Frage, ob das wirklich irgendwann von einer künstlichen Intelligenz abgenommen werden kann. Dafür muss man sehr sauber die künstliche Intelligenz mit Daten füttern, dass ein sinnvoller Output dabei rauskommt. Digitale Medien konstant über langen Zeitraum sinnvoll gefüllt zu behalten ist nach wie vor das Schwierigste daran.

Ich: Welche Vorerfahrungen bestehen in Bezug auf künstliche Intelligenz im Sport?

IP 3: Nein, da bin ich wirklich sehr weit davon entfernt. Wobei einen kurzen Kontakt mit künstlicher Intelligenz hatten wir bereits. Dabei haben wir eine Videosoftware, die Körpergelenkpunkte erkennt und dann sieht, wie der Körperschwerpunktverlauf war und dementsprechend dann die Geschwindigkeit berechnen kann. Diese Software lernt mit der Zeit immer mehr dazu und kann sagen, wenn ein Körperteil verdeckt ist, wo es sich gerade befinden muss. Jedoch ist das Ergebnis bisher nicht wirklich genau genug, sodass man nicht sagen kann, ich bin mit manueller Arbeit langsamer. Ich brauche natürlich sehr viel mehr Zeit, dies händisch auszuwerten. Jedoch ist es ein trade off, den man machen muss. Das ungenaue Ergebnis kann dazu führen, dass ich es dann sowieso nicht richtig für Rückschlüsse verwenden kann. Ich glaube aktuell will jeder künstliche Intelligenz verwenden, jedoch muss man sie zuerst einmal mit hochfaktenbasierten Informationen füttern, um die Ergebnisse dann nutzen zu können. Ich glaube, dass man momentan den zweiten Schritt vor dem ersten macht.

Ich: Besteht grundsätzlich Vertrauen künstlicher Intelligenz gegenüber?

IP 3: Ich glaube - ganz arrogant gesagt - je banaler die Aufgabe für die KI ist, desto höher ist mein Vertrauen der künstlichen Intelligenz gegenüber.

Ich: Welche Relevanz hat künstliche Intelligenz bisher im Training des Leistungssports?

IP 3: Nein, also wir verwenden zwar viele standardisierte Berechnungsmethoden im Hintergrund, aber eher in Richtung Statistik.

Ich: Welche Anwendung findet künstliche Intelligenz (in welcher Form auch immer) aktuell?

IP 3: Bis auf diese Software, die ich vorhin genannt habe, findet künstliche Intelligenz aktuell keine Anwendung.

Ich: In welchem Bereich sehen Sie den größten Nutzen von künstlicher Intelligenz im Training des Leistungssports?

IP 3: Die Gefahr, wenn ein Mensch etwas macht, ist immer das Bauchgefühl, was dem Menschen dazwischen kommen kann, dass er nicht faktenbasiert reagiert oder handelt. Dies ist zwar auf der einen Seite eine potenzielle Gefahr, jedoch auch die größte Chance des Menschen ist, dass er immer über der künstlichen Intelligenz stehen wird. Der Mensch wird immer emphatischer sein als die künstliche Intelligenz. Ich glaube, es ist sehr cool, wenn man sein eigenes Gefühl als Trainer faktenbasiert untermauert bekommen würde, jedoch könnte auch der Konflikt aufkommen, dass die KI etwas anderes sagt als der Trainer eigentlich vorhatte.

Ich: Wie wird die Entwicklung von künstlicher Intelligenz in Zukunft eingeschätzt?

IP 3: Ich glaube, dass künstliche Intelligenz im Training im Breitensport irgendwann mal sehr gut funktionieren könnte, da die Informationen der KI für die breite Masse der Bevölkerung sehr gut funktionieren. Ich glaube, dass sich dieses Thema allgemein in Zukunft sehr verändern wird und wir mit der Zeit lernen werden, dort viel mehr Vertrauen zu haben, weil man mit der Zeit auch viel versucht hat und Erfahrungen gemacht hat. Ähnlich war es damals als Herzfrequenzmessungen sehr neu waren. Heute stellt einen Pulsgurt wohl niemand mehr in Frage. Ich kann mir vorstellen, dass das mit KI ähnlich ablaufen wird. Am Anfang ist man noch sehr skeptisch und hinterfragt und wenn man dann ausreichend

Erfahrung gesammelt hat, kann mehr Vertrauen aufgebaut werden. Ich denke trotzdem, dass das lange Prozesse sein werden, bis dieser Punkt erreicht wird.

Ich: Künstliche Intelligenz eher als Hilfsmittel oder Ersatz für Trainer*in?

IP 3: Wie gesagt, ich glaube im Breitensport kann ein Trainer von der künstlichen Intelligenz ersetzt werden. Der Hobbyläufer kann sicher auch ohne Trainer gut zurechtkommen, wenn er seine Daten der KI gibt. In der oberen Spitze im Spitzensport glaube ich, dass dieser Ersatz nicht möglich sein wird, da diese individuelle Betreuung durch Zwischenmenschlichkeit nochmal mehr gebraucht wird. Die Belastungen, die dort auf einem Athleten lasten, auch außerhalb der reinen Trainingsbelastung, dass da dieser Austausch und das Vertrauen gegenüber dem Trainer zu wichtig ist.

Ich: Besteht die Sorge, in Zukunft von künstlicher Intelligenz als Trainer*in ersetzt zu werden?

IP 3: Ich muss nur noch zwanzig Jahre arbeiten, ich komme also sicher noch durch. Für einen aktuellen Absolventen eines Sportstudiums würde ich eventuell eine andere Prognose machen. Nein, also ich habe keine Sorge ersetzt zu werden. Unser Altersunterschied spielt dort aber eine große Rolle, denn ich glaube schon, dass unser Job in der großen Masse eben durch solche Technologien ersetzt wird. Und gerade wenn man keinen Spitzensporthintergrund hat, kommt man schwer in dieses Spitzensportsetting hinein.

Ich: Wie ist Ihre Einschätzung zu diesen beiden Trainingsplänen?

IP 3: Ohne dass ich mir die Trainingspläne genau durchlese, kann ich vorab sagen, dass beide nicht ansehnlich und nicht strukturiert sind. Die Workoutbeschreibung sollte nicht in einem Zweizeiler geschrieben sein, jedes Mal muss ich nach der Distanz und Intensität suchen. Die Tabelle sollte also übersichtlich und einheitlich aufgebaut sein. Bei Plan 2 muss der Athlet sich selber schon gut kennen, dass er genau weiß, was zu tun ist. Ohne Vorerfahrung stelle ich mir das schwierig vor. Es muss für den Empfänger auf dem jeweiligen Niveau eindeutig sein, was zu machen ist. Es sind sehr viele Variablen angegeben, wobei es für mich als Trainer schwierig nachzuvollziehen ist, was der Athlet dann wirklich gemacht hat. Trainingsplan 1 ist bisschen kompakter gehalten. Es ist konkreter angegeben, was der Athlet zu tun hat. Vor dem Hintergrund, dass beide Trainingspläne für jemanden erstellt wurden, der zum ersten Mal einen Halbmarathon laufen möchte, würde ich den Plan 1 präferieren, weil allein schon weniger Text ist und die Hürde dann kleiner ist. Der Plan ist wie ein Kuchenrezept, er gibt die Dauer, Intensität und Trainingsmittel an und dann kann es losgehen. Plan 2 hat zu viele Entscheidungsvarianten.

Ich: Vielen Dank für deine Zeit!

IP 3: Gerne, kein Problem.

Interviewpartner 4, 19.12.2023

Ich: Wie lange sind Sie bereits als Trainer*in im Leistungssport aktiv?

IP 4: Am Olympiazentrum generell war ich nie als aktiver Trainer tätig. Ich habe das Olympiazentrum gegründet und damals 2011 sofort die Leitung übernommen. 2001 bin ich von Salzburg wieder zurück nach Innsbruck gegangen und ab da haben wir das trainingswissenschaftliche Zentrum gegründet. Damals war meine Tätigkeit primär in der Leistungsdiagnostik, trotzdem hatte ich gleichzeitig das Interesse, Athletinnen und Athleten direkt zu betreuen. Wir haben hier am Olympiazentrum einen sehr intensiven Austausch innerhalb des Teams und ich habe auch die Verantwortung für die Tätigkeiten von meinen Mitarbeiterinnen und Mitarbeitern. Ich selber war aber nie aktiv verantwortlich als Trainer für einen einzelnen Athleten.

Ich: Wie lange arbeiten Sie bereits in Ihrer Institution?

IP 4: Seit 2011 arbeite ich als Leiter des Olympiazentrums.

Ich: Welche Qualifikationen haben Sie bereits in Ihrer Laufbahn erlangt?

IP 4: Ich habe hier in Innsbruck Sport studiert und bin danach nach Salzburg gegangen und habe dort mein Doktorat gemacht. Nebenher habe ich damals noch die Skitrainerausbildung gemacht. Zusätzlich dazu habe ich einige Fortbildungen im Bereich Trainingswissenschaften gemacht, nachdem ich zuerst mehr in der Biomechanik tätig war. Als ich von Salzburg zurückgekommen bin, habe ich in Innsbruck an der Sportuniversität als Dozent begonnen.

Ich: Was ist Ihr Spezialgebiet/was liegt Ihnen am meisten in Bezug auf die Trainingsplanung?

IP 4: Da ich aus dem Skirennlauf komme, habe ich auch immer hauptsächlich Forschung im Skirennlauf betrieben. Gleichzeitig war für mich die Vielfalt an Athleten in Bezug auf verschiedene Sportarten sehr wichtig. Wenn Bedarf war oder ich angefragt wurde, habe ich Athleten betreut und beraten. Sportarten wie Golf, Eiskunstlauf, Snowboard, Ski, Wasserski, Bob, Formel 1 habe ich betreut. Das war für mich immer eine tolle Herausforderung. Man muss sich eben in jede Sportart hineindenken, um dann die richtigen Trainingspläne erstellen zu können. In Bezug auf die Trainingsplanung ist der konditionelle Faktor der Hauptbereich.

Ich: Wie ist der Ablauf von der Planung bis hinzu den fertig erstellten Trainingsplänen?

IP 4: Das Wichtigste ist zuerst die Anforderungen der jeweiligen Sportart auszumachen und mit Experten, die aus der Sportart kommen, zu sprechen. Man muss verstehen, welche Anforderungen die Sportart technisch, taktisch, psychologisch und körperlich mit sich bringt. Es kommt darauf an, ob die Sportart eine Ganzjahressportart oder saisonale Sportart ist. Es ist zudem sehr wichtig, Personen aus der Praxis, die sehr lange schon in der Sportart tätig sind, zu Rate zu ziehen. Bevor man dann mit der Trainingsplanung beginnen kann, muss man wissen, wo der Athlet aktuell steht. Dafür macht man Analysen, wie die Saison verlaufen ist, in welchem Bereich gab es Erfolge und wo nicht. Wichtig zudem ist die Leistungsdiagnostik, je nachdem wo der Schwerpunkt gesetzt wurde. Aufbauend auf diesen Informationen und der Zielsetzung des Trainers und des Athleten, kann die Planung für die Saison begonnen werden.

Ich: Welche spezifischen Indikatoren und Kennzahlen werden verwendet, um die Trainingspläne zu erstellen?

IP 4: In Bezug auf die körperliche Vorbereitung des Athleten sollte auf wissenschaftlich evaluierte Tests, bei denen hoffentlich bereits Normwerte von den Athleten vorliegen, zurückgegriffen werden. Bei Nachwuchsathleten wäre es optimal, wenn für die einzelnen Jahrgänge oder Altersgruppen Normwerte vorliegen, damit man dann einen Vergleich schaffen und seine Stärken und Schwächen ausmachen kann. Aus diesen Erkenntnissen leitet sich dann auch die Schwerpunktsetzung für die Trainingsplanung ab.

Ich: Werden vorab sportmotorische Tests durchgeführt?

IP 4: Es ist sicherlich ein Schwerpunkt hier in Innsbruck schon immer gewesen, dass viele Tests durchgeführt werden. Mit Experten wurden dann sieben bis acht Tests ausgemacht, die am meisten Sinn machen. Dabei sind Testungen wie 1RM, Kniebeugen, isokinetische Tests, Rumpf, Beinkraft, Sprungkraft, Reaktivkraft, Kraftausdauer, agility test je nach Sportart maßgeblich. Die Tests unterscheiden sich also von Sportart zu Sportart.

Ich: Welche digitalen Hilfsmittel werden in der Trainingsplanung verwendet?

IP 4: Hilfsmittel sind im Grunde genommen alle Ergebnisse, die man aus den Tests zieht, da man einen Vergleich mit den Normwerten herstellen kann. Aktuell in der Leistungsdiagnostik werden vereinzelt digitale Hilfsmittel wie z.B. das Nearsystem verwendet. Dies wurde bei Radfahrern verwendet, um die Sitzposition zu analysieren. Ansonsten ist die Leistungsdiagnostik eher klassisch mit stationären Geräten. Gleichzeitig werden die Geräte immer kleiner, gerade wenn man beispielsweise auf Gymaware Bezug nimmt. Die Verwendung von Wearables ist dann der nächste Schritt, der dann nach der erfolgten Trainingsplanung als digitales Hilfsmittel eingesetzt wird, um das Training zu steuern oder zu überwachen.

Ich: Wie flexibel ist die Anpassung von Trainingsplänen innerhalb eines bestimmten Zeitraums (Vorbereitung, Wettkampfsaison)?

IP 4: Die Flexibilität in der Anpassung von Trainingsplänen ist etwas, was einen guten von einem nicht so guten Trainer unterscheidet. Dass man dieses Gespür entwickeln kann, benötigt natürlich auch Erfahrung. Wichtig zu wissen ist, dass der Trainer einschätzen kann, wann der Athlet eine höhere Auslastung braucht, um das Ziel zu erreichen und wann weniger manchmal mehr sein kann. Wichtig zudem ist das offene Vertrauensverhältnis zwischen den handelnden Personen, dass immer gut kommuniziert wird, wie es einem gerade geht. Zusätzlich dazu ist wichtig, dass man immer die aktuelle Auslastung durch Wettkämpfe für die nächste Trainingsplanung mitberücksichtigt. Der Aspekt der Regeneration spielt dabei eine sehr wichtige Rolle.

Ich: Welche Herausforderungen/Komplikationen bei der Erstellung von Trainingsplänen können auftreten?

IP 4: Die Anpassung der richtigen Auslastung zu jedem Zeitpunkt ist definitiv eine Schwierigkeit, die beim Erstellen der Trainingspläne aufkommt. Der Trainer muss kurzfristig nach einem Wettkampf einschätzen können, wie stark die Auslastung durch den Wettkampf war und welche regenerativen Prozesse es benötigt, um den Athleten wieder voll leistungsfähig zu machen. Unvorhersehbare Dinge sind zudem Schwierigkeiten, die auftreten können. Der Ausfall eines Fluges ist ein Beispiel dafür. Medientermine, Fotoshootings sind Dinge, die viel Zeit beanspruchen und deshalb Flexibilität in der Trainingsplanung erfordern. Eine weitere Herausforderung ist die Kontinuität in der Aufzeichnung von Trainingsdaten.

Ich: Wie können die Herausforderungen bewältigt werden bzw. was wären digitale Lösungsmöglichkeiten?

IP 4: Wearables bieten gewisse Objektivierungsmöglichkeiten, wenn es herauszufinden gilt, welchen Leistungsstand der Athlet hat. Jedoch sollte dies nur ein ergänzendes Tool sein, das als Hilfsmittel herangezogen wird und der Trainer die subjektive Einschätzung zusätzlich haben sollte. Aktuell ist der Erkenntnisstand, was die Technik betrifft, aus meiner Sicht noch nicht so ausgereift, dass man sich immer zu 100% darauf verlassen kann. Trotzdem ist es sehr wichtig, dass eine Kontinuität in der Aufzeichnung von Trainingsdaten, gerade schon in jüngeren Jahren der Athleten, entsteht. Es ist zudem wichtig, dass Daten aus der Leistungsdiagnostik für Verletzungs- und Überlastungsproblematiken aufgezeichnet und in einer Datenbank abgespeichert werden. Diese Daten kann man dann mit früheren vergleichen und feststellen, ob früher schon einmal Anzeichen vorhanden waren, die auf ähnliche Muster hindeuten.

Ich: Wie ist Ihre Einstellung künstlicher Intelligenz gegenüber? (pro/contra)

IP 4: Aus meiner Sicht muss man als Sportwissenschaftler diesen Dingen gegenüber aufgeschlossen sein. Als Trainer ist man immer auf der Suche nach neuen Dingen, die dem Athleten weiterhelfen können, sich zu verbessern. Künstliche Intelligenz gehört aus meiner Sicht da dazu. Ich sehe KI als Chance, dass man ergänzende Tools zur Seite hat, die die ein oder andere Entscheidung erleichtert.

Ich: Welche Vorerfahrungen bestehen in Bezug auf künstliche Intelligenz im Sport?

IP 4: Teilweise habe ich durch einen Doktoranden, den ich betreut habe, einen kleinen Einblick in das Thema, genauer machine learning, bekommen. Dabei ging es um Wearables, die im American Football eingesetzt wurden und viele Daten aufgezeichnet haben. Man hat dann versucht, diese Daten mithilfe von künstlicher Intelligenz auszuwerten. Zudem gibt es

vermehrt wissenschaftliche Literatur in Bezug auf mögliche Verletzungsprophylaxe durch KI. Ich denke, je mehr Daten es gibt, desto interessanter wird künstliche Intelligenz für gewisse Fragestellungen.

Ich: Besteht grundsätzlich Vertrauen künstlicher Intelligenz gegenüber?

IP 4: Es besteht kritisches Vertrauen. Ich sehe es als ergänzende Information, die durch das Wissen der jeweiligen Person selektiert werden muss.

Ich: Welche Relevanz hat künstliche Intelligenz bisher im Training des Leistungssports?

IP 4: Ich denke, dass künstliche Intelligenz in Zukunft in Bezug auf Datenverarbeitung und Datenauswertung sehr relevant werden kann.

Ich: Welche Anwendung findet künstliche Intelligenz (in welcher Form auch immer) aktuell?

IP 4: Nicht im Trainingsbetrieb. Ich muss aber auch gestehen, dass ich nicht genau weiß, ob die Trainer künstliche Intelligenz für die Trainingsplanung hernehmen. Ich denke aber nicht.

Ich: In welchem Bereich sehen Sie den größten Nutzen von künstlicher Intelligenz im Training des Leistungssports?

IP 4: Ich denke, dass der größte Nutzen in der Auswertung von Big Data unserer Leistungsdiagnostiken, die über viele Jahrzehnte schon durchgeführt wurden, sein wird. Dabei geht es um Ursachenerkennung von Verletzungen und Überbelastungen. Wünschenswert wäre natürlich, dass man sowohl positive als auch negative Auffälligkeiten des Athleten erkennen kann. In der Talentforschung könnte KI einen sehr großen Nutzen darstellen, eben aufgrund der vielen zur Verfügung stehenden Daten, sofern diese Daten dann auch erfasst werden. Die Objektivierung von Erkenntnissen und die Übertragung in andere Umweltbedingungen sind weitere Nutzen von künstlicher Intelligenz.

Ich: Wie wird die Entwicklung von künstlicher Intelligenz in Zukunft eingeschätzt?

IP 4: Aspekte, wie das Erkennen von positiven und negativen Auffälligkeiten des Athleten können durch die KI eine Chance sein, dies in Kombination mit dem Trainer besser festzustellen. Weiters sehe ich durch die KI die Chance bei Sportarten, die eine subjektive Bewertung im Wettkampfgeschehen haben, z.B. Eiskunstlauf oder Geräteturnen. Diese Sportarten werden durch Video sehr gut aufgezeichnet und die Bewertung findet dann sehr subjektiv von Kampfrichtern statt. Vielleicht kann die KI dann aufgrund der schnellen Datenverarbeitung eine Möglichkeit liefern, dass man die Bewertung dann besser objektivieren kann und die Sportart dadurch gerechter wird. Vielleicht führt dies aber wiederum dazu, dass man beispielsweise im Skispringen keine Kampfrichter mehr benötigt, weil die KI genauer analysieren kann, wie gut der Sprung war. Anstatt fünf Kampfrichter sitzen eventuell nur noch drei dort und bewerten das Ergebnis der KI. Vorteil dabei wäre jedoch, dass die Kampfrichter keinen Bias hätten in Bezug auf das Urteil. Während des Spiels muss man sich trotzdem überlegen, ob der Charakter des Sports durch künstliche Intelligenz eher zerstört wird. Ich könnte mir vorstellen, dass es im Leistungssport zukünftig Personen geben sollte, die Spezialisten im Bereich künstliche Intelligenz sind. Die je nach Fragestellung die an der Person arbeitenden Trainerinnen und Trainer beraten können und ihnen Hinweise geben, wie KI verwendet werden kann, um eine Zusatzinformationen in verschiedenen Belangen generieren zu können. Ich denke auch, dass der Bereich um die künstliche Intelligenz ein Thema sein kann, dass den Unterschied in Bezug auf die Leistung von Athleten machen kann.

Ich: Künstliche Intelligenz eher als Hilfsmittel oder Ersatz für Trainer*in?

IP 4: Aktuell braucht es noch einen Trainer. Künstliche Intelligenz kann nicht erkennen, wie es dem Athleten in Bezug auf den Leistungszustand geht. Wenn man ein System entwickelt, bei dem man als Athlet der Software sein Wohlbefinden bekannt geben kann und die KI dann dementsprechend das Training abändert, würde es den Menschen nicht mehr brauchen. Trotzdem kann die KI aktuell aus meiner Sicht den Menschen nicht ersetzen. Im Breitensport würde ich das anders bewerten. Angenommen ich möchte einen Halbmarathon laufen und habe eine gute Einschätzung über mein Level entwickelt und lasse mir dann einen Trainingsplan von einer KI erstellen. Ob der Plan dann ideal geeignet ist für den Athleten spielt im Breitensport nicht wirklich eine große Rolle, sofern nicht wirklich gesundheitlich negative Auswirkungen durch das Abarbeiten des KI-erstellten Trainingsplans entstehen. Im Leistungssport sind der psychologische Aspekt und die menschliche Beziehung durch einen Menschen zu wichtig, um von einer KI ersetzt zu werden.

Ich: Besteht die Sorge, in Zukunft von künstlicher Intelligenz als Trainer*in ersetzt zu werden?

IP 4: Aktuell nicht, da die künstliche Intelligenz bisher nur durch Kombination mit einem Menschen funktioniert, vor allem im Leistungssport. Ich weiß jedoch nicht, wie sich diese Technologien in Zukunft entwickeln werden.

Ich: Wie ist Ihre Einschätzung zu diesen beiden Trainingsplänen?

IP 4: Interessant zu wissen wäre, nach welchem Ziel die Person trainieren möchte. Trainingsplan 1 ist ein allgemeiner Trainingsplan, der sich gegenüber dem 2 im Aufwärmen und Abkühlen grundsätzlich unterscheidet. Grundsätzlich beinhaltet Trainingsplan 2 Grundlagentraining und zwei Einheiten mit intensiveren Trainings, was durchaus für einen Halbmarathon ganz gut geeignet ist, je nachdem welches Fitnesslevel die Person auch hat. Bei Trainingsplan 1 sieht man beginnend in der ersten Woche eher kürzere Distanzen und mit der Zeit dann längere, also ein progressiver Aufbau. Bei Trainingsplan 2 haben wir unterschiedliche Angaben, was die Länge der Einheit betrifft. Die Angabe „oder Ruhetag“ kann zu einer Verwirrung des Athleten führen, da er nicht genau weiß, ob er die Einheit nun machen soll oder nicht. Deshalb ist diese Angabe nicht ganz ideal. Der Plan ist für jemanden, der schon mehr Erfahrung hat im Laufen, besser geeignet. Der Trainingsplan 1 ist für einen Laufanfänger, der noch nicht viel Erfahrung hat, besser geeignet, da 2 aufwendiger in der Trainingsausführung ist und schwieriger zu verstehen ist.

Ich: Vielen Dank für deine Zeit!

IP 4: Gerne!

Interviewpartner 5, 21.12.2023

Ich: Wie lange sind Sie bereits als Trainer*in im Leistungssport aktiv?

IP 5: Ich bin seit 2008 Trainer. Hier am Nachwuchsleistungssport Tirol habe ich aktuell circa 20% Training und 80% organisatorische Dinge inne.

Ich: Wie lange arbeiten Sie bereits in Ihrer Institution?

IP 5: Hier in der Institution Nachwuchsleistungssport Tirol bin ich seit 2016 tätig. Nach wie vor habe ich in meiner eigentlichen Funktion als sportlicher Leiter trotzdem noch eine kleine Trainerfunktion, da ich diesen Bereich nicht komplett abgeben möchte. Jedoch ist das Organisatorische in den letzten Jahren sehr viel mehr geworden. Aktuell betreue ich nur noch die Kletterer von den Leistungssportschulen im Athletikbereich.

Ich: Welche Qualifikationen haben Sie bereits in Ihrer Laufbahn erlangt?

IP 5: Ich habe in Jena an der Friedrich-Schiller-Universität ein Diplom Sportstudium mit Spezialisierung Bewegung und Leistung gemacht. 2008 bin ich dann zum deutschen Schwimmverband und habe dort für zwei Jahre als Assistent für Leistungssport gearbeitet. Dort war ich eigentlich auch nicht Trainer, sondern eher Sportwissenschaftler. Dort habe ich ganz viel in der Diagnostik gearbeitet mit dem IAT und den Olympiazentren zusammen. Für die Nationalmannschaften für die Bundestrainer aus den verschiedenen Bereichen Freiwasser und im Becken in verschiedenen Altersklassen (also Nachwuchs bis hoch zur Spitze) Trainingskurse geplant. Ich war zuständig für die Facilites und die Planung der Wettkampfreisen. Bevor die Wettbewerbe stattgefunden haben, bin ich dort hingereist und habe mir die Dinge angeschaut. Dementsprechend habe ich viele organisatorische Dinge gemacht, die einen sportwissenschaftlichen Background brauchten. Zusätzlich dazu war ich auch mit bei der Trainerausbildung involviert. 2010 bis 2014 war ich in Dresden am Olympiastützpunkt als wissenschaftlicher Mitarbeiter. Dort ebenfalls eher weniger im Trainingsbereich, sondern in der Diagnostik mit viel Videoobjektivierung in den Sportarten Wasserspringen, Shorttrack und Bob, Rodeln und Skeleton. Damals war ich auch viel mit dem deutschen Bob-Team auf der Weltcuptour unterwegs und war dort für die Videodiagnostik an den Bahnen zuständig. In den Sommermonaten war dann auch viel Kraftdiagnostik an den Messplätzen Bob, Rodel und Skeleton dabei. Primär haben wir dort die Starttechnik der Athleten objektiviert. Ursprünglich war ich Skilangläufer und damit Ausdauerathlet und war auf dem Skigymnasium. D.h. für mich war der Kraftbereich etwas Neues und somit auch mein Einstieg in das Trainerdasein. 2015 bin ich dann als Trainer zum Olympiazentrum hier in Innsbruck und das war so der Schritt ins Krafttraining. In meiner Zeit am OZ hatte ich hauptsächlich Ausdauersportarten betreut. Dort war ich jedoch nur ein Jahr, da ich wieder den Zug zu meiner Sportart hatte und ein Angebot vom deutschen Skiverband hatte. Die Stelle war bei mir daheim in Oberwiesental am Sportgymnasium und ich sollte ein Pilotprojekt als Trainer für Langlauf und Biathlon betreuen. Der Skiverband wollte in der Nachwuchsausbildung die technische Ausbildung aus dem Skilanglauf auch für Junge Biathleten gerne umgesetzt haben wollte. Durch private Dinge bin ich dann wieder nach Innsbruck gekommen. Insgesamt kann ich sagen, dass ich einen guten Mix aus Trainersein und der Diagnostik habe. Im Training war ich dabei eher im Nachwuchsbereich und in der Diagnostik eher im Spitzenbereich unterwegs. Bezüglich meiner Qualifikationen habe ich die gesamte Trainerausbildungsschiene des deutschen Skiverbands im Skilanglauf durchlaufen, also habe dort einen A-Schein. Für den Kraftbereich habe ich während meiner Zeit am Olympiazentrum einen Trainerkurs gemacht. Zusätzlich dazu habe ich noch kleinere Zusatzausbildungen wie Kettle-Bell-Ausbildung, die ergänzend im athletischen Bereich ganz gut sind, gemacht. Das sind so die Hauptsachen, die ich gemacht habe.

Ich: Was ist Ihr Spezialgebiet/was liegt Ihnen am meisten in Bezug auf die Trainingsplanung?

IP 5: Der KVÖ hat jetzt aufgrund der Tatsache, dass Klettern jetzt olympisch geworden ist, eine Professionalisierung durchgeführt. Mit diesem Schritt sind sie eben jetzt auch auf uns zugekommen, da wir schon immer Nachwuchsleistunssportschulen betreuen, die Kletterer haben. Diese waren jedoch nie bei uns im Athletiktraining. Der Bedarf, was die athletische Ausbildung schon in den jungen Jahren angeht, ist auch durch das Sinken des Hochleistungsalters der Athleten deutlich gestiegen. Der Verband wollte dann, dass die

Schüler bei uns im athletischen Bereich betreut werden und der Verband macht alles kletterspezifische. Wenn sie bei uns fertig sind, übernehmen dann die Trainer am Olympiazentrum den athletischen Part. Auch dort läuft es so, dass die Athletik vom OZ und Spezifik beim KVÖ übernommen wird. Momentan ist es bei uns so, dass ich die Gruppen zweigeteilt habe, die eine Gruppe ist Speed und die andere Boulder und Vorstieg. Ich treffe mich dann mit den Trainern zum Saisonbeginn Ende September und bespreche die Krafttrainings. Dann wägen wir ab, welche Krafttrainingsblöcke wir machen und dann ist es über das Jahr so, dass wir uns zu Beginn des Kraftblocks mit dem Trainer zusammensetzen und schauen, wie der vergangene Kraftblock abgelaufen ist und dann in die Detailplanung für den nächsten Mesozyklus gehen. Damit sollte dann das Belastungs- und Erholungsgefüge, mit dem was sie im Spezialtraining machen, passen.

Ich: Wie ist der Ablauf von der Planung bis hinzu den fertig erstellten Trainingsplänen?

IP 5: Zum einen werden zunächst die Stärken und Schwächen von der Kletterspezifik des Athleten analysiert. Daraus ergeben sich erste grobe Raster. Dann haben wir seit diesem Jahr eine allgemeine Diagnostik, die wir zu Beginn der Zyklen durchlaufen lassen und bestimmte kletterspezifische Übungen abprüfen. Dort sehen wir dann, ob sich das bestätigt, was der Trainer sagt. Über das Olympiazentrum gibt es dann noch eine kletterspezifische Diagnostik, welche dann Einfluss auf die Trainingsplanung hat.

Ich: Welche spezifischen Indikatoren und Kennzahlen werden verwendet, um die Trainingspläne zu erstellen?

IP 5: Die Ergebnisse aus den Tests sind dann maßgeblich für die Trainingsplanerstellung.

Ich: Werden vorab sportmotorische Tests durchgeführt?

IP 5: Handgriffkrafttests, Beweglichkeitstests und allgemein kletterspezifische Übungen, die am Olympiazentrum diagnostiziert werden.

Ich: Welche digitalen Hilfsmittel werden in der Trainingsplanung verwendet?

IP 5: Nein. Das ist das spannende Thema, an dem wir arbeiten sollten. Für mich ein großer Punkt ist die Trainingsplanung und -dokumentation, weil aus meiner Sicht in vielen Sportarten, nicht nur im Klettern, ganz wenig dokumentiert wird. Das kenn ich aus Deutschland anders. Deshalb kann man nicht sagen, wie viele Kraftstunden der Athlet hat und wie man am besten weiterhin planen sollte. Aktuell haben der Klettertrainer und ich eine gemeinsame Excel-Datei, in der ich die athletischen und er die kletterspezifischen Dinge einträgt. Der Athlet selber ist da aber nicht mit eingebunden. Das Problem in der Trainingsdokumentation ist aus meiner Sicht, dass das immer sehr sportartspezifisch ist. Wir haben natürlich schon Apps, die es auf dem Markt gibt, ausprobiert und aber schlussendlich dann wieder bei Excel landen und das eher verwenden, da dies flexibler ist für das, was wir brauchen. Diese Variabilität kann aus meiner Sicht gerade keine Software leisten. Unser Wunsch wäre eben auch, dass das Olympiazentrum ein gleiches System hat und wir die Daten untereinander weitergeben können und das auch auf Langfristigkeit läuft.

Ich: Wie flexibel ist die Anpassung von Trainingsplänen innerhalb eines bestimmten Zeitraums (Vorbereitung, Wettkampfsaison)?

IP 5: Ich könnte mir vorstellen, dass die Anpassung durch KI besser wird. Momentan ist es so, dass der Faktor Zeit eine Rolle spielt, weil ich auch andere Aufgaben habe. In der Regel mache ich es so, dass ich mich in der Übungsauswahl eher an einem Gruppentraining orientiere. Natürlich haben die Athleten unterschiedliche Gewichte und wenn es ganz gravierend ist, dann hat mal jemand auch einen anderen Plan. Ich betreue z.B. gerade drei Athleten, die alle die gleichen Übungen machen, da ich es zeitlich nicht schaffe, für alle einen unterschiedlichen Plan zu machen. Klar ist die Frage, ob es das schon braucht in dem Alter, aber da wäre die KI sicherlich auch hilfreich. Die Anpassung kann dementsprechend leichter sein durch KI.

Ich: Welche Herausforderungen/Komplikationen bei der Erstellung von Trainingsplänen können auftreten?

IP 5: Aktuell sehen die Trainingspläne so aus, dass die Übung kurz beschrieben ist, ein Bild dabei ist, die Serien angegeben und die Gewichte definiert sind. Momentan ist also keine Interaktion für die Ausführung einer bestimmten Übung vorhanden.

Ich: Wie können die Herausforderungen bewältigt werden bzw. was wären digitale Lösungsmöglichkeiten?

IP 5: Ich habe über die Frage im Vorhinein Gedanken gemacht. Ich denke, da bin ich momentan zu wenig im Thema drin. Ich kann mir gut vorstellen, dass die KI über Video dokumentieren kann. Aber problemorientiert an diesen Umfang, was der Athlet braucht, heranzugehen und daraus einen Plan zu erstellen, das würde ich mir gerne anschauen, wenn das geht, aber vorstellen kann ich mir das aktuell nicht so wirklich. Es ist so komplex, was ein Athlet mitbringt. Die KI müsste wissen, wie sein biologischer Stand und seine Defizite sind und vieles mehr, um die Trainingsplanung richtig durchführen zu können. Es sind also viele Informationen, die ich erstmal der KI geben müsste, damit eine gute Qualität an einem Plan rauskommt. Meine Vorstellungskraft reicht aktuell nicht aus, dass die KI diesen Prozess schneller durchläuft, als wenn ich das selbst machen würde. Eine weitere digitale Lösungsmöglichkeit wäre, dass KI in einem Trainingsplan eine Interaktion schafft, indem eine Übung beispielsweise mit Video gezeigt wird. Ich denke das wäre eine gute Unterstützung für den Athleten. Durch KI könnte ich mir vorstellen, dass die Qualität an Trainingsplänen besser wird, wenn KI auf die gesamte Datenbank mit den Übungen für den einzelnen Athleten zugreifen könnte.

Ich: Wie ist Ihre Einstellung künstlicher Intelligenz gegenüber? (pro/contra)

IP 5: Grundsätzlich bin ich offen dem Thema gegenüber, da ich mir vorstellen kann, dass es Dinge gibt, die künstliche Intelligenz für uns übernehmen kann.

Ich: Welche Vorerfahrungen bestehen in Bezug auf künstliche Intelligenz im Sport?

IP 5: Ich habe künstliche Intelligenz selber schon mal probeweise genutzt und bin erstaunt, was dabei rauskam.

Ich: Besteht grundsätzlich Vertrauen künstlicher Intelligenz gegenüber?

IP 5: Doch, also wäre auf alle Fälle offen. Ich kann auch trotz der künstlichen Intelligenz als Hilfsmittel noch das Kontrollorgan als Trainer sein.

Ich: Welche Relevanz hat künstliche Intelligenz bisher im Training des Leistungssports?

IP 5: Bisher keine Relevanz.

Ich: Welche Anwendung findet künstliche Intelligenz (in welcher Form auch immer) aktuell?

IP 5: Bisher keine Anwendung.

Ich: In welchem Bereich sehen Sie den größten Nutzen von künstlicher Intelligenz im Training des Leistungssports?

IP 5: Wo der Einsatz denke ich auch möglich wäre, ist in der Belastungssteuerung bzw. -feedbackbereich. Also wenn die Athleten im Kraftraum trainieren, natürlich bin ich als Trainer selber dabei, jedoch habe ich dann gut zu tun, wenn 9 Athleten mit unterschiedlichen Trainingsplänen trainieren. In Zukunft werden es sogar noch mehr Athleten, da wir in zwei Jahren fünf Jahrgänge betreuen werden. Also gerade für Feedback kann die künstliche Intelligenz vielleicht in Zukunft helfen. Gerade in Bezug auf die Ausführung einer Übung könnte ich mir vorstellen, dass KI Abhilfe schaffen kann. Zudem kann künstliche Intelligenz in der Trainingsplanung und -dokumentation Abhilfe schaffen, wenn man z.B. eintragen kann, wie trainiert wurde und diese Information dann die nächste Trainingsplanung automatisch anpasst. Außerdem wäre cool, wenn die KI sieht wie viel Gewicht der Athlet gerade stemmt und man das selber nicht mehr händisch eintragen muss. Wichtig zudem ist das Vertrauen zwischen Trainer und Athlet und wenn die jungen Athleten immer mehr mit Technologien zu tun haben, ist es für ihn auch gut zu sehen, wenn der Trainer das auch macht. Die Punkte Zeitmanagement und Zeitersparnis sind sicher auch Themen, die einen großen Nutzen durch KI bringen können.

Ich: Wie wird die Entwicklung von künstlicher Intelligenz in Zukunft eingeschätzt?

IP 5: Das kann ich schwer einschätzen, da ich technisch nicht im Thema bin. Jedoch kann ich mir gut vorstellen, dass da viel möglich sein kann in Zukunft.

Ich: Künstliche Intelligenz eher als Hilfsmittel oder Ersatz für Trainer*in?

IP 5: Als Hilfsmittel gerade im Bereich Trainingsaufarbeitung, um zu sehen, was der Athlet gemacht hat, während ich gerade bei einem anderen war, macht es auf jeden Fall Sinn. Die künstliche Intelligenz wird uns Trainer nicht ersetzen können, da man eben auf zwischenmenschlicher Ebene mit dem Athleten kommunizieren muss. Vielleicht bin ich auch zu blauäugig was das Thema angeht, aktuell kann ich mir das jedoch nicht vorstellen.

Ich: Besteht die Sorge, in Zukunft von künstlicher Intelligenz als Trainer*in ersetzt zu werden?

IP 5: Die künstliche Intelligenz wird uns nicht ersetzen, da habe ich keine Angst. Weil wir ja mit den Menschen arbeiten und irgendwo muss man ja auch kommunizieren und das Feedback geben.

Ich: Wie ist Ihre Einschätzung zu diesen beiden Trainingsplänen?

IP 5: Trainingsplan 1 ist vor dem Hintergrund, dass der Athlet zum ersten Mal einen Halbmarathon laufen möchte, besser geeignet, da er übersichtlicher und einfacher zu verstehen ist als Plan 2. Das Belastungs- und Erholungsgefüge für einen Anfänger ist gut. Plan 2 ist sehr detailliert und hat mit drei Einheiten gleich mal eine hohe Intensität, bei der der Anfänger sehr zu tun hat. Ich weiß nicht, inwiefern der Athlet die 100m Sprints braucht, um den Halbmarathon laufen zu können.

Ich: Vielen Dank für deine Zeit!

IP 5: Gerne, kein Problem!

Interviewpartner 6, 18.01.2024

Ich: Wie lange sind Sie bereits als Trainer*in im Leistungssport aktiv?

IP 6: In dem Bereich, in dem ich jetzt tätig bin, knapp 20 Jahre. Während und nach dem Studium war ich noch als Handballtrainer tätig. Das hat sich aber nach und nach Richtung Athletik verschoben.

Ich: Wie lange arbeiten Sie bereits in Ihrer Institution?

IP 6: Seit 2013 bin ich jetzt in Flensburg, Schwerpunkt war erstmal Jugend mit der Akademie im Nachwuchsbereich. Aktuell bin ich das siebte Jahr bei den Profis.

Ich: Welche Qualifikationen haben Sie bereits in Ihrer Laufbahn erlangt?

IP 6: Ich habe Sportwissenschaften mit Fachbereich Leistungssport in Leipzig studiert. Danach habe ich mich weitergebildet mit Athletiktrainer für Spielsportarten an der Trainerakademie in Köln. Zusätzlich dazu habe ich die A-Lizenz im Bereich Handball und den EHF Mastercoach gemacht. Und ich glaube, das hilft mir gerade bei meiner Tätigkeit weiter, weil man weiß, mit was man es zu tun hat.

Ich: Was ist Ihr Spezialgebiet/was liegt Ihnen am meisten in Bezug auf die Trainingsplanung?

IP 6: Ich bin hauptamtlich für den athletischen Bereich der Handballer mit ein paar Toptalenten zuständig. Also zum einen die Profis und zum anderen die Talente aus unserer Akademie. Über Doktoranden befasse ich mich aktuell zusätzlich den Themen Regenerations- und Belastungsmanagement, das ist bei uns ja auch ein riesen Thema. Zusätzlich dazu kommt das Spielersatztraining für die Spieler, die weniger zum Einsatz kommen. Ich bin genauso Reha-Trainer wie Athletiktrainer, deswegen ist der Aufgabenbereich etwas größer.

Ich: Wie ist der Ablauf von der Planung bis hinzu den fertig erstellten Trainingsplänen?

IP 6: Wenn ich mit Profis zu tun habe, dann bestimmt der Wettkampfkalender den Plan und dieser Kalender steht nicht einmal fest, das ist die Herausforderung. Die Spiele sind bekannt für die nächsten vier bis sechs Wochen, ich weiß natürlich, wann die Saison anfängt und aufhört, aber alles dazwischen weiß ich nicht. Ich weiß zudem im Vorhinein eines Spiels nicht, wie hoch die Belastung des Spielers ist, also wie lang er spielen wird. Wir verfahren nach einer Art dynamischen Periodisierung, d.h. wir wissen, wann die Vorbereitungsphase beginnt, dann haben wir die Hinrunde, Meisterschaft und dann die Rückrunde. Beim Handball ist es leider fast so, dass wir elf Monate Saison haben. Und in diesem Monat, in dem die Spieler frei haben, fahren sie zur EM, WM oder olympische Spiele. Im Groben habe ich vorstrukturiert, wofür ich eine bestimmte Phase nutzen will, es macht aber alles keinen Sinn, wenn ich z.B. merke, der Spieler hat am Wochenende gar nicht gespielt. Dann muss ich neu überlegen, was am meisten Sinn macht. In Bezug auf die Informationsbeschaffung gilt für mich am Anfang und während der Saison zu klären, mit wem habe ich es zu tun. In Bezug auf die Fokussetzung beschreibt es im Minimum vorliegende Faktoren eigentlich ganz gut. Das heißt, ich finde bei jedem Spieler einen Bereich, in dem er sich verbessern kann. In Bezug auf die Fokussetzung ist natürlich durch die Physiotherapeuten und Leistungsdiagnostik maßgeblich, damit wir wissen, wo wir den größten Effekt erzielen. In Bezug auf die Leistungsmerkmale kann ich dir sagen, dass Handball eine sehr komplexe Sportart ist und es egal ist, ob mein Links-Außen zwei Zentimeter höher springt oder nicht. Ich muss dafür sorgen, dass er 66 mal bei 66 Spielen in der Saison springen kann und das 20 mal pro Spiel. Das heißt der Ansatz ist ein bisschen ein anderer. Wichtig ist, dass ich weiß, wann ich diesen Trainingsreiz, den ich setzen möchte, setzen kann und wann nicht. Das Anforderungsprofil ist durch die verschiedenen Positionen sehr unterschiedlich und selbst in Bezug auf eine Position gibt es verschiedene Spielertypen, die unterschiedliche Trainings brauchen.

Ich: Welche spezifischen Indikatoren und Kennzahlen werden verwendet, um die Trainingspläne zu erstellen?

IP 6: Die Indikatoren ergeben sich aus den durchgeführten Leistungsdiagnostiktests.

Ich: Werden vorab sportmotorische Tests durchgeführt?

IP 6: Grundlagenausdauertests, verpflichtende Sportmedizinischen Tests, laufende Sprungdiagnostiken mit Kraftmessplatten, Psychometrie, diese Tests werden durchgeführt.

Während der Spiele nutzen wir die Datenerfassung durch LPS und während des Trainings mit IMU, sodass ich genau weiß, wer wie viel im Training und Spiel gemacht hat, was die Belastung betrifft.

Ich: Welche digitalen Hilfsmittel werden in der Trainingsplanung verwendet?

IP 6: IMU, LSP.

Ich: Wie flexibel ist die Anpassung von Trainingsplänen innerhalb eines bestimmten Zeitraums (Vorbereitung, Wettkampfsaison)?

IP 6: Sehr hoch, also da passiert relativ viel. Ich habe im Vorhinein angepasst auf die Daten, die ich habe, einen Plan für jeden Spieler und gehe vor dem Training noch kurz mit jedem Spieler in den Dialog. Wenn jemand beispielsweise eine Pause durch Verletzung hatte, können wir bei ihm z.B. zwei Durchgänge mehr machen. Oder jemand hatte eine Verletzung im Knie, dann macht dieser Spieler zusätzliche Beinkraftübungen. Da passiert also extrem viel Anpassung und das natürlich auch nur, weil ich jetzt das Glück habe, immer dabei sein zu dürfen. Das ist viel angenehmer als zu der Zeit als ich eine halbe Stelle inne hatte. Es ist also teilweise objektiv durch die Daten, subjektiv durch den Spieler und dann kommen noch meine Erfahrungswerte dazu und dann passt man das dementsprechend an.

Ich: Welche Herausforderungen/Komplikationen bei der Erstellung von Trainingsplänen können auftreten?

IP 6: Ich weiß nicht, was die Spieler in ihrer Freizeit an sportlicher Aktivität machen. Letzte Woche hatte ich einen Spieler, der fällt für das Training aus, weil er drei Stunden Tennis gespielt hat, nachdem er beim Krafttraining war. Die Trainingspläne werden danach gestaltet, je nachdem wie viel Zeit ich für das Training zur Verfügung habe, das ist aufgrund der genannten intensiven Saison und der EM,WM und olympischen Spielen nicht so einfach. Das Problem ist also, dass wir nur eine gewisse Zeit für das Training haben und der Spieler kann nur einen gewissen Load ertragen. Das Anforderungsprofil ist durch die verschiedenen Positionen sehr unterschiedlich und selbst in Bezug auf eine Position gibt es verschiedene Spielertypen, die unterschiedliche Trainings brauchen. Die gleiche Position kann einen komplett anderen Trainingsrhythmus und andere Trainingsbedingungen und Zielsetzung haben. Selbst bei dem gleichen Spieler kann der Trainingsplan unterschiedlich sein, da wahnsinnig viele Sachen mit reinspielen. Die Herausforderung, die jetzt hierbei noch sehe bei diesen künstlichen Intelligenzen was zum Beispiel diese Trainingspläne angeht usw., sie wissen ja eigentlich nicht so richtig was dabei rauskommt, d.h. wenn ich jetzt ChatGPT frage nach einem Trainingsplan, durchsucht er alle Seiten, hämmert da was zusammen und sagt „bei Hypertrophie stand häufig das, da mach ich das und das“. Was Zahlen angeht, macht das ja schon Sinn und ist nicht völlig am Thema vorbei, aber das ist ja eigentlich ein Durchschnitt von dem was er so gefunden hat, ohne zu wissen, was da am Ende dabei herauskommt. Dort sehe ich den Unterschied: während wir der künstlichen Intelligenz sagen, was am Ende dabei rauskommen soll, weiß ChatGPT das noch nicht so richtig, bzw. da habe ich bisher noch keinen gefunden, der mir das sagen kann. Wir reden ja auch mit Wirtschaftsinformatikern, die das System bisschen besser durschauen als wir. Deswegen fehlt mir das aktuell noch ein bisschen, um zu schauen, was kommt am Ende dabei heraus. Diese individualisierte Anpassung an einen einzelnen Spieler.

Ich: Wie können die Herausforderungen bewältigt werden bzw. was wären digitale Lösungsmöglichkeiten?

IP 6: Wir führen deshalb objektive Daten (Belastungsdaten aus dem Spiel und Training) zusammen, um einen Überblick über den Leistungszustand des Spielers zu bekommen. Zudem ist wichtig, dass man ein individuelles Empfinden für einen Spieler entwickelt, damit man die individuellen Anforderungen erfüllen kann. Wir arbeiten mit einem Dashboard, auf dem wir Psychometriedaten, Sprungdaten, Gewichtdaten und Belastungsdaten über das Spiel zusammenführen. Es ist allgemein schwierig zu lösen, da sehr viel über Kommunikation läuft, d.h. ein Schwerpunkt im Training kann nicht dort gesetzt werden, wo der Spieler Probleme hat. Das weiß man aber oft nicht im Vorhinein. Im besten Fall weiß ich möglichst alles über die einzelnen Spieler Bescheid, damit ich einen geeigneten Trainingsplan erstellen kann.

Ich: Wie ist Ihre Einstellung künstlicher Intelligenz gegenüber? (pro/contra)

IP 6: Finde ich super.

Ich: Welche Vorerfahrungen bestehen in Bezug auf künstliche Intelligenz im Sport?

IP 6: Das sage ich auch gern bei Vorträgen: Alles, was wir machen, entstand aus einer Fragestellung heraus. Also wir haben jetzt nicht mit etwas angefangen, weil jemand gesagt hat: „ das ist super“. Glücklicherweise hat die HBL einen Vertrag mit Kinexon gemacht, wobei seitdem alle Spieler während des Spiels einen Sensor im Nacken tragen. Für uns war dann das nächste Problem, dass wir diese Informationen auch beim Training bräuchten. Dann haben wir dort ein IMU-System ins Training implementiert. Das heißt, mit der Zeit wächst dann die Nutzung von solchen Sachen. Auch über Wirtschaftsinformatiker haben wir Erfahrung in Bezug auf die Technik und die Möglichkeiten bekommen. Für uns war immer die Frage: „Das will ich wissen, was kann mir dabei helfen“? Wir haben aber auch viele Wege gehabt, bei denen wir feststellen mussten, dass das so jetzt nicht funktioniert.

Ich: Besteht grundsätzlich Vertrauen künstlicher Intelligenz gegenüber?

IP 6: Man muss aufpassen, dass man den Daten dann nicht zu schnell vertraut. Die Daten sollten schon nochmal hinterfragt werden. Es hilft mir nichts, wenn die KI etwas anderes sagt als der Spieler selbst.

Ich: Welche Relevanz hat künstliche Intelligenz bisher im Training des Leistungssports?

IP 6: Also in dem Begriff, wie ich KI verstehe, würde ich KI erst sehr spät als KI definieren. Gerade in Bezug auf den Film vom ZDF sind viele Dinge dabei, die ich nicht als KI bezeichnen würde. Oftmals wurde dort KI mit Datenverarbeitung verwechselt, gerade im Bereich des Tennis in dem Video. Datenverarbeitung nutzen wir natürlich so oder so, aber KI so wie wir das definieren mit diesen angelernten Algorithmen, ist das im praktischen ein minimaler Bereich, wo wir das nutzen. Bisher hilft uns KI ansonsten nicht genug weiter, dass wir es verwenden könnten. Trotzdem ist es schön zu sehen, wie sich das alles weiterentwickelt. Und gerade durch die Uni, welche die Vollversion für ChatGPT hat, ist es beachtlich, was in Bezug auf die Auswertung möglich ist. KI verwenden wir bisher wirklich nur dort, wo du es in dem Video gesehen hast, nämlich Taktikanalyse. Bezogen auf den Trainingsalltag findet KI wirklich wenig Relevanz bisher. So weit ist es einfach noch nicht.

Ich: Welche Anwendung findet künstliche Intelligenz (in welcher Form auch immer) aktuell?

IP 6: Wir nutzen das auch in Verbindung mit Wirtschaftsmathematikern. Wir haben jetzt schon Sachen entwickelt, wo es darum ging, eigene Sachen anzulernen, um Spieleffektivitäten unser eigener Spieler besser darstellen zu können. Damit wir womöglich einen Input bekommen, taktische Sachen für die Trainingsplanung usw. Ich glaube, dass es hauptsächlich dort genutzt werden muss, wo es um taktische Sachen geht. Ich sehe aktuell noch nicht, wie ich die KI in anderen Bereichen mit den Voraussetzungen und dem Effekt so füttern kann, wie ich das bei Taktiksachen machen kann.

Ich: In welchem Bereich sehen Sie den größten Nutzen von künstlicher Intelligenz im Training des Leistungssports?

IP 6: Zum jetzigen Zeitpunkt kann ich mir keinen größeren Nutzen aus der KI vorstellen als das, was wir sowieso bereits machen. Was Technik und Daten angeht, kann uns das sicher sehr gut unterstützen und uns mehr Zeit mit dem Athleten verschaffen. Aber wie gesagt, in anderen Bereichen wie beispielsweise die Trainingsplanung sehe ich das aktuell als schwierig an.

Ich: Wie wird die Entwicklung von künstlicher Intelligenz in Zukunft eingeschätzt?

IP 6: Da bin ich mal gespannt, vor zehn Jahren hätte ich auch nicht gedacht, was heute alles möglich ist, wirklich möglich sein könnte. Deswegen muss man immer bisschen vorsichtig sein mit seinen Kommentaren, aber ich sehe es hauptsächlich da drin, Muster zu erkennen und Vorschläge, wie ich was trainieren kann und wie man gegen die und die Mannschaften spielen sollte, zu machen. Deswegen wird’s halt auch immer nur eine Hilfe sein können, wenn man die KI richtig ansteuert. Trainingsplanung und andere Bereiche sehe ich als eher schwierig an, für Datenanalyse macht KI auf jeden Fall Sinn.

Ich: Künstliche Intelligenz eher als Hilfsmittel oder Ersatz für Trainer*in?

IP 6: Ich glaube bis zu einem gewissen Teilbereich kann die KI natürlich Sachen genauso anbieten, wie ein Trainer, der extrem weit weg ist oder die Erfahrungswerte nicht so groß sind und wo diese Individualisierung so nicht läuft. Ich habe das aus Spaß bei ChatGPT auch mal gemacht, dass ich mir einen Trainingsplan erstellen lassen hab. Bis zu einem gewissen Grad glaube ich, kann das auf jeden Fall Menschen ersetzen. Irgendwann ist es

dann glaub ich schwierig, wenn diese menschliche Komponente mit reinkommt und auch viel diese Thematik mit reinkommt, wie die KI so viel Know-How haben kann, wie ein Trainer, der da nah dran ist. Also solange es nicht dann mal irgendwann unter die Haut eingepflanzt ist und Stoffwechsel und alles mögliche gleichzeitig miterfasst, ist ja alles super. Und man merkt auch irgendwo sind wir ja doch auch Herden- und soziale Tiere, dass sich manchmal dann Sportler wünschen, das gerne mit einem Menschen zusammen zu machen.

Ich: Besteht die Sorge, in Zukunft von künstlicher Intelligenz als Trainer*in ersetzt zu werden?

IP 6: Nein, ich denke nicht, dass KI einen Trainer ersetzen kann. Aktuell sehe ich zu wenige Möglichkeiten, die durch KI sinnvoll genutzt werden können, um dann einen Trainer zu ersetzen.

Ich: Wie ist Ihre Einschätzung zu diesen beiden Trainingsplänen?

IP 6: Das erste, was mir mal so aufgefallen ist, als ich drübergeflogen bin, ist, dass die Pläne schon sehr sehr unterschiedlich sind. Wenn die jetzt beide für die gleiche Person sein sollen, dann macht einer etwas falsch. Der erste Plan liest sich so, wie ich damals aus meiner runtastic-App bekommen habe. Also so wie man es im Buch schon mal gelesen hat, bis auf die Anzahl der Tage. Bei einem Laufanfänger muss man sich fragen, ob es vier Tage pro Woche braucht, da bin ich mir sicher, dass der nach 5-6 Wochen Probleme haben wird, mit Achillessehne oder sonst irgendwas, weil er insgesamt dann zu viel läuft. Das ist allgemein so ein Plan, bei dem jetzt nicht allzu viel falsch ist, das könnte eine App generiert haben. Bei dem zweiten Plan dachte ich, als ich das nur mit den „Bergsprints“ gelesen habe, dachte ich, das ist zu hohe Intensität. Ich dachte mir nur: „Wenn jemand in der Lage ist, die ersten Wochen zu überleben, dann muss derjenige gar nicht für einen Halbmarathon zu trainieren“. Ich denke, dass Plan 2 sehr wild ist, gleich in Woche 1 mit Bergsprints anzufangen, da hat der Läufer danach doch schon Schmerzen ohne Ende, wenn er sich nicht sogar verletzt. Zusätzlich dazu kommt noch das Ein- und Auslaufen, der Läufer kommt ja auf unglaubliche Umfänge. Deswegen dachte ich eigentlich nur: „wenn jemand diesen Plan cool findet, dann muss er so eine gute Voraussetzung haben, dass er sich für den Halbmarathon gar nicht mehr vorbereiten muss, weil er dann schon ohne diese Vorbereitung und den Plan so fit ist. Die Pausen sind auch oft zu kurz, selbst wenn das noch ein Ruhetag mal dazwischen ist. Es kommt einfach auch sehr stark darauf an, womit wir es zu tun haben. Das meine ich in Bezug auf den Anspruch des Läufers und wie sonst die Rahmenbedingungen sind. Bei Plan 2 würde ich lange suchen, bis ich das einem Sportler in die Hand drücken würde. Bei Trainingsplan 1 würde ich sagen, dass man nichts mit falsch macht. Grundsätzlich ist das das, was bei einem Durschnitt von vielen Trainingsplänen herauskommt, wenn ich das Internet fragen würde.

Ich: Ich bedanke mich recht herzlich für deine Zeit und die Möglichkeit, dass wir ein Gespräch führen konnten.

IP 6: Gerne, kein Problem.

Ich: Tschüss, alles Gute!

IP 6: Bis dann!

Transcripts of the interviews

Interviewpartner 1 (IP 1), 06.12.2023

Me: How long have you been working as a coach in high-performance sports?
IP 1: At the Olympic Training Center since February 2023. Before that, I worked as a self-employed powerlifting coach for three years, and I still do that on the side.
Me: How long have you been working at your institution?
IP 1: Since February 2023. I coach sports such as para-skiing, wrestling, sailing, youth skiing, and cross-country skiing.
Me: What qualifications have you acquired in your career so far?
IP 1: I studied Sports Science for my Bachelor's degree in Konstanz and earned my Master's degree in Sports Science in Innsbruck. I was active in competitive sports for a long time in climbing, mountain biking, and powerlifting. I completed the C-license in mountain biking, the C-license in snowboarding, and the Fitness B-license.
Me: What is your specialty/what is most important to you in terms of training planning?
IP 1: Maximum strength and hypertrophy are my specialties when it comes to training planning.
Me: What is the process from planning to the finished training plans?
IP 1: It really depends on the environment the athlete was in before coming to us. Often, athletes already have specialized trainers, or they may have coaches who handle, for example, endurance training. This means that we need to clarify in advance what training programs are already in place. We then first sit down with the athlete and later with the coaches to discuss the planning. It is important to know what the goals are. My role as a coach is also to integrate all the inputs from the coaches and assess the athlete's workload to identify areas for improvement. The focus is then determined based on the discussions with the coaches.
Me: What specific indicators and metrics are used to create the training plans?
IP 1: We ask how many competitions the athlete plans to participate in this year. Within the training, the RPE (Rate of Perceived Exertion) is important to me, meaning how strenuous the training is for the athlete.
Me: Are sports motor tests conducted in advance?
IP 1: Generally not, we do not require athletes to undergo sports motor tests before they start training with us. Instead, we need to ensure that they are fit for sports, which is confirmed by a doctor. Regular diagnostics such as spiroergometry and lactate tests are conducted at the institution.
Me: What digital tools are used in training planning?
IP 1: I primarily use Excel. Additionally, I use video analysis tools like Quick, velocity-based training tools that measure barbell speed. I also use a database to collect and record data. We use heart rate monitors and rings to track data.
Me: How flexible is the adjustment of training plans within a specific period (preparation, competition season)?
IP 1: This varies from coach to coach. I plan training one week at a time. Then, I wait for feedback from the athlete, and based on that feedback, I plan the next training week. For example, if exercise XY was much harder, I adjust the weight immediately for the next week. If tests are conducted during a training phase, small adjustments are made to the training. If the test is at the end of a training phase, the result significantly influences the next training phase.
Me: What challenges/complications can arise in the creation of training plans?
IP 1: The biggest challenge is load management, as athletes often have two or more training sessions per day, and the input comes not just from one coach but from several. This is the biggest challenge but also my responsibility, to determine what the athlete needs and what they don't.
Me: How can these challenges be addressed, and what digital solutions are possible?
IP 1: Communication is crucial to understand how the athlete is doing. Wellness scores or

stress indices can be used to assess the athlete's stress levels. It is also important to accurately assess the athlete and know when they might be pretending. A digital solution could be integrating the collected data to gain insights into the athlete's condition. SmarterBase is currently being developed to help combine all the data and have it readily available at a glance.

Me: What is your attitude towards artificial intelligence (AI)? (pro/con)

IP 1: Pretty neutral.

Me: What prior experience do you have with artificial intelligence in sports?

IP 1: I personally have no prior experience with artificial intelligence.

Me: Do you generally trust artificial intelligence?

IP 1: When it comes to data analysis alone, I would trust AI. I believe the potential for error is lower with AI in terms of data analysis compared to a human. However, for other things, one might want the freedom to deviate from AI recommendations.

Me: How relevant is artificial intelligence in elite sports training so far?

IP 1: I don't think it is necessarily essential, but it can be a helpful tool. It could be that AI contributes to improving an athlete's performance to some extent.

Me: How is artificial intelligence currently being applied (in any form)?

IP 1: Currently, artificial intelligence is not used at our facility.

Me: In which area do you see the greatest benefit of artificial intelligence in elite sports training?

IP 1: I could imagine AI being beneficial when it comes to analyzing and evaluating data, especially when dealing with large data sets. AI could certainly save a lot of time.

Me: How do you foresee the development of artificial intelligence in the future?

IP 1: I believe this topic will become increasingly important, especially in relation to the large amounts of data that need to be evaluated and filtered.

Me: Would you see AI more as a tool or a replacement for a coach?

IP 1: Definitely not a replacement, but it is certainly well-suited as a tool. I don't think a coach can currently be replaced by technology. Maybe in ten or twenty years, but at the moment, it's not achievable, especially considering the mental aspect. As a coach, you need to respond to an athlete's emotions. The interpersonal aspect is very important in elite sports, including the psychological side.

Me: Do you have any concerns about being replaced by AI as a coach in the future?

IP 1: No, I don’t have that concern at the moment. There are things that cannot be assessed solely objectively but must also be subjectively interpreted by a human. These things need to be reassessed each time.

Me: What is your assessment of these two training plans?

IP 1: In general, Training Plan 1 includes periodization, meaning the plan is divided by each day of the week. There are four training days in each block, divided into base training, interval training, and moderate load. The plan is very general but certainly effective for running a half marathon. Training Plan 2 includes stretching before each session as a warm-up. I don't think that affects half marathon performance; stretching isn't absolutely necessary. Plan 1 provides information about the route during training, whereas Plan 2 is time-based. I think both plans work fundamentally, but they are presented differently. Plan 2 is presented in more detail, while Plan 1 is more general.

Me: Which of the two plans was created by artificial intelligence?

IP 1: Based on the density of information given to the athlete in Training Plan 2, I would conclude that this plan was created by a coach.

Me: Thank you for your time!

IP 1: You're welcome, no problem.

Interviewpartner 2, 13.12.2023

Me: How long have you been working as a coach in high-performance sports?

IP 2: I have been at the Olympic Training Center for almost two years now, but before that, I worked in youth high-performance alpine skiing for two years. So, in total, I've been a coach in high-performance sports for four years, and before that, I was an active competitive athlete myself.

Me: And is alpine skiing your specialty? Do you only coach ski racers?

IP 2: No, like all other coaches here, I don't coach just one sport. There isn't a specific specialty. Of course, as a former ski racer, I tend to have more athletes from alpine skiing, but I also work with various other sports. I am really a general athletic trainer and performance diagnostician.

Me: What qualifications have you acquired in your career so far?

IP 2: I completed my Bachelor's degree in Sports Science here in Innsbruck, and I will submit my Master's thesis in January or February. So, I am not quite finished with my Master's in Sports Science yet. Additionally, I completed basic coach training at BSPA and some specialized coaching courses, though they are less relevant to my current role.

Me: Regarding training planning, what is your specialty?

IP 2: Currently, it's more focused on rehabilitation framework programs. Otherwise, since I come from alpine skiing, I also specialize in training planning for alpine skiing. However, I coach sports like vaulting, breakdancing, and tennis as well. In the past few months, the focus has been on knee injury rehabilitation because I have been working with an injured athlete, giving me more insight into rehab planning.

Me: What is the general process from planning to the final training plans? In terms of information gathering, focus setting, and performance metrics.

IP 2: First, it's important to consider which sport you are coaching; the sport analysis is the first step to creating a plan. It's important to know the requirements of the sport and the athlete, which we determine through initial tests. Then, I review the athlete's annual competition schedule. The training plan should target those competitions. It's crucial to understand the cycle, whether it's a summer or winter sport. Then, the athlete's goals need to be identified: what the athlete wants to achieve and what building blocks need to be established to get there. The athlete's personal goals should carry a lot of weight; we always try to find a consensus. At the Olympic Training Center, we work very interdisciplinarily, collaborating among coaches for an athlete. The center covers everything from athletic and psychological support to nutrition counseling. Performance diagnostic tests are a key component for focus setting in training planning.

Me: Are motor skills tests conducted in advance?

IP 2: Yes, we conduct several motor skills tests. We perform strength tests, speed tests, such as the Gatter Test and Speedy Jump Test for speed, force plate testing, jump strength tests like Counter Movement Jumps, Drop Jumps, and tapping tests for both arms and legs, strength tests with isometric leg press, 1RM tests, and isokinetic tests using Contrex. Additionally, we use tools like Gymaware for force velocity measurement during training.

Me: Are there any digital tools you use for training planning besides the ones mentioned?

IP 2: Yes, I use TrainingPeaks for planning training sessions. I create training sessions traditionally using Numbers or Excel. We also use SmarterBase, which primarily serves as a wellness monitoring tool. Athletes fill it out every morning, indicating how they feel, how much and how well they slept, their muscular condition, and their overall state.

Me: How flexible is the adjustment of training plans within a specific period (preparation, competition season)?

IP 2: In the preparation phase, we have a plan, but the plan rarely works out exactly as expected because we need to consider the athlete's subjective feelings. We must react to

unpredictable things, such as muscle soreness, which requires short-term flexibility. The long-term planning remains consistent, but whether it is exactly three weeks followed by a one-week break or four weeks followed by a one-week break is usually adjusted on short notice. During the competition season, it depends on the sport. Adjustments must be made based on the schedule and any changes. Training content within a week can be continuously adjusted.

Me: What challenges/complications can arise in creating training plans?

IP 2: Firstly, the athlete's own preferences—what they want, how much commitment they show, and how much time they have. Many athletes are also students or have part-time jobs, so they have other commitments. The second point is how much time they can dedicate to their specialty sport. For instance, if a tennis player can train in the hall every day, it's challenging to align athletic components with that. Regeneration plays a major role, and as coaches, we need to ensure that the athlete's workload is appropriate. We always try to get the maximum out of the athlete while focusing on their long-term development, which often creates some overlap.

Me: How can these challenges be addressed, and what digital solutions might help?

IP 2: Building a good relationship with the athlete is extremely important. The athlete should be able to communicate openly with you, and you should also be able to communicate with them without causing offense. Tracking and continuous communication with the athlete are essential tools in overcoming challenges.

Me: What is your attitude towards artificial intelligence (AI)? (pro/con)

IP 2: It can certainly be helpful. However, the human element is missing from my perspective. Personal aspects are lost, but I do believe it can provide support in various situations. It can be a good supplement. One must not forget their own work. Especially in data analysis, it can be a good tool. But I don't think AI is advanced enough yet to be fully trusted.

Me: What prior experience do you have with artificial intelligence in sports?

IP 2: So far, only with ChatGPT. When testing its functions, you notice that the information it provides must be critically examined. I have no experience with AI in our training settings.

Me: Do you generally trust artificial intelligence?

IP 2: Not 100%, more as an auxiliary tool.

Me: What relevance does artificial intelligence currently have in elite sports training?

IP 2: So far, AI has no relevance in our training.

Me: In what form is AI currently applied, if at all?

IP 2: AI is not currently used in our setting.

Me: In which area do you see the greatest benefit of AI in elite sports training?

IP 2: In processing large amounts of data and in data analysis.

Me: How do you assess the future development of artificial intelligence?

IP 2: In the high-performance sports environment, it will remain important to have a relationship at the human level. Quick adaptation is essential to provide optimal training for the athlete.

Me: AI as a tool or a replacement for a coach?

IP 2: I don't think that coaches in high-performance sports can be replaced by AI, as the human component will always be needed. It also requires a human to critically evaluate the information provided by AI.

Me: Are there concerns about being replaced by AI as a coach in the future?

IP 2: No, there are no concerns.

Me: What is your assessment of these two training plans?

IP 2: Training Plan 2 has a lot of information, perhaps even too much. Training Plan 1 is more concise. I understand everything in Training Plan 1. Structurally, Training Plan 1 makes more sense to me. Training Plan 2 has very high intensity. From a structural and understanding perspective, I find Training Plan 1 better. Both plans are suitable for preparing for a half

marathon, but I think Training Plan 1 is easier for the athlete to understand, especially if they are running a half marathon for the first time.

Me: Thank you for your time.

IP 2: You're welcome!

Interviewpartner 3, 15.12.2023

Me: How long have you been working as a coach in high-performance sports?

IP 3: I have been working at the Olympic Training Center since January 1, 2020, so I am almost completing four years here. However, before that, I already had 20 years of professional experience as the head coach in ski jumping in a leadership position.

Me: How long have you been working at your institution?

IP 3: I have been working here at the Olympic Training Center for nearly four years now.

Me: What qualifications have you acquired in your career so far?

IP 3: I started with the Austrian Ski Federation (ÖSV) in ski jumping as the women's coach. I did that for two years. After that, I moved to Italy to the Italian Ski Federation and developed women's ski jumping there from scratch. We started with teenagers, teaching them ski jumping and coaching them over the years until the World Championships in Oslo in 2011. After that, I led Nordic combined in Italy for another two years. Then I spent a year in Switzerland as the head coach for women's ski jumping. Following that, I worked in youth development at the Garmisch-Patenkirchen Ski Club. Earlier, I was a competitive ski jumper myself and attended a sports school. I was part of the Austrian national team and competed briefly in the World Cup. I also studied sports management and completed several coaching certifications, such as trainer education, general coach, specialized ski jumping coach, and Nordic combined instructor. In recent years, I have taken several courses in neuroathletics.

Me: What is your specialty or what do you focus on most in terms of training planning?

IP 3: As an athletic or strength and conditioning coach, we are responsible for various sports. Each coach handles several athletes participating in different sports. Most of my athletes are climbers - four speed climbers, one male bouldering athlete, and one female bouldering athlete (combined), one para-athlete, one gymnast, one taekwondo fighter, and one ski jumper whom I train in neuroathletics. Generally, at our facility, due to organizational reasons, we manage a mix of winter and summer sports. I coordinate with the specialized coach for each athlete. We receive specific guidelines from the specialist coaches that we are expected to follow, but we still have the freedom to incorporate our own inputs into the training plan. Overall, I work with sports where explosive strength is crucial, so that is my core competency. Our institution emphasizes teamwork, ensuring that the areas of expertise in training planning are allocated to the appropriate coaches.

Me: What is the process from planning to the final training plans?

IP 3: Simply put, there is a competition calendar for each athlete. The competitions are then prioritized by the specialist coaches or based on the athlete's wishes. After that, we consider how to achieve these requirements through training. We try to base this on performance diagnostics, meaning we have discussions with the specialist coaches and create a strengths-weaknesses profile for the athlete.

Me: What specific indicators and metrics are used to create the training plans?

IP 3: We mainly use relative values as they are more meaningful than absolute values. We also try to compare values within the group to establish reference points. The advantage of the Olympic Training Center is that we have extensive performance diagnostics data across many years and various sports. For instance, we know that skiers dominate the 1RM squat and that ski jumpers excel in jump strength tests. We then assess how, for example, climbers perform in these tests. With newer sports, the challenge is determining what the target value should be, as there is not yet much data available. It is our task to explore this.

Me: Are motor skills tests conducted in advance?

IP 3: If tests have not already been conducted by the federations in advance, we naturally have the option to test athletes here as well. If we test at the institution, it is not as extensive, but focused on the next area of emphasis. For example, we conduct jump strength tests, maximum strength tests, tapping tests, spiroergometry, ergometry, core strength tests, shoulder rotation tests, bench pulls, and bench presses. We always prioritize the overall

health of the athlete, meaning we take the time to correct muscular imbalances. If performance diagnostic tests reveal deficits, possibly due to injuries, we take the time to address them adequately. However, it is still important for the athlete to perform at their best, provided their well-being is ensured.

Me: What digital tools are used in training planning?

IP 3: Increasingly, wearables are being used to track various parameters, such as heart rate monitoring, heart rate belts, Aura rings, and Whoop bands.

Me: How flexible is the adjustment of training plans within a specific period (preparation, competition season)?

IP 3: Generally, as a coach, you are not encouraged to adjust plans, but this is "daily business." It must be flexible, and if something happens like illness or injury, you need to react. I am somewhat skeptical about adjusting plans based on wearable data, as there is not yet enough long-term experience with wearables, and they should be viewed more as supplementary information. You should follow the training plan for at least a week to see if it works well. Beyond that, it is often based on gut feeling.

Me: What challenges or complications can arise when creating training plans?

IP 3: The challenge is that there is rarely an athlete for whom planning can be based solely on athletic necessity. In Austria, we don't yet have a culture where professional athletes dedicate everything to their sport; they have other commitments. We, as coaches, need to take these things into account. For weekly planning, this means determining in advance how much time the athlete has for training. For instance, as a student, the athlete must estimate in advance when they need more time for their studies.

Me: How can these challenges be addressed, and what digital solutions could help?

IP 3: It is crucial to maintain good time management. As mentioned, it is important to clearly communicate the available training time with the athlete at the start of a training phase. There isn't a program that automatically manages time. However, creating digital training plans using an app-controlled software has made communication easier. The athlete has everything on their phone, allowing them to train remotely if needed. As a coach, I can still track what training was completed and how it was carried out. The software used is called AthleteMonitoring. In Austria, there is currently a major project by the Austrian Olympic Committee to better manage the vast amount of data available for each athlete on one platform. The software is called SmarterBase, originating from Australia, and primarily focuses on managing athlete availability and training capacity. The goal is to reduce injuries.

Me: What is your attitude towards artificial intelligence (AI)? (pro/con)

IP 3: Being one of the older coaches here, I am quite distant from the topic, though I find it extremely interesting. For many standardized tasks, AI could work well. However, in elite sports, training is not standardized. For example, I have four athletes who all receive personalized plans, and even though they all compete in the same events and have the same priorities, it's not a "copy-paste" approach. Seeing the effort needed to incorporate everything, I question whether AI could ever fully take over. It would require a very clean input of data to produce useful output. Maintaining a meaningful data input over a long period is still the hardest part.

Me: What prior experiences do you have with artificial intelligence in sports?

IP 3: I am quite far removed from the topic, but we did have brief contact with AI through a video software that detects body joint points and calculates the center of mass and speed. This software learns over time and can estimate the position of obscured body parts. However, the results are not yet accurate enough to claim it is faster than manual analysis. While manual analysis takes much longer, it's a trade-off. The inaccuracy of AI results can mean they aren't reliable for drawing conclusions. Currently, everyone wants to use AI, but the key is to provide it with highly fact-based information. I believe people are often taking the second step before the first.

Me: Do you generally trust artificial intelligence?

IP 3: In a rather arrogant sense, the simpler the task for AI, the higher my trust in it.
Me: What relevance does AI currently have in elite sports training?
IP 3: We use many standardized calculations in the background, but mainly in the area of statistics.
Me: In what form is AI currently applied?
IP 3: Apart from the software I mentioned, AI is not currently used.
Me: In which area do you see the greatest benefit of AI in elite sports training?
IP 3: The risk with human involvement is the influence of gut feeling, which can interfere, causing actions that are not fact-based. This poses a potential danger, but it is also a human strength that will always surpass AI—empathy. It would be great if my intuition as a coach could be backed by facts from AI, but it might lead to a conflict if the AI suggests something different from the coach's plan.
Me: How do you see the development of AI in the future?
IP 3: I believe AI could work very well in recreational sports in the future, as the information provided by AI would suit the general population. This topic will evolve significantly, and over time, we will learn to trust it more as we gain experience. It was similar when heart rate monitors were new—today, no one questions using a heart rate belt. I imagine it will be the same with AI; at first, there is skepticism, but once sufficient experience is gained, trust will build. However, I think it will take a long time before this point is reached.
Me: AI as a tool or a replacement for a coach?
IP 3: As I mentioned, I believe that in recreational sports, a coach could be replaced by AI. A hobby runner could certainly manage without a coach if they provide their data to AI. At the elite level, I don't think this replacement is possible because the personal relationship and individualized attention are crucial. The burdens on an athlete, beyond just training stress, require trust and communication with the coach.
Me: Are there concerns about being replaced by AI as a coach in the future?
IP 3: I only need to work for twenty more years, so I think I'll make it through. For a recent sports science graduate, I might give a different prognosis. No, I am not worried about being replaced. However, our age difference plays a big role, as I believe our job will eventually be replaced by such technologies for the general population. Without a background in elite sports, it will be difficult to enter this high-performance setting.
Me: What is your assessment of these two training plans?
IP 3: Without reading the training plans in detail, I can say that neither looks appealing or structured. The workout description should not be just two lines long; I always have to look for the distance and intensity. The table should be clear and consistent. In Plan 2, the athlete needs to know themselves well to understand exactly what to do. Without prior experience, this would be difficult. It must be clear for the recipient at their level what to do. There are many variables, making it hard for me as a coach to track what the athlete has actually done. Plan 1 is more concise. It specifies what the athlete needs to do more concretely. Given that both plans were designed for someone running a half marathon for the first time, I would prefer Plan 1, as it has less text and is less intimidating. It's like a recipe—it provides the duration, intensity, and training method, and then you can start. Plan 2 has too many decision points.
Me: Thank you for your time!
IP 3: You're welcome, no problem.

Interviewpartner 4, 19.12.2023

Me: How long have you been working as a coach in high-performance sports?

IP 4: I was never an active coach at the Olympic Training Center. I founded the center and immediately took on the leadership role in 2011. In 2001, I returned to Innsbruck from Salzburg, and from then on, we established the training science center. At that time, my primary role was in performance diagnostics, but I was also interested in directly coaching athletes. We have a very intensive exchange within the team at the Olympic Training Center, and I am responsible for the activities of my employees. However, I was never actively responsible as a coach for a single athlete.

Me: How long have you been working at your institution?

IP 4: I have been working as the director of the Olympic Training Center since 2011.

Me: What qualifications have you acquired in your career so far?

IP 4: I studied sports here in Innsbruck, then moved to Salzburg to complete my doctorate. At the same time, I completed my ski coach training. Additionally, I have taken several advanced courses in training science, especially after initially focusing on biomechanics. When I returned from Salzburg, I started as a lecturer at the sports university in Innsbruck.

Me: What is your specialty or what do you focus on most in training planning?

IP 4: Coming from alpine ski racing, I have primarily focused my research on this sport. At the same time, I value working with a diverse range of athletes from various sports. Whenever there was a need or I was asked, I would coach and advise athletes. I have coached sports such as golf, figure skating, snowboarding, skiing, waterskiing, bobsleigh, and Formula 1. This was always an exciting challenge for me, as I needed to immerse myself in each sport to create the right training plans. In terms of training planning, the conditioning factor is the main area of focus.

Me: What is the process from planning to the final training plans?

IP 4: The most important step is to first identify the requirements of the respective sport and consult with experts from that sport. You need to understand the technical, tactical, psychological, and physical demands of the sport. It also depends on whether the sport is a year-round or seasonal sport. Additionally, it is crucial to consult with people who have been involved in the sport for a long time. Before starting the training plan, you need to know the athlete's current status by analyzing the past season and assessing areas of success and areas needing improvement. Performance diagnostics, depending on the focus, is also vital. Based on this information and the goals set by the coach and the athlete, the planning for the season can begin.

Me: What specific indicators and metrics are used to create the training plans?

IP 4: In terms of the athlete's physical preparation, it is important to rely on scientifically validated tests, ideally with existing normative values for the athletes. For youth athletes, it would be optimal to have normative values for each age group to allow for comparisons and to identify strengths and weaknesses. These insights then guide the focus of the training plan.

Me: Are motor skills tests conducted in advance?

IP 4: It has always been a priority here in Innsbruck to conduct numerous tests. Experts have identified seven to eight tests that make the most sense. These tests include 1RM, squats, isokinetic tests, core strength, leg strength, jump strength, reactive strength, muscular endurance, and agility tests, depending on the sport. The tests vary based on the sport.

Me: What digital tools are used in training planning?

IP 4: The tools essentially include all results obtained from the tests, as they allow comparisons with normative values. Currently, in performance diagnostics, we occasionally use digital tools like the Nearsystem, which has been used with cyclists to analyze their seating position. Otherwise, performance diagnostics are generally conducted using traditional stationary equipment. However, devices are becoming smaller, especially when

considering tools like Gymaware. The use of wearables is the next step, which is employed after training planning as a digital tool to monitor or guide the training.

Me: How flexible is the adjustment of training plans within a specific period (preparation, competition season)?

IP 4: The flexibility in adjusting training plans is what distinguishes a good coach from a less capable one. Developing this intuition requires experience. It is important for the coach to assess when the athlete needs higher intensity to reach the goal and when less can sometimes be more. Additionally, open trust and communication between those involved are crucial to ensure that the athlete can always express how they feel. It is also important to always consider the athlete's current competition schedule when planning the next phase. The aspect of recovery plays a critical role.

Me: What challenges or complications can arise when creating training plans?

IP 4: Adjusting the intensity at each point is definitely a challenge when creating training plans. The coach must be able to quickly assess the impact of a competition on the athlete and determine the necessary recovery processes to restore their full performance capacity. Unforeseen circumstances, such as flight delays, are also challenges that may arise. Media appointments and photo shoots are events that require flexibility in training planning as they consume a lot of time. Another challenge is maintaining consistency in recording training data.

Me: How can these challenges be addressed, and what digital solutions might help?

IP 4: Wearables provide some objectivity when determining the athlete’s performance level. However, they should only be used as a supplementary tool, with the coach’s subjective assessment also considered. Currently, the level of knowledge regarding technology is, in my view, not yet advanced enough to be fully relied upon. Nevertheless, it is essential to ensure consistent recording of training data, particularly from an early age. It is also important to record and store performance diagnostics data related to injuries and overuse issues in a database. This data can then be compared with earlier records to identify patterns indicating similar issues.

Me: What is your attitude towards artificial intelligence (AI)? (pro/con)

IP 4: As a sports scientist, one must be open to these developments. Coaches are always looking for new ways to help athletes improve. AI is part of that. I see AI as an opportunity to have supplementary tools that can facilitate decision-making.

Me: What prior experience do you have with artificial intelligence in sports?

IP 4: I have had a brief insight into the topic through a Ph.D. student I supervised, specifically in the area of machine learning. It involved wearables used in American football to collect a lot of data, which was then analyzed using AI. Additionally, there is increasing scientific literature on injury prevention using AI. I believe that the more data there is, the more relevant AI will become for certain questions.

Me: Do you generally trust artificial intelligence?

IP 4: There is critical trust. I see it as supplementary information that needs to be filtered through the expertise of the person using it.

Me: What relevance does AI currently have in elite sports training?

IP 4: I think AI could become very relevant in the future for data processing and analysis.

Me: How is AI currently being applied, if at all?

IP 4: Not in the training process. However, I have to admit that I do not know whether the coaches use AI for training planning. I don’t think so.

Me: In which area do you see the greatest benefit of AI in elite sports training?

IP 4: I think the greatest benefit will be in analyzing the big data from our performance diagnostics, which have been conducted over many decades. It involves identifying the causes of injuries and overuse. Ideally, it would also detect both positive and negative indicators in athletes. In talent identification, AI could be highly beneficial due to the abundance of available data, provided that this data is collected. The objectivity of findings

and their transfer to other environments are further benefits of AI.

Me: How do you see the development of AI in the future?

IP 4: Aspects such as identifying positive and negative athlete indicators could be improved by AI, in combination with the coach’s assessment. I also see potential in sports where competition outcomes are subjectively judged, such as figure skating or gymnastics. These sports are well-recorded on video, but the scoring is subjective. AI could provide a way to make scoring more objective, thereby making the sport fairer. However, this might lead to situations where, for example, ski jumping no longer requires judges because AI can analyze the jump more accurately. Instead of having five judges, perhaps only three would be needed to validate the AI's result, minimizing bias. However, it is also worth considering whether AI might undermine the character of the sport. In elite sports, I imagine there will be specialists in AI who can advise coaches on how to use AI to generate additional information on various aspects. I also think AI could be a factor that differentiates athletes' performance levels.

Me: AI as a tool or a replacement for coaches?

IP 4: Currently, a coach is still needed. AI cannot yet assess the athlete’s performance status. If a system were developed where an athlete could communicate their well-being to the software and AI could then adjust the training accordingly, a coach might not be necessary. However, in my view, AI currently cannot replace a coach. In recreational sports, I would assess this differently. Suppose I want to run a half marathon and have a good sense of my level; I could then have AI create a training plan. Whether the plan is ideally suited to the athlete doesn’t matter much in recreational sports, as long as it doesn’t result in negativeeffects on the athlete’s health. However, in elite sports, the psychological aspect and the human relationship provided by a coach are too important to be replaced by AI.

Me: Are there concerns about being replaced by AI as a coach in the future?

IP 4: Currently, no, as AI only functions effectively when combined with a human, especially in elite sports. However, I do not know how these technologies will develop in the future.

Me: What is your assessment of these two training plans?

IP 4: It would be interesting to know what the person’s goal is. Training Plan 1 is a general plan that fundamentally differs from Plan 2 in warm-up and cool-down routines. Plan 2 includes base training and two sessions with more intensive training, which is quite suitable for a half marathon, depending on the person’s fitness level. In Plan 1, you can see shorter distances in the first week, gradually increasing over time, indicating a progressive build-up. Plan 2 has varying details on session length. The option "or rest day" could confuse the athlete, as they might not know whether they should complete the session or not. The plan is more suitable for someone with running experience. Training Plan 1 is better for a beginner who has less experience because Plan 2 is more demanding in execution and harder to understand.

Me: Thank you for your time!

IP 4: You’re welcome!

Interviewpartner 5, 21.12.2023

Me: How long have you been working as a coach in high-performance sports?

IP 5: I have been a coach since 2008. At the moment, in the Youth High-Performance Sports Center in Tyrol, my work is about 20% training and 80% organizational tasks.

Me: How long have you been working at your institution?

IP 5: I have been working at the Youth High-Performance Sports Center in Tyrol since 2016. I still have a small coaching role as part of my actual function as the sports director because I don't want to give up that area completely. However, the organizational aspect has increased significantly over the years. Currently, I only coach climbers from the high-performance sports schools in the athletic training sector.

Me: What qualifications have you acquired in your career so far?

IP 5: I studied sports at the Friedrich Schiller University in Jena, specializing in movement and performance. In 2008, I joined the German Swimming Federation and worked there for two years as an assistant in high-performance sports. I was not a coach there; I was more of a sports scientist, primarily working in diagnostics with the IAT and the Olympic centers. I planned training courses for the national teams and head coaches in various disciplines, including open water and pool events across different age groups (from youth to elite level). I was responsible for facilities and competition travel planning. Before the competitions took place, I traveled there to check the setups. I managed many organizational tasks that required a sports science background. I was also involved in coach education. From 2010 to 2014, I worked at the Olympic Training Center in Dresden as a scientific assistant, primarily in diagnostics rather than training, focusing heavily on video analysis in sports like diving, short track, bobsleigh, luge, and skeleton. I traveled extensively with the German bobsleigh team during the World Cup tour, managing video diagnostics at the tracks. In the summer months, I was involved in strength diagnostics for bobsleigh, luge, and skeleton at the measurement stations, primarily objectifying athletes' start techniques. I originally was a cross-country skier and endurance athlete, attending a ski gymnasium. For me, the strength area was new and marked my entry into the world of coaching. In 2015, I joined the Olympic Training Center here in Innsbruck as a coach, which was my step into strength training. During my time at the center, I mainly coached endurance sports. However, I was there for only one year, as I was drawn back to my sport, and I received an offer from the German Ski Federation. The position was in my hometown of Oberwiesenthal at the sports gymnasium, where I was supposed to oversee a pilot project as a coach for cross-country skiing and biathlon. The federation wanted to implement the technical training of cross-country skiing for young biathletes in youth development. Due to personal reasons, I returned to Innsbruck. Overall, I can say that I have a good mix of coaching and diagnostics experience. In coaching, I worked mainly in youth development, while in diagnostics, I worked more in elite sports. Regarding my qualifications, I have completed the full coaching training track for cross-country skiing with the German Ski Federation, earning an A license. In strength training, I completed a coaching course during my time at the Olympic Center. Additionally, I have taken supplementary courses such as a kettlebell certification, which is beneficial for athletic training. Those are the main things I have done.

Me: What is your specialty or focus in training planning?

IP 5: The KVÖ (Austrian Climbing Federation) has professionalized its approach now that climbing is an Olympic sport. With this step, they approached us since we have always supported high-performance sports schools that include climbers. However, these climbers had never participated in our athletic training. The demand for athletic development, even at a young age, has increased significantly due to the decreasing peak performance age of athletes. The federation wanted the students to receive athletic training support from us, while the federation handles everything specific to climbing. Once they complete our program, the trainers at the Olympic Center take over the athletic component. There, too, the athletic training is managed by the center, while the climbing specifics are handled by the KVÖ. Currently, I have divided the groups into speed climbing and bouldering/lead climbing. I meet with the coaches at the start of the season in late September to discuss the strength training blocks. We then decide which strength training blocks to implement. Over the year,

we meet at the start of each block to review the previous one and plan the details for the next mesocycle to align the load and recovery balance with the specialized training.

Me: What is the process from planning to the final training plans?

IP 5: First, the strengths and weaknesses of the athlete’s climbing specifics are analyzed, providing an initial framework. Since this year, we have implemented general diagnostics at the beginning of cycles to assess specific climbing exercises. This allows us to see if the results align with the coach's observations. The Olympic Training Center also conducts climbing-specific diagnostics, which influence the training planning.

Me: What specific indicators and metrics are used to create the training plans?

IP 5: The results from the tests are crucial for creating the training plans.

Me: Are motor skills tests conducted in advance?

IP 5: Yes, tests include grip strength, mobility tests, and general climbing-specific exercises diagnosed at the Olympic Training Center.

Me: What digital tools are used in training planning?

IP 5: No, that is an exciting topic we should work on. For me, training planning and documentation are significant issues because, in many sports, not just climbing, very little is documented. This is different from what I know from Germany. Therefore, it's hard to know how many hours of strength training the athlete has done and how best to plan further. Currently, the climbing coach and I share an Excel file where I enter the athletic details and the coach enters the climbing-specific details. However, the athlete is not involved in this process. In my view, the problem with training documentation is that it is often very sport-specific. We have tested various market apps but usually end up back with Excel because it is more flexible for our needs. No software currently offers the variability we require. Ideally, the Olympic Center would have a unified system to exchange data and work with long-term records.

Me: How flexible is the adjustment of training plans within a specific period (preparation, competition season)?

IP 5: I imagine that adjustments could be improved through AI. Currently, time is a factor, as I have other responsibilities. Generally, I follow a group training model when choosing exercises. Athletes have different weights, and if there is a significant difference, I may give someone a different plan. I am currently coaching three athletes who all do the same exercises because I don’t have the time to create individual plans. Whether this is necessary at their age is debatable, but AI could certainly help. Adjustments could be easier with AI.

Me: What challenges or complications can arise when creating training plans?

IP 5: The current training plans describe the exercise briefly, include a picture, indicate the series, and define the weights. There is no interaction on how to perform a specific exercise.

Me: How can these challenges be addressed, and what digital solutions might help?

IP 5: I have thought about this question in advance. I think I am not deeply involved in this topic yet. I imagine that AI could document through video, but approaching the problem based on the athlete's needs and creating a plan from that is something I would like to explore if possible. However, I currently can’t quite imagine it. It is complex, given an athlete’s biological state, deficits, and more, for AI to conduct training planning properly. A lot of information would need to be provided to AI to produce a high-quality plan. I can’t imagine AI processing this faster than I would. Another digital solution could be for AI to create interaction within a training plan, showing an exercise via video, for example. This would be a good support for the athlete. I believe AI could improve the quality of training plans if it could access the entire database of exercises for each athlete.

Me: What is your attitude towards artificial intelligence (AI)? (pro/con)

IP 5: I am generally open to the topic because I believe there are things that AI can take over for us.

Me: What prior experience do you have with artificial intelligence in sports?

IP 5: I have tested AI myself and am surprised by what it can do.

Me: Do you generally trust artificial intelligence?

IP 5: Yes, I am definitely open to it. Despite AI as a tool, I can still act as the control entity as a coach.

Me: What relevance does AI currently have in elite sports training?

IP 5: So far, none.
Me: How is AI currently applied?
IP 5: It is not currently applied.
Me: In which area do you see the greatest benefit of AI in elite sports training?
IP 5: I think its potential use could be in load management and feedback. For instance, when athletes train in the gym, I am present, but it becomes challenging when nine athletes with different plans are training. In the future, there will be even more athletes, as we will support five age groups in two years. AI could help provide feedback, especially in terms of exercise execution. Additionally, AI could assist in training planning and documentation by automatically adjusting the plan based on logged training information. It would also be great if AI could automatically recognize the weight the athlete is lifting so that it doesn't need to be manually entered. Building trust between the coach and athlete is also important; if young athletes interact more with technology, it is good for them to see that the coach is doing the same. Time management and saving time are significant aspects where AI could provide great benefits.
Me: How do you perceive the development of AI in the future?
IP 5: I find it hard to assess because I am not technically knowledgeable in this area. However, I believe that there could be a lot of potential in the future.
Me: AI as a tool or a replacement for a coach?
IP 5: As a tool, especially in processing training data to track what the athlete did while I was busy with another athlete, it definitely makes sense. AI will not replace coaches because communicating with athletes on a personal level is essential. Perhaps I am being too naive, but I can't imagine it happening right now.
Me: Are there concerns about being replaced by AI as a coach in the future?
IP 5: I'm not afraid of AI replacing us, as we work directly with people and need to communicate and provide feedback.
Me: What is your assessment of these two training plans?
IP 5: Training Plan 1 is more suitable given that the athlete is preparing for their first half marathon, as it is more straightforward and easier to understand than Training Plan. The load and recovery balance for a beginner is appropriate. Plan 2 is very detailed and starts with three sessions at a high intensity, which would be challenging for a beginner. I'm not sure how beneficial 100-meter sprints would be for running a half marathon.
Me: Thank you for your time!
IP 5: You're welcome, no problem!

Interviewpartner 6, 18.01.2024

Me: How long have you been working as a coach in high-performance sports?

IP 6: In the area where I currently work, almost 20 years. During and after my studies, I was still working as a handball coach, but gradually it shifted towards athletic training.

Me: How long have you been working at your institution?

IP 6: I've been in Flensburg since 2013, initially focusing on youth development with the academy in the junior sector. Currently, I am in my seventh year with the professionals.

Me: What qualifications have you acquired in your career so far?

IP 6: I studied sports science with a specialization in high-performance sports in Leipzig. I then furthered my education with a certification as an athletic coach for team sports at the Trainer Academy in Cologne. Additionally, I obtained the A license in handball and the EHF Master Coach certification. I believe these qualifications help me a lot in my current role, as it gives me a solid understanding of the context I am working in.

Me: What is your specialty or focus in training planning?

IP 6: I am mainly responsible for the athletic development of the handball players, focusing on top talents. This includes both the professional players and the talents from our academy. Through Ph.D. students, I am also currently exploring topics such as recovery and load management, which is a huge area for us. Additionally, I manage training for players who don't play as often. I also serve as a rehabilitation coach alongside being an athletic coach, which broadens my responsibilities.

Me: What is the process from planning to the final training plans?

IP 6: When working with professionals, the competition schedule dictates the plan, and this schedule isn't always fixed, which is the challenge. The games are known for the next four to six weeks; I know when the season starts and ends, but everything in between is uncertain. I also don't know beforehand how much load a player will have in a game or how long they will play. We follow a kind of dynamic periodization, meaning we know when the preparation phase starts, then we have the first half of the season, the championship, and then the second half. In handball, unfortunately, the season almost lasts for eleven months, and in the one month when players are free, they go to the European Championship, World Cup, or Olympics. Generally, I have a pre-structured plan for each phase, but it becomes irrelevant if, for example, a player didn't play at all over the weekend. Then I have to rethink what makes the most sense. In terms of information gathering, it's about understanding who I am working with at the beginning and throughout the season. Regarding setting the focus, I find at least one area for each player where they can improve. Focus setting is primarily determined by physiotherapists and performance diagnostics to know where we can achieve the most impact. Handball is a very complex sport, and it doesn't matter if my left winger jumps two centimeters higher; what matters is that he can jump consistently 66 times over 66 games and 20 times per game. The goal is to know when to apply the training stimulus I want to implement and when not to. The requirements differ greatly depending on the position, and even within the same position, there are different types of players who need different training approaches.

Me: What specific indicators and metrics are used to create the training plans?

IP 6: The indicators are based on the performance diagnostic tests conducted.

Me: Are motor skills tests conducted in advance?

IP 6: Yes, we conduct basic endurance tests, mandatory sports medical tests, ongoing jump diagnostics with force plates, and psychometric tests. During games, we use LPS for data collection, and during training, we use IMU systems to monitor the load and activity level of each player precisely.

Me: What digital tools are used in training planning?

IP 6: IMU and LPS.

Me: How flexible is the adjustment of training plans within a specific period (preparation, competition season)?

IP 6: Very high; a lot of adjustments happen. I have a plan tailored to the data I have for each player, and I engage in a quick dialogue with each player before training. If someone, for example, had a break due to injury, we might add two more sets for them. If a player has had

a knee injury, we include additional leg strength exercises. A lot of adaptations happen, which is possible because I have the advantage of being present all the time. This is much more convenient compared to when I had only a part-time position. The adjustment is partly objective based on the data, subjective based on the player, and influenced by my experience, which allows for appropriate modifications.

Me: What challenges or complications can arise when creating training plans?

IP 6: I don’t know what the players do in their free time regarding physical activity. Last week, for example, a player missed training because he played three hours of tennis after weight training. The training plans are designed based on the time available for training, which isn’t easy due to the intensive season and tournaments like the European Championship, World Cup, and Olympics. The challenge is that we have limited time for training, and the player can only handle a certain load. The demands vary greatly depending on the position, and even within the same position, there are different types of players requiring different training. The same position can have a completely different training rhythm, conditions, and goals. Even with the same player, the training plan can differ significantly due to various influencing factors. The challenge with AI, particularly regarding training plans, is that AI doesn’t fully understand the outcomes of its plans. When I ask ChatGPT for a training plan, it searches multiple sources, pieces something together, and suggests it based on common references like "hypertrophy." While it’s not entirely off-topic, it’s an average of what it finds without fully understanding the outcome. That’s the difference: AI executes based on inputs, but it lacks the complete understanding of what the final result should be, which no one has managed to clarify yet. We also consult IT specialists who understand these systems better than we do, but this individualized adaptation to a specific player remains challenging.

Me: How can these challenges be addressed, and what digital solutions might help?

IP 6: We combine objective data (load data from games and training) to get an overview of the player’s performance status. It’s also important to develop an individual understanding of each player to meet their specific needs. We use a dashboard where we consolidate psychometric data, jump data, weight data, and load data from the games. It’s generally challenging, as a lot of it relies on communication, meaning a training focus cannot always be set where the player has issues. Often, this is not known beforehand. Ideally, I have as much information as possible about each player to create a suitable training plan.

Me: What is your attitude towards artificial intelligence (AI)? (pro/con)

IP 6: I think it’s great.

Me: What prior experience do you have with artificial intelligence in sports?

IP 6: As I often say in lectures, everything we do originates from a question. We didn’t start using something because someone said it was great. Fortunately, the HBL has a contract with Kinexon, so all players wear a sensor during games. Our next challenge was implementing this in training, so we added an IMU system. Over time, the use of these tools grows. We also collaborate with IT experts to gain technical insights. Our approach is always: "This is what I want to know; what can help me find out?" However, we’ve also tried many things that didn’t work.

Me: Do you generally trust artificial intelligence?

IP 6: You need to be cautious not to trust data too quickly; it should always be questioned. It’s not helpful if AI says something different than the player himself.

Me: What relevance does AI currently have in elite sports training?

IP 6: From my understanding, I would only define AI as such in a limited sense. There are many things that people call AI, which I see more as data processing, especially in tennis, as shown in a ZDF documentary. We use data processing, but AI in the sense of machine learning algorithms is currently minimal in practical use. AI hasn’t provided us with enough value yet to fully utilize it, although it’s interesting to see the progress. With the university’s full version of ChatGPT, it’s impressive to see what’s possible with analysis. Currently, we use AI mainly for tactical analysis, as shown in the video. In everyday training, AI has little relevance; it’s simply not that advanced yet.

Me: How is AI currently applied?

IP 6: We work with IT specialists to develop tools that improve tactical efficiency analysis for our players, providing inputs for tactical adjustments and training plans. I believe it should

primarily be used in tactical areas. I don’t yet see how AI can be effectively applied to other areas, such as training planning, as I can with tactics.
Me: In which area do you see the greatest benefit of AI in elite sports training?
IP 6: At this point, I cannot imagine a greater benefit beyond what we already do. In terms of technology and data, AI can support us well and give us more time with the athletes. However, I see challenges in other areas, like training planning. For data analysis, AI certainly makes sense.
Me: How do you view the development of AI in the future?
IP 6: I’m curious; ten years ago, I would not have imagined what is possible today. Therefore, one has to be cautious with predictions, but I primarily see AI’s role in identifying patterns and making training or tactical recommendations based on that. It will always serve as an aid if used correctly, but I find it difficult to see its application in training planning and other areas. For data analysis, AI definitely makes sense.
Me: AI as a tool or a replacement for a coach?
IP 6: I believe AI can certainly handle some tasks like a coach, especially when a coach is not close to the player or lacks experience, where personalization is not happening. I tested it for fun by having ChatGPT create a training plan, and to some extent, it could replace people. However, it becomes difficult when the human element comes into play, such as the experience and close connection a coach has. Unless AI eventually becomes so advanced that it can track metabolism and other physiological factors directly, the human component remains crucial. Athletes, as social beings, sometimes prefer to train with a human.
Me: Are you concerned that AI could replace you as a coach in the future?
IP 6: No, I don’t think AI can replace a coach. Currently, I see too few viable AI applications that could effectively replace a coach.
Me: What is your assessment of these two training plans?
IP 6: The first thing I noticed is that the plans are very different. If they are both supposed to be for the same person, one of them is wrong. The first plan reads like something I might get from my Runtastic app—basic, as seen in books, except for the number of days. For a beginner runner, four days a week might be too much; I’m sure that after 5-6 weeks, there will be issues like Achilles tendon problems because of the overall volume. This plan is generic, and while it isn’t entirely wrong, it looks like something an app could generate. As for the second plan, when I saw "hill sprints," I thought the intensity was too high. If someone likes this plan, they must already be in such good shape that they don't really need to prepare for the half marathon, as they would already be fit enough without this plan. Hill sprints in week 1 are too much; the runner could be in pain afterward or even get injured. The warm-up and cool-down add to the already extensive distances. If someone finds this plan suitable, they likely don’t need much preparation for a half-marathon. The breaks are also often too short, even when a rest day is added. It depends heavily on the athlete's fitness level and other conditions. I would be hesitant to give Plan 2 to an athlete, with Plan 1 there is little room for error; it represents a general average of many plans you would find on the internet.
Me: Thank you very much for your time and for this opportunity to have a conversation.
IP 6: You’re welcome, no problem.
Me: Goodbye, all the best!
IP 6: See you, take care!
