# Supplementary material for: Acceptance and trust in AI-generated exercise plans among recreational athletes and quality evaluation by experienced coaches: a pilot study
Source: BMC Res Notes. 2025 Mar 13;18:112. doi: 10.1186/s13104-025-07172-9 (PMC11908068; doi:10.1186/s13104-025-07172-9)
Supplement: Supplementary file 5 — Supplementary Material 4 [file 13104_2025_7172_MOESM5_ESM.pdf]

Fragebogen (Questionnaire)

Page/ Question	Original	Translated
1	Liebe*r Untersuchungsteilnehmer*in!<br>Im Rahmen meiner Abschlussarbeit des Studiums Sportmanagement an der Universität Innsbruck beschäftige ich mich damit, inwiefern Sportler*innen aus verschiedenen Bereichen Trainingsplanen vertrauen, die mittels künstlicher Intelligenz erstellt wurden Für die Analyse ist Ihre Sicht als Sportle*in sehr wichtig!<br>Die Befragung ist anonym! Alle Ihre Angaben werden streng vertraulich behandelt, es können keine Ruckschlüsse auf einzelne Personen gezogen werden. Eine individuelle Auswertung ist aufgrund der Anonymisierung nicht möglich Im Voraus vielen Dank dafür, dass Sie sich die Zeit zur Beantwortung der Fragen nehmen!	Dear Participant,<br>As part of my thesis in Sports Management at the University of Innsbruck, I am investigating the extent to which athletes from various fields trust training plans created using artificial intelligence. Your perspective as an athlete is highly valuable for this analysis!<br>The survey is anonymous! All your responses will be treated with strict confidentiality, and no conclusions can be drawn about individual participants. Due to the anonymization, individual evaluations are not possible.<br>Thank you in advance for taking the time to answer the questions!
2/1	**Welcher Sportart gehen Sie hauptsächlich nach?**<br>Bitte geben Sie die Sportart an, die sie hauptsächlich durchführen. Wenn Sie mehrere Sportarten ausüben, wählen Sie die Sportart für die Sie am meisten trainieren.<br>- Laufen (Straße, Trail, …)<br>- Radfahren (MTB, Rennrad, …)<br>- Schwimmen<br>- Triathlon<br>- Leichtathletik<br>- Krafttraining<br>- Klettern<br>- Kampfsport<br>- Ski (Alpin, Langlauf, Freeride, Mountaineering, …)<br>- Snowboard<br>- Fußball<br>- Handball<br>- Andere: _____<br>- keine Angabe	**Which sport do you primarily participate in?**<br>Please specify the sport you primarily engage in. If you practice multiple sports, select the one you train for the most.<br>- Running (road, trail, etc.)<br>- Cycling (MTB, road bike, etc.)<br>- Swimming<br>- Triathlon<br>- Track and field<br>- Strength training<br>- Climbing<br>- Martial arts<br>- Skiing (alpine, cross-country, freeride, mountaineering, etc.)<br>- Snowboarding<br>- Soccer<br>- Handball<br>- Other: _____<br>- Prefer not to say
2/2	**Wie oft pro Woche treiben Sie durchschnittlich Sport?**<br>Beziehen Sie die Anzahl bitte auf die Trainingseinheit/en.<br>- Weniger als 1 mal pro Woche<br>- 1 bis 2 mal pro Woche	**How often do you exercise per week on average?**<br>Please refer to the number of training sessions.<br>- Less than once per week<br>- 1 to 2 times per week

	- 3 bis 4 mal pro Woche<br>- 5 bis 6 mal pro Woche<br>- Mehr als 6 mal pro Woche<br>- keine Angabe	- 3 to 4 times per week<br>- 5 to 6 times per week<br>- More than 6 times per week<br>- Prefer not to say
2/3	**Wie lange trainieren Sie durchschnittlich pro Trainingseinheit?**<br>- Weniger als 30 Minuten<br>- 30 - 60 Minuten<br>- 60 - 90 Minuten<br>- 90 - 120 Minuten<br>- Mehr als 120 Minuten<br>- keine Angabe	**How long is your average training session?**<br>- Less than 30 minutes<br>- 30 - 60 minutes<br>- 60 - 90 minutes<br>- 90 - 120 minutes<br>- More than 120 minutes<br>- Prefer not to say
2/4	**Trainieren Sie nach einem Trainingsplan?**<br>- Ja<br>- Nein	**Do you follow a training plan?**<br>- Yes<br>- No
3	**Definition von Künstlicher Intelligenz**<br><br>Im Folgenden wird der Begriff Künstliche Intelligenz (KI) definiert, wie er in dieser Studie verwendet wird. Ein KI-System ist ein maschinenbasiertes System, das Empfehlungen geben, Prognosen erstellen oder Entscheidungen treffen kann. Es verwendet maschinelle oder menschliche Eingaben, um eine reale oder virtuelle Umgebung zu erfassen. Auf dieser Grundlage werden Modelle erstellt und Informationen oder Handlungsoptionen ermittelt. KI-Systeme können mit einem unterschiedlichen Grad an Autonomie ausgestattet sein.<br><br>Diese Funktionen werden auch bei der Erstellung von Trainingsplanen im Sport genutzt.	**Definition of Artificial Intelligence**<br><br>The following definition of Artificial Intelligence (AI) is used in this study. An AI system is a machine-based system that can provide recommendations, make predictions, or make decisions. It processes machine or human inputs to analyze a real or virtual environment. Based on this, models are created to generate information or determine possible courses of action. AI systems can operate with varying degrees of autonomy.<br><br>These functions are also used in the creation of training plans in sports.
4/1	**Wird nur angezeigt wenn bei 2/4 Antwort mit „Ja".**<br><br>**Verwenden Sie momentan einen Trainingsplan, der mittels Künstlicher Intelligenz erstellt wurde?**<br>- Ja<br>- Nein	**This question is only displayed if question 2/4 was answered with "Yes."**<br><br>**Are you currently using a training plan created by artificial intelligence?**<br>- Yes<br>- No
5/1	**Wird nur angezeigt wenn bei 4/1 Antwort mit „Ja".**<br><br>**Verwenden Sie einen der folgenden Tools zur Erstellung eines KI-generierten Trainingsplans?**<br>Wenn Sie das Tool für andere Zwecke – wie die Aufzeichnung von Leistungsdaten	**This question is only displayed if question 4/1 was answered with "Yes."**<br><br>**Do you use any of the following tools to create an AI-generated training plan?**

	– nutzen, so kreuzen Sie es bitte NICHT an!<br>- ChatGPT<br>- Enduco<br>- Freeletics<br>- Strava<br>- Twalv<br>- VIPerform<br>- Andere: _____	If you use the tool for other purposes - such as tracking performance data - please do NOT select it!<br>- ChatGPT<br>- Enduco<br>- Freeletics<br>- Strava<br>- Twalv<br>- VIPerform<br>- Other: _____
6/1	**Einstellung**<br><br>5-Punkte Likert-Skala<br>1 = Stimme überhaupt nicht zu<br>5 = Stimme vollkommen zu<br><br>**Bitte geben Sie an, inwiefern Sie den folgenden Aussagen zustimmen.**<br><br>Ich stehe KI-Systemen zur Trainingsplanung positiv gegenüber.<br><br>Ich empfinde die Nutzung von KI-Systemen zur Trainingsplanung als angenehm.<br><br>Die Verwendung von KI-Systemen zur Trainingsplanung ist eine gute Idee<br><br>Die Verwendung von KI-Systemen zur Trainingsplanung ist eine intelligente Art, um die Trainingsplanung durchzuführen	**Attitude**<br><br>5-Point Likert Scale<br>1 = Strongly disagree<br>5 = Strongly agree<br><br>**Please indicate the extent to which you agree with the following statements.**<br><br>I have a positive attitude toward AI systems for training planning.<br><br>I find the use of AI systems for training planning enjoyable.<br><br>Using AI systems for training planning is a good idea.<br><br>Using AI systems for training planning is an intelligent way to conduct training planning.
6/2a	**Wird nur angezeigt wenn bei 4/1 Antwort mit „Ja“.**<br><br>**Wahrgenommene Benutzerfreundlichkeit**<br><br>**Nutzer**<br><br>5-Punkte Likert-Skala<br>1 = Stimme überhaupt nicht zu<br>5 = Stimme vollkommen zu<br><br>**Bitte geben Sie an, inwiefern Sie den folgenden Aussagen zustimmen.**<br><br>Ich finde die Nutzung von KI-Systemen zur Trainingsplanung einfach.<br><br>Ich finde es einfach, mit KI-Systemen meine Trainingsplanung durchzuführen	**This section is only displayed if question 4/1 was answered with "Yes."**<br><br>**User-Friendliness**<br><br>**User**<br><br>5-Point Likert Scale<br>1 = Strongly disagree<br>5 = Strongly agree<br><br>**Please indicate the extent to which you agree with the following statements.**<br><br>I find using AI systems for training planning easy.<br><br>I find it easy to conduct my training planning with AI systems.

	Die Trainingsplanung mit KI-Systemen ist klar und verständlich.<br><br>Ich bin kompetent in der Trainingsplanung mit KI-Systemen.<br><br>Ich finde KI-Systeme zur Trainingsplanung einfach zu bedienen.<br><br><br>**Wahrgenommene Nützlichkeit**<br><br>**Nutzer**<br><br>Bitte geben Sie an, inwiefern Sie den folgenden Aussagen zustimmen.<br><br>KI-Systeme helfen mir dabei, meine Trainingsplanung schneller zu erledigen.<br><br>KI-Systeme verbessern meine Leistung bei der Trainingsplanung.<br><br>KI-Systeme steigern meine Produktivität bei der Trainingsplanung.<br><br>KI-Systeme erhöhen meine Effektivität bei der Trainingsplanung.<br><br>Ich finde die Verwendung von KI-Systeme zur Trainingsplanung nützlich.<br><br><br>**Nutzungsintention**<br><br>**Nutzer**<br><br>Bitte geben Sie an, inwiefern Sie den folgenden Aussagen zustimmen.<br><br>Ich beabsichtige, KI-Systeme zur Trainingsplanung weiterhin zu nutzen.<br><br>Ich treffe Entscheidungen zur Trainingsplanung basierend auf Empfehlungen von KI-Systemen.<br><br>KI-Systeme zur Trainingsplanung zu nutzen ist etwas, dass ich in Zukunft weiterhin tun werde.	Training planning with AI systems is clear and understandable.<br><br>I feel competent in training planning with AI systems.<br>I find AI systems for training planning easy to use.<br><br><br>**Usefulness**<br><br>**User**<br><br>Please indicate the extent to which you agree with the following statements.<br><br>AI systems help me complete my training planning faster.<br><br>AI systems improve my performance in training planning.<br><br>AI systems increase my productivity in training planning.<br><br>AI systems enhance my effectiveness in training planning.<br><br>I find the use of AI systems for training planning beneficial.<br><br><br>**Intention to Use**<br><br>**User**<br><br>Please indicate the extent to which you agree with the following statements.<br><br>I intend to continue using AI systems for training planning.<br><br>I make training planning decisions based on AI system recommendations.<br><br>Using AI systems for training planning is something I will continue doing in the future.
6/2b	**Wird nur angezeigt wenn bei 4/1 Antwort mit „Nein“.**	**This section is only displayed if question 4/1 was answered with "No."**

	**Wahrgenommene Benutzerfreundlichkeit**<br><br>**Nicht Nutzer**<br><br>5-Punkte Likert-Skala<br>1 = Stimme überhaupt nicht zu<br>5 = Stimme vollkommen zu<br><br>Bitte geben Sie an, inwiefern Sie den folgenden Aussagen zustimmen.<br><br>Die Nutzung von KI- Systemen zur Trainingsplanung wäre für mich einfach zu erlernen.<br><br>Ich fände es einfach, mit KI-Systemen meine Trainingsplanung durchzuführen.<br><br>Die Trainingsplanung mit KI-Systemen wäre klar und verständlich.<br><br>Es wäre einfach für mich, kompetent in der Trainingsplanung mit KI-Systemen zu werden.<br><br>Ich fände KI-Systeme zur Trainingsplanung einfach zu bedienen.<br><br><br>**Wahrgenommene Nützlichkeit**<br><br>**Nicht Nutzer**<br><br>Bitte geben Sie an, inwiefern Sie den folgenden Aussagen zustimmen.<br><br>KI-Systeme würden mir dabei helfen meine Trainingsplanung schneller zu erledigen.<br><br>KI-Systeme würden meine Leistung bei der Trainingsplanung verbessern.<br><br>KI-Systeme würden meine Produktivität bei der Trainingsplanung steigern.<br><br>KI-Systeme würden meine Effektivität bei der Trainingsplanung erhöhen.<br><br>Ich fände die Verwendung von KI-Systemen zur Trainingsplanung nützlich.	**User-Friendliness**<br><br>**Non-Users**<br><br>5-Point Likert Scale<br>1 = Strongly disagree<br>5 = Strongly agree<br><br>Please indicate the extent to which you agree with the following statements.<br><br>Using AI systems for training planning would be easy for me to learn.<br><br>I would find it easy to conduct my training planning with AI systems.<br><br>Training planning with AI systems would be clear and understandable.<br><br>It would be easy for me to become competent in training planning with AI systems.<br><br>I would find AI systems for training planning easy to use.<br><br><br>**Usefulness**<br><br>**Non-Users**<br><br>Please indicate the extent to which you agree with the following statements.<br><br>AI systems would help me complete my training planning faster.<br><br>AI systems would improve my performance in training planning.<br><br>AI systems would increase my productivity in training planning.<br><br>AI systems would enhance my effectiveness in training planning.<br><br>I would find the use of AI systems for training planning beneficial.<br><br><br>**Intention to Use**

	**Nutzungsintention**<br><br>**Nicht Nutzer**<br><br>Bitte geben Sie an, inwiefern Sie den folgenden Aussagen zustimmen.<br><br>Ich beabsichtige, KI-Systeme zur Trainingsplanung in Zukunft zu nutzen<br><br>Ich beabsichtige, Entscheidungen zur Trainingsplanung basierend auf Empfehlungen von KI-Systemen zu treffen.<br><br>KI-Systeme zur Trainingsplanung zu nutzen ist etwas, dass ich in Zukunft tun würde.	**Non-Users**<br><br>Please indicate the extent to which you agree with the following statements.<br><br>I intend to use AI systems for training planning in the future.<br><br>I intend to make training planning decisions based on AI system recommendations.<br><br>Using AI systems for training planning is something I would do in the future.
7	**Vertrauen**<br><br>5-Punkte Likert-Skala<br>1 = Stimme überhaupt nicht zu<br>5 = Stimme vollkommen zu<br><br>**Bitte geben Sie an, inwiefern Sie den folgenden Aussagen zustimmen.**<br><br>KI-Systeme für die Trainingsplanung sind kompetent in der Bereitstellung von Informationen und Beratung, die ich brauche.<br><br>KI-Systeme für die Trainingsplanung sind zuverlässig und liefern konsistente und verlässliche Informationen.<br><br>KI-Systeme für die Trainingsplanung sind transparent.<br><br>KI-Systeme für die Trainingsplanung sind verlässlich und glaubwürdig.<br><br>KI-Systeme für die Trainingsplanung werden ihre Antworten nicht manipulieren und keine negativen Konsequenzen für mich erzeugen.<br><br>KI-Systeme für die Trainingsplanung handeln in guter Absicht und sind ehrlich zu mir.	**Trust**<br><br>5-Point Likert Scale<br>1 = Strongly disagree<br>5 = Strongly agree<br><br>**Please indicate the extent to which you agree with the following statements.**<br><br>AI systems for training planning are competent in providing the information and advice I need.<br><br>AI systems for training planning are reliable and provide consistent and trustworthy information.<br><br>AI systems for training planning are transparent.<br><br>AI systems for training planning are dependable and credible.<br><br>AI systems for training planning will not manipulate their responses and will not create negative consequences for me.<br><br>AI systems for training planning act with good intentions and are honest with me.<br><br>AI systems for training planning are secure and protect my privacy and confidential information.

	KI-Systeme für die Trainingsplanung sind sicher und schützen meine Privatsphäre und vertrauliche Informationen.<br><br>Ich vertraue Trainingsplanen, die mittels künstlicher Intelligenz erstellt wurden.<br><br>Ich vertraue KI-Systemen für die Trainingsplanung.	I trust training plans created by artificial intelligence.<br><br>I trust AI systems for training planning.
8	**Vergleich Trainingspläne**<br><br>**Trainingsplan Beispiel**<br><br>Im Folgenden werden Ihnen zwei Trainingspläne präsentiert.<br>Es handelt sich jeweils um einen 12-wöchigen Halbmarathon-Trainingsplan, von dem Ihnen einfachheitshalber die ersten beiden<br>Wochen gezeigt werden.<br><br>Diese sind für eine Person ausgelegt, die:<br>- durchschnittich sportlich ist und<br>- ihren ersten Halbmarathon Lauft und<br>- zum Ziel hat diesen zu schaffen<br><br>Lesen Sie sich die Trainingspläne bitte durch und beantworten Sie die darunter stehende Frage.<br><br>*Randomisierung Reihenfolge Trainingsplan X und Y.*	**Comparison of Training Plans**<br><br>**Training Plan Example**<br><br>Below, you will be presented with two training plans.<br>Each is a 12-week half marathon training plan, with only the first two weeks shown for simplicity.<br><br>These plans are designed for a person who:<br><br>Is moderately athletic and<br>Is running their first half marathon, aiming to complete it.<br>Please read through the training plans and answer the question below.<br><br>*Randomized order of Training Plan X and Y.*
9	**Trainingsplan X**<br><br>5-Punkte Likert-Skala<br>1 = Stimme überhaupt nicht zu<br>5 = Stimme vollkommen zu<br><br>**Bitte geben Sie an, inwiefern Sie der folgenden Aussage zustimmen.**<br><br>Ich vertraue diesem Trainingsplan.	**Training Plan X**<br><br>5-Point Likert Scale<br>1 = Strongly disagree<br>5 = Strongly agree<br><br>**Please indicate the extent to which you agree with the following statement.**<br><br>I trust this training plan.
10	**Trainingsplan Y**<br><br>5-Punkte Likert-Skala<br>1 = Stimme überhaupt nicht zu<br>5 = Stimme vollkommen zu<br><br>**Bitte geben Sie an, inwiefern Sie der folgenden Aussage zustimmen.**	**Training Plan Y**<br><br>5-Point Likert Scale<br>1 = Strongly disagree<br>5 = Strongly agree<br><br>**Please indicate the extent to which you agree with the following statement.**

<table>
<tr><td></td><td>Ich vertraue diesem Trainingsplan.</td><td>I trust this training plan.</td></tr>
<tr><td>11</td><td>Soziodemografische Daten

Welchem Geschlecht fühlen Sie sich zugehörig?
- Männlich
- Weiblich
- Divers
- Keine Angabe

Welches ist Ihre höchste abgeschlossene Ausbildung?
- Pflichtschule
- Berufsschule (Lehre)
- Weiterführende Schule ohne Matura / Abitur (Handelsschule, Fachschule, …)
- Weiterführende Schule mit Matura / Abitur (Gymnasium, HAK, HTL,)
- Universität: Bachelor
- Universität: Master, PHD
- Anderes: _____
- keine Angabe

Welcher Tätigkeit gehen Sie momentan hauptsächlich nach?
- Schüler*in
- Student in
- Erwerbstätig (weniger als 20 h/Woche)
- Erwerbstätig mehr als 20 h/Woche)
- Ohne Beschäftigung
- Anderes: _____
- keine Angabe

Wo befindet sich momentan Ihr Lebensmittelpunkt?
- Österreich
- Deutschland
- Italien
- Anderes: _____
- keine Angabe</td><td>Sociodemographic Data

What gender do you identify with?
- Male
- Female
- Non-binary
- Prefer not to say

What is your highest completed education?
- Compulsory education
- Vocational school (apprenticeship)
- Secondary school without high school diploma (business school, technical school, etc.)
- Secondary school with high school diploma (gymnasium, vocational school, technical school, etc.)
- University: Bachelor's degree
- University: Master's degree, PhD
- Other: _____
- Prefer not to say

What is your current main occupation?
- Student
- University student
- Employed (less than 20 hours/week)
- Employed (more than 20 hours/week)
- Unemployed
- Other: _____
- Prefer not to say

Where is your current place of residence?
- Austria
- Germany
- Italy
- Other: _____
- Prefer not to say</td></tr>
<tr><td>12</td><td>Abschluss

Vielen Dank für Ihre Teilnahme!

Sie sind am Ende des Fragebogens angekommen.</td><td>Conclusion

Thank you for your participation!

You have reached the end of the questionnaire.</td></tr>
</table>

	Ich möchte mich herzlich bei Ihnen für das Beantworten der Fragen und die damit verbundene Unterstützung meiner Abschlussarbeit bedanken.<br><br>Alles Gute!<br><br>Ihre Antworten wurden gespeichert, Sie können das Browser-Fenster nun schließen.	I would like to sincerely thank you for answering the questions and for supporting my thesis.<br><br>All the best!<br><br>Your responses have been saved, and you can now close the browser window.

Trainingsplan X German

Woche 1-2	Aufwärmen	Workout Beschreibung	Abkühlen
**Montag**	5 Minuten aktives Dehnen	Lockeres Laufen bei 60 bis 65% der max. Herzfrequenz – 20 bis 30 Minuten	5 Minuten Dehnen
**Dienstag**	5 Minuten aktives Dehnen	Lockeres Laufen oder Crosstraining bei 60 bis 65% der max. Herzfrequenz – 20 bis 30 Minuten oder Ruhetag	5 Minuten Dehnen
**Mittwoch**	2 bis 3 km, 5 Minuten aktives Dehnen	6 bis 8 x 100m-Bergsprints, zur Regeneration jeweils zurück zum Start joggen/gehen, 3 bis 4 Minuten Pause oder joggen zu ebenem Gelände, 6 bis 8 x 400m bei Halbmarathon- bis 10-km-Pace (oder 85 bis 90% der max. Herzfrequenz) mit jeweils 60 bis 75 Sekunden Pause.<br>* Wiederhole in Woche 2 die 400-m-Intervalle 10-12 mal	2 bis 3 km, 5 Minuten Dehnen
**Donnerstag**	Ruhetag		
**Freitag**	5 Minuten aktives Dehnen	Lockeres Laufen bei 60 bis 65% der max. Herzfrequenz – 30 bis 40 Minuten	5 Minuten Dehnen
**Samstag**	2 bis 3 km, 5 Minuten aktives Dehnen	4 bis 6 x 100m-Sprints bei 90 bis 95% der Höchstleistung mit 30 - 45 Sekunden Pause, dann 3 Minuten Pause,<br>3 bis 6km Tempolauf bei Marathon-Pace (80% der max. Herzfrequenz)<br>* erhöhe den Tempolauf in Woche 2 auf 5-8km	2 bis 3 km, 5 Minuten Dehnen
**Sonntag**	Ruhetag		

Trainingsplan X English

Week 1-2	Warm-up	Workout Description	Cool-down
**Monday**	5 minutes active stretching	Easy running at 60 to 65% of max heart rate – 20 to 30 minutes	5 minutes stretching
**Tuesday**	5 minutes active stretching	Easy running or cross-training at 60 to 65% of max heart rate – 20 to 30 minutes or rest day	5 minutes stretching

**Wednesday**	2 to 3 km, 5 minutes active stretching	6 to 8 x 100m hill sprints, jogging/walking back for recovery, 3 to 4 minutes rest, or jog on flat terrain, 6 to 8 x 400m at half-marathon to 10k pace (or 85 to 90% of max heart rate) with 60 to 75 seconds rest. *Repeat 400m intervals 10-12 times in week 2	2 to 3 km, 5 minutes stretching
**Thursday**	Rest day		
**Friday**	5 minutes active stretching	Easy running at 60 to 65% of max heart rate – 30 to 40 minutes	5 minutes stretching
**Saturday**	2 to 3 km, 5 minutes active stretching	4 to 6 x 100m sprints at 90 to 95% of max effort with 30-45 seconds rest, then 3 minutes rest, 3 to 6 km tempo run at marathon pace (80% of max heart rate) *increase tempo run to 5-8 km in week 2	2 to 3 km, 5 minutes stretching
**Sunday**	Rest day		

Trainingsplan Y German

Woche 1-2	Aufwärmen	Workout Beschreibung	Abkühlen
**Montag**	10 Minuten leichtes Laufen	Easy Run: 3km in gemütlichem Tempo (60-70% deiner maximalen Herzfrequenz)	5-10 Minuten Gehen oder Dehnen
**Dienstag**	10 Minuten leichtes Laufen	Intervalltraining: 4x400 Meter schnelles Laufen (80-90% deiner maximalen Herzfrequenz) mit 2 Minuten Gehpause dazwischen	5-10 Minuten Gehen oder Dehnen
**Mittwoch**	Ruhetag oder leichtes Crosstraining (z.B. Radfahren oder Schwimmen		
**Donnerstag**	10 Minuten leichtes Laufen	Tempo Run: 5km moderates Tempo (70-80% deiner maximalen Herzfrequenz)	5-10 Minuten Gehen oder Dehnen
**Freitag**	Ruhetag oder leichtes Crosstraining		
**Samstag**	10 Minuten leichtes Laufen	Long Run: 8km in gemütlichem tempo (60-70% deiner maximalen Herzfrequenz)	5-10 Minuten Gehen oder Dehnen
**Sonntag**	Ruhetag		

Trainingsplan Y English

Week 1-2	Warm-up	Workout Description	Cool-down
**Monday**	10 minutes easy running	Easy Run: 3 km at a comfortable pace (60-70% of your maximum heart rate)	5-10 minutes walking or stretching
**Tuesday**	10 minutes easy running	Interval Training: 4x400 meters of fast running (80-90% of your maximum heart rate) with 2 minutes walking break in between	5-10 minutes walking or stretching
**Wednesday**	Rest day or light cross-training (e.g. cycling or swimming)		

**Thursday**	10 minutes easy running	Tempo Run: 5 km at a moderate pace (70-80% of your maximum heart rate)	5-10 minutes walking or stretching
**Friday**	Rest day or light crosstraining		
**Saturday**	10 minutes easy running	Long Run: 8 km at a comfortable pace (60-70% of your maximum heart rate)	5-10 minutes walking or stretching
**Sunday**	Rest day		
